# Supplementary material for: Fluorinated chlorin chromophores for red-light-driven CO2 reduction
Source: Nat Commun. 2024 Jul 8;15:5704. doi: 10.1038/s41467-024-50084-8 (PMC11231220; doi:10.1038/s41467-024-50084-8)
Supplement: Supplementary file 1 — Supplementary Information [file 41467_2024_50084_MOESM1_ESM.pdf]

Supplementary Information

Fluorinated chlorin chromophores for red-light-driven CO_2 reduction

Shuang Yang[1], Huiqing Yuan[1], Kai Guo[1], Zuting Wei[1], Mei Ming[1,2], Jinzhi Yi[1], Long Jiang[1], and Zhiji Han[1]*

[1]MOE Key Laboratory of Bioinorganic and Synthetic Chemistry, School of Chemistry, IGCME, Sun Yat-sen University, Guangzhou, 510275, China. [2]School of Materials Science and Engineering, Xihua University, Chengdu, 610039, China.

*Email: hanzhiji@mail.sysu.edu.cn.

Table of contents

Supplementary Figures 1-7 UV-vis absorption spectra of chromophores	S2-5
Supplementary Figure 8 Photo-flux of light sources	S5
Supplementary Table 1 Summary of molecular systems for CO_2RR with low-energy light	S6
Supplementary Table 2 Summary of heterogeneous systems for CO_2RR with low-energy light	S7
Supplementary Figure 9 TOF_{CO} for photocatalytic CO_2 reduction	S8
Supplementary Figures 10-13 Photocatalytic CO_2RR with F_{20}Ch	S9-10
Supplementary Table 3 Quantum yields of photocatalytic CO_2RR	S11
Supplementary Tables 4-5 Data for photocatalytic CO_2RR	S11
Supplementary Figure 14 GC-MS chromatograms of isotopic labeling experiments	S12
Supplementary Tables 6-7 Control experiments for photocatalytic CO_2RR	S12
Supplementary Figure 15 Photolysis performed with highly pure chemicals	S13
Supplementary Figure 16 Photocatalytic CO_2RR with or without Hg^0	S13
Supplementary Figure 17 Dynamic light scattering experiments	S14
Supplementary Figures 18-26 Electrochemical study of F_xChs	S14-20
Supplementary Figures 27-32 UV-vis spectra of F_xChs during photolysis	S21-24
Supplementary Figure 33 UV-vis spectra by keeping photolyzed solution in dark	S25
Supplementary Table 8 CO generation in the dark after photolysis	S25
Supplementary Figure 34 Photocatalytic CO_2RR of F_xChs at 730 nm	S26
Supplementary Figures 35-36 UV-vis spectra of photolysis solutions during CO_2RR.	S27-28
Supplementary Figure 37 Stability tests of photocatalytic systems.	S29
Supplementary Figures 38-43 UV-vis spectra of F_xTPP during photolysis	S29-34
Supplementary Figure 44 Photocatalytic CO_2RR for F_{20}Ch and F_{20}BC	S35
Supplementary Figures 45-46 UV-vis spectra of F_{20}BC during photolysis	S36-37
Supplementary Figures 47-48 Electrochemical study of BIH	S37-38
Supplementary Figure 49 Photocatalytic CO_2 reduction in DMF:TEA=4	S38
Supplementary Figures 50-54 UV-vis absorption spectra for mechanism study	S39-40
Supplementary Table 9 Crystal data and structure refinement for F_{20}BC	S41
Supplementary Table 10 Comparison of bond lengths between F_{20}BC and similar structures	S42
Supplementary Table 11 Molecular systems for CO_2 reduction with TEA additive.	S43
Supplementary Figure 55 ORTEP diagrams of F_{20}BC	S44
Supplementary Figures 56-58 Additional electrochemical data	S45-47
Supplementary Figures 59-60 Emission, excitation, and absorbance spectra of F_xChs	S48-49
Supplementary Figure 61 Emission decays of F_xChs	S50
Supplementary Figures 62-65 HRMS spectra of F_xChs	S51-52
Supplementary Figures 66-86 NMR spectra of chemicals used in photolysis	S53-65
Supplementary References	S66-67

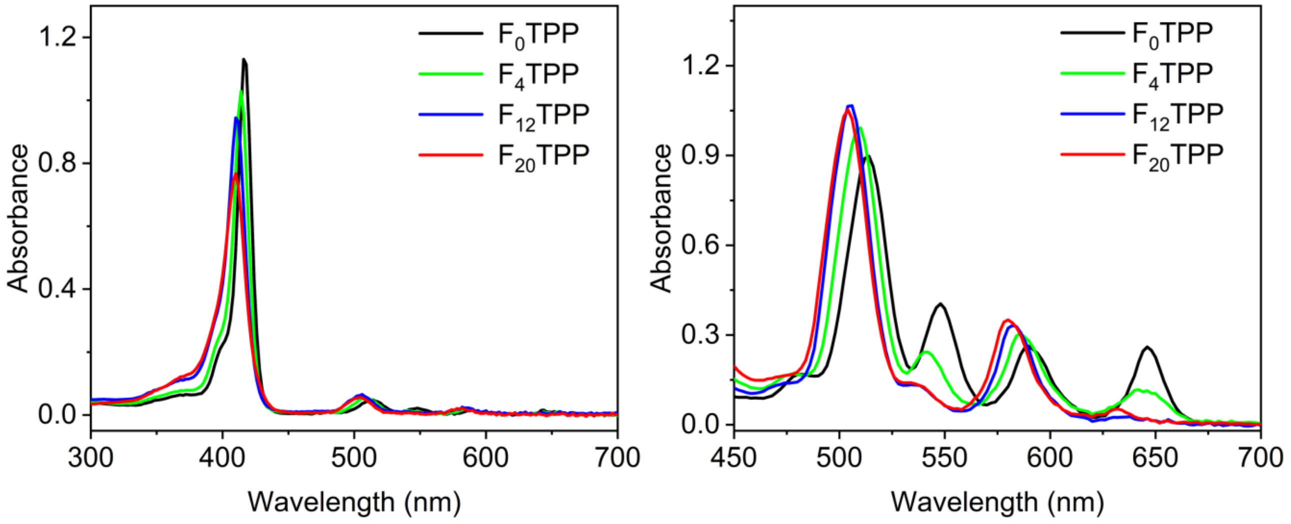

Supplementary Figure 1. UV-vis spectra. UV-vis absorption spectra of 2.5 µM (left) and 50 µM (right) F_0TPP (black), F_4TPP (green), $F_{12}TPP$ (blue), and $F_{20}TPP$ (red) in DMF in a quartz cuvette (10-mm path length).

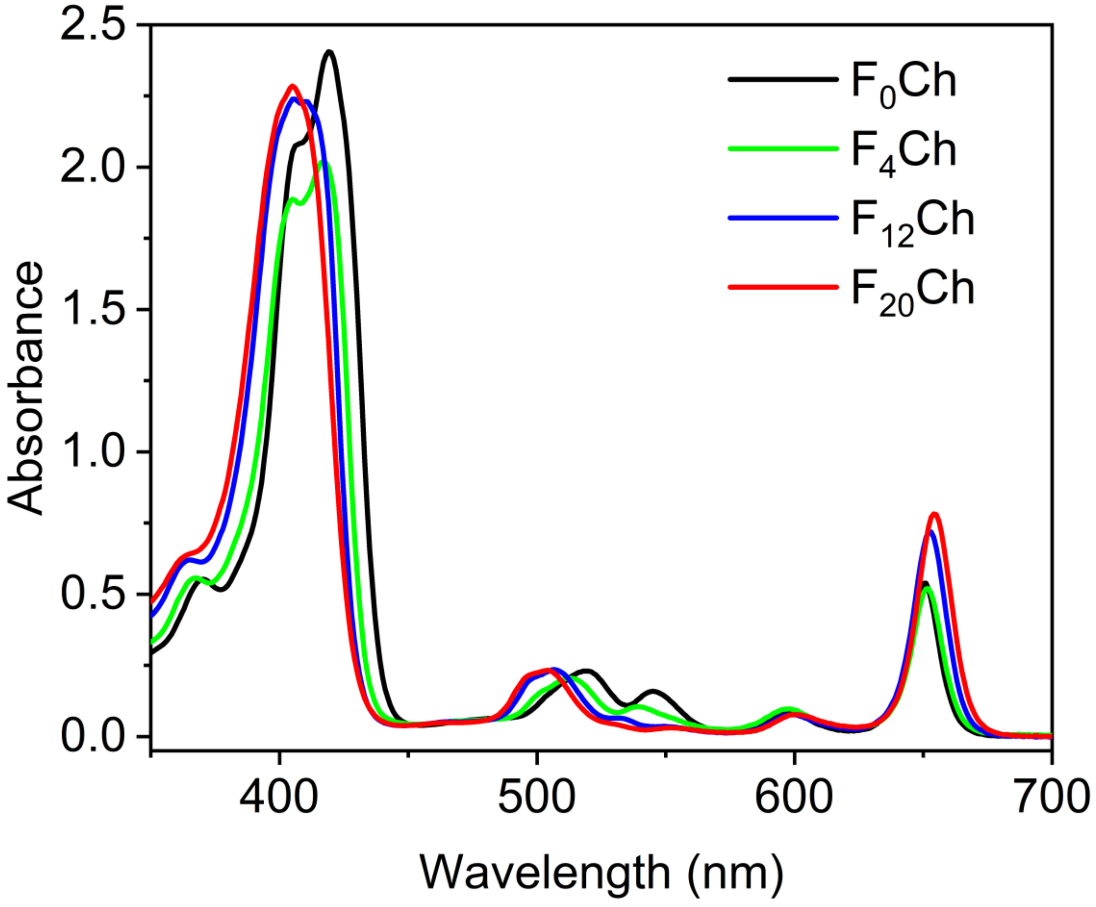

Supplementary Figure 2. UV-vis spectra. UV-vis absorption spectra of 15 µM F_0Ch (black), F_4Ch (green), $F_{12}Ch$ (blue), and $F_{20}Ch$ (red) in DMF in a quartz cuvette (10-mm path length).

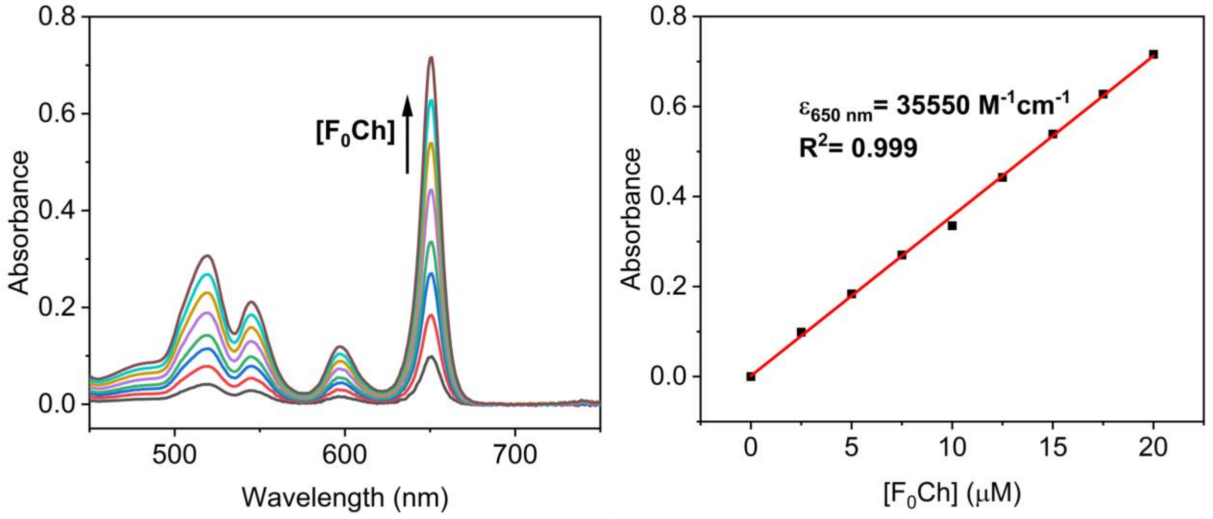

Supplementary Figure 3. UV-vis spectra. UV-vis absorption spectra of F_0Ch at different concentrations (2.5 to 20 μM) in DMF in a quartz cuvette (10-mm path length) (left); a linear plot of absorbance at 650 nm (right).

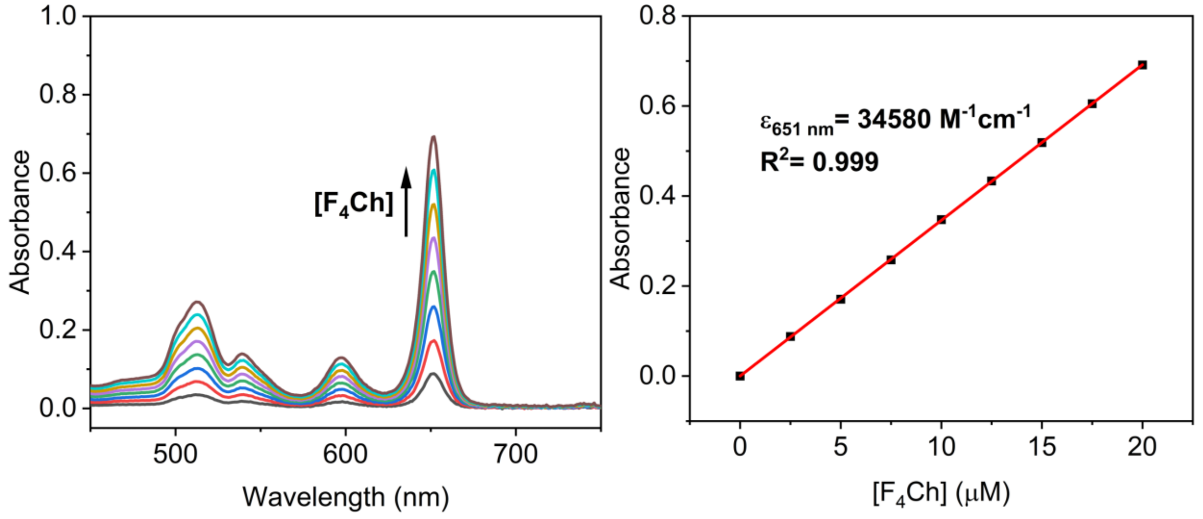

Supplementary Figure 4. UV-vis spectra. UV-vis absorption spectra of F_4Ch at different concentrations (2.5 to 20 μM) in DMF in a quartz cuvette (10-mm path length) (left); a linear plot of absorbance at 651 nm (right).

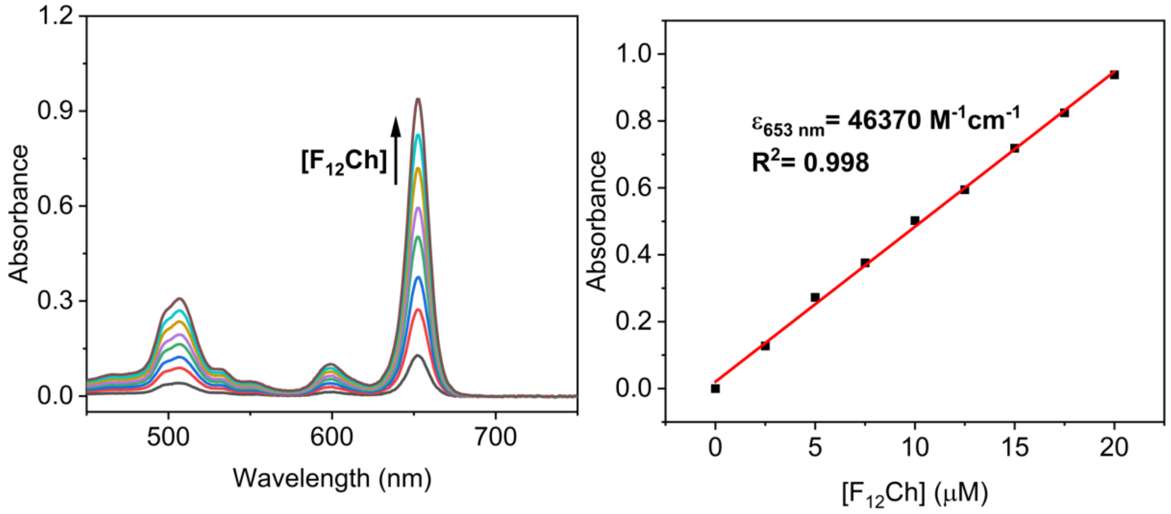

Supplementary Figure 5. UV-vis spectra. UV-vis absorption spectra of $F_{12}Ch$ at different concentrations (2.5 to 20 μM) in DMF in a quartz cuvette (10-mm path length) (left); a linear plot of absorbance at 653 nm (right).

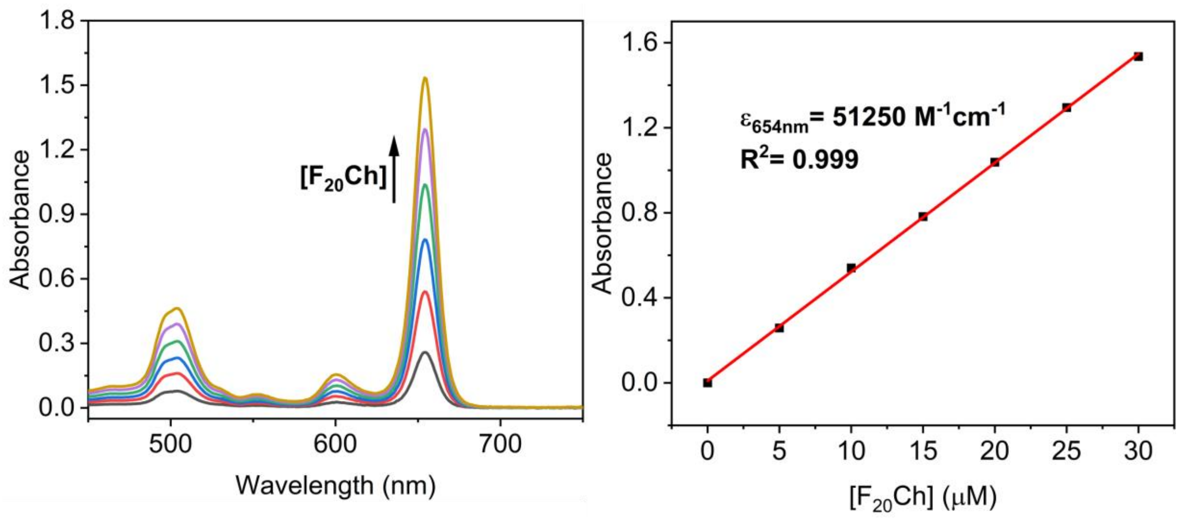

Supplementary Figure 6. UV-vis spectra. UV-vis absorption spectra of $F_{20}Ch$ at different concentrations (5 to 30 μM) in DMF in a quartz cuvette (10-mm path length) (left); a linear plot of absorbance at 654 nm (right).

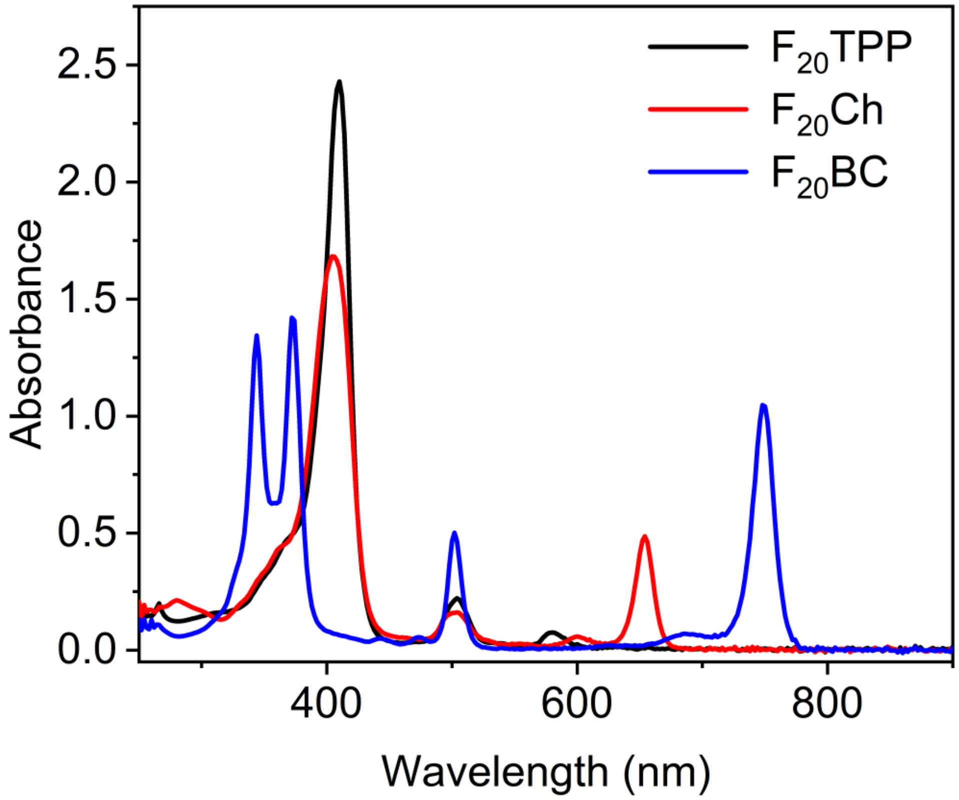

Supplementary Figure 7. UV-vis spectra. UV-vis absorption spectra of 10 μM F_{20}TPP (black), F_{20}Ch (red), and F_{20}BC (blue) in DMF in a quartz cuvette (10-mm path length).

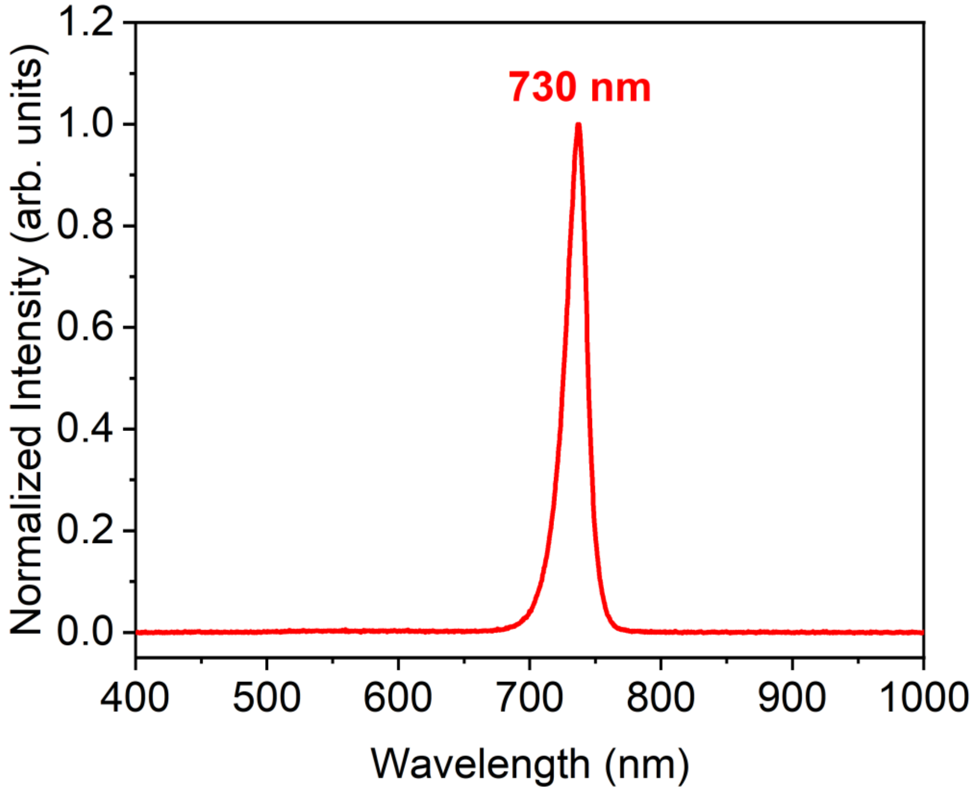

Supplementary Figure 8. Photo-flux of the 730 nm LED. (Photo-flux of the 630 nm LED has been reported from the reference[1]).

Supplementary Table 1 Summary of molecular systems for CO_2 reduction using low-energy light in the literature.

System	Major product	Solvent/Sacrificial donor	TON (time)	TOF (h^{-1})	ϕ_{CO} (%)	Light source (nm)	Ref
F_{20}Ch + FeTDHPP	CO	DMF/BIH	1842 (51 h)	203 (1.12×10^5 μmol g^{-1} h^{-1})	0.91	630	This work
F_{20}Ch + FeTDHPP	CO	DMF/BIH	510 (170 h)	8 (4.43×10^3 μmol g^{-1} h^{-1})	nr	730	This work
Os + Ru(CO)	HCOOH	DMA / BI(OH)H	81 (40 h)	nr	0.061 (480 nm)	725	2
Os + Ru(CO)	HCOOH	DMA / BI(OH)H	42 (12 h)	nr	nr	> 770	2
[ZnTMPyP]Cl_4 + Mn(bpy)$(CO)_3$Br	CO	H_2O/ AA	< 1 (~ 4 h)	< 0.1	2.67	625	3
Os(II)-Re(I)(Cl)	CO	DMF/TEOA=5 /BIH	1138 (20 h) (λ > 620 nm)	3.3 min^{-1} (λ > 420 nm)	12 (650 nm)	> 620	4

Supplementary Table 2: Summary of heterogeneous systems for CO_2 reduction using low-energy light in the literature.

System	Electron donor/Solvent	Major product	Light source (nm)	Evolution rates (μmol g^{-1} h^{-1})	ϕ_{CO} (%)	Ref
CoN porous atomic layers	Na_2S/H_2O	CO	800-2500	14.5	nr	5
$B_{13}P_2$ + $Co(bpy)_3^{2+}$	TEOA/DMF	CO	> 780	6.5	0.07 (810 nm)	6
V_S-$AgInS_2$	-/H_2O	CO	> 780	8.04	0.055 (790 nm)	7
P_3	BNAH/MeCN: TEOA=5	CO<br>CH_4	> 600	282.6<br>293.7	nr	8
CN + $[Co(bpy)_3]^{2+}$	TEOA/MeCN: TEOA=3:2	CO	660<br>730	nr	nr	9
BiOI	-/H_2O	CO<br>CH_4	≥ 700	0.119 μmol h^{-1}<br>0.021 μmol h^{-1}	0.02 ($CO+CH_4$) (700 nm)	10
m-NiAl-LDH	TEOA/ CH_3CN/TEOA /H_2O=3:1:1	CH_4	> 600	77.2	0.95 ($CO+CH_4$) (600 nm)	11
TNP-MOF	TEOA/MeCN	HCOOH	λ ≥ 730	6630 ± 242	2.03 (760 nm)<br>1.11 (808 nm)	12

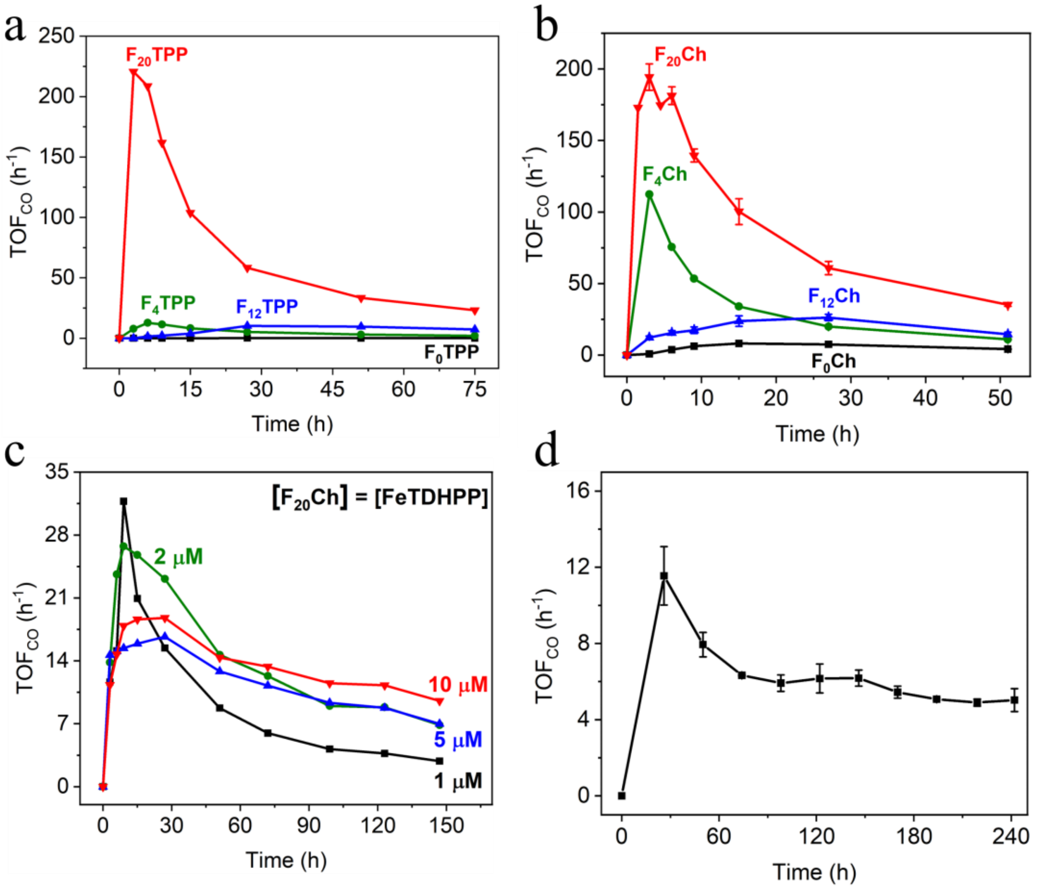

Supplementary Figure 9. Photocatalytic CO_2 reduction. a, TOF_{CO} for systems with different F_xTPP. b, TOF_{CO} for systems with different F_xCh. c, TOF_{CO} for systems with different initial [$F_{20}Ch$] and [FeTDHPP]. d, TOF_{CO} for stability of a system with $F_{20}Ch$. Catalytic conditions: (a-b) used 50 µM F_xTPP or F_xCh, 1.0 µM FeTDHPP, and 50 mM BIH; (c) used the same concentrations (1, 2, 5, 10 µM) of $F_{20}Ch$ and FeTDHPP, 50 mM BIH; (d) used 100 µM $F_{20}Ch$, 100 µM FeTDHPP, and 200 mM BIH. Experiments were in CO_2-saturated DMF (5.0 mL) at 20 °C using a light-emitting diode (LED) source (λ = 630 nm, 110 mW/cm^2). Error bars denote standard deviations.

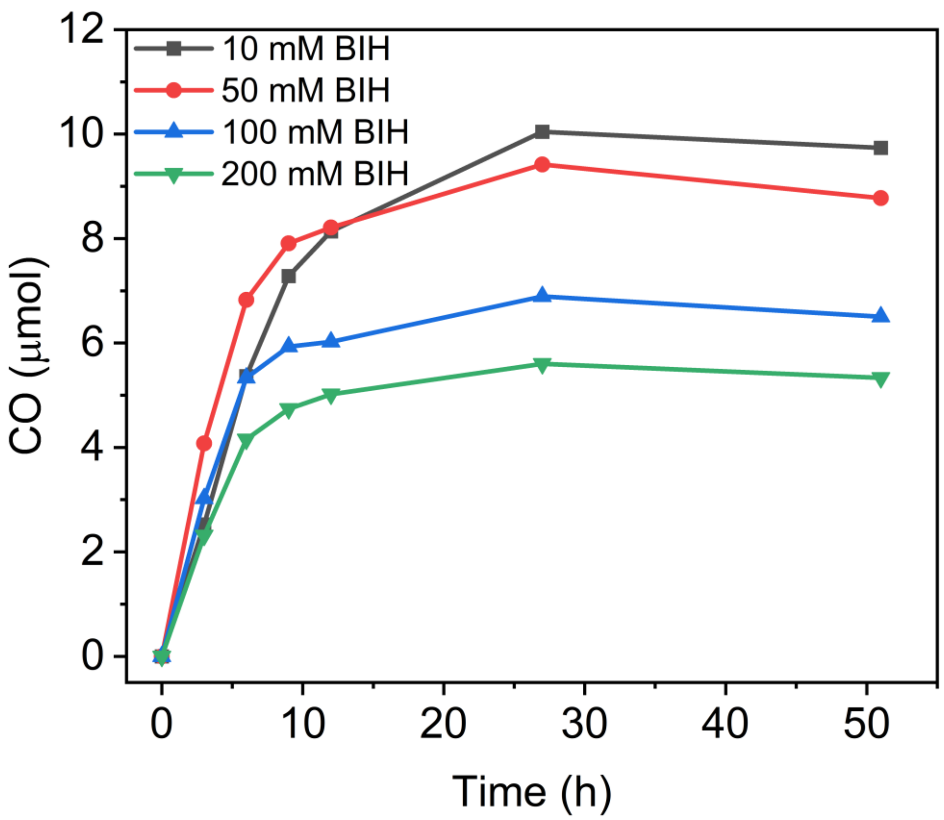

Supplementary Figure 10. Photocatalytic CO_2 reduction. Photocatalytic CO production in CO_2-saturated DMF solution containing 50 μM $F_{20}Ch$ and 1.0 μM FeTDHPP at different BIH concentrations under red LED (λ = 630 nm, 110 mW/cm^2) at 293 K.

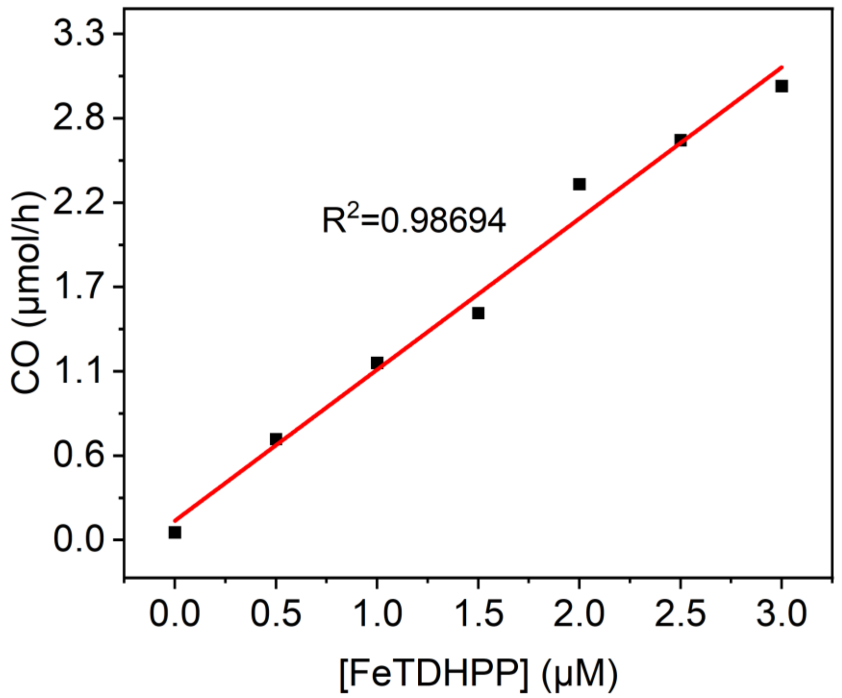

Supplementary Figure 11. Photocatalytic CO_2 reduction. Plot of the initial rate of CO generation with respect to [FeTDHPP]. Photocatalytic CO_2 reduction in CO_2-saturated DMF solutions containing 100 μM $F_{20}Ch$, 20 mM BIH and with different initial [FeTDHPP] under red LED (λ = 630 nm, 110 mW/cm^2) at 293 K.

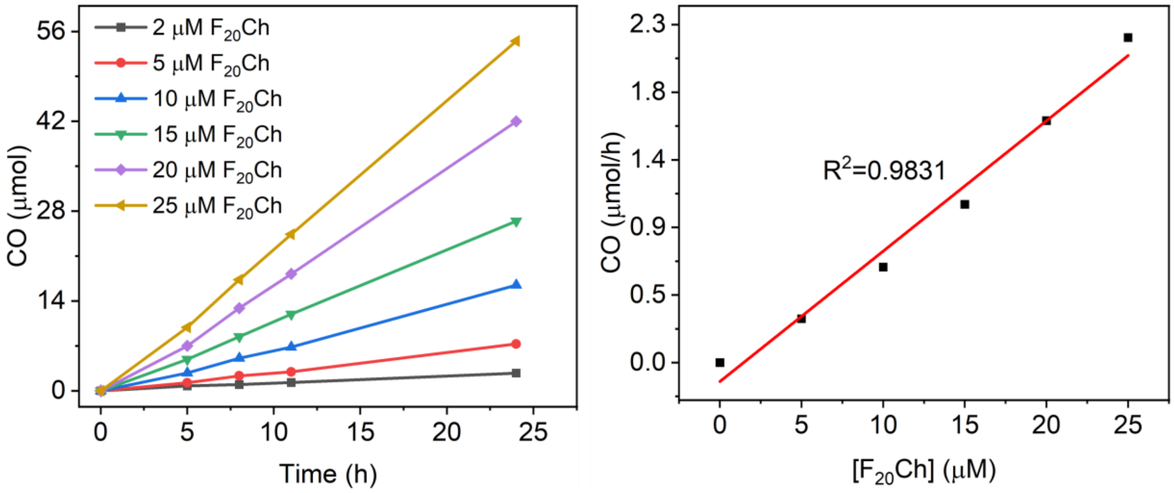

Supplementary Figure 12. Photocatalytic CO_2 reduction. Photocatalytic CO_2 reduction in CO_2-saturated DMF solutions containing 20 μM FeTDHPP, 20 mM BIH and with different initial [F_{20}Ch] from 2 to 25 μM under red LED (λ = 630 nm, 110 mW/cm^2) at 293 K (left); Plot of the initial rate of CO generation with respect to [F_{20}Ch] (right).

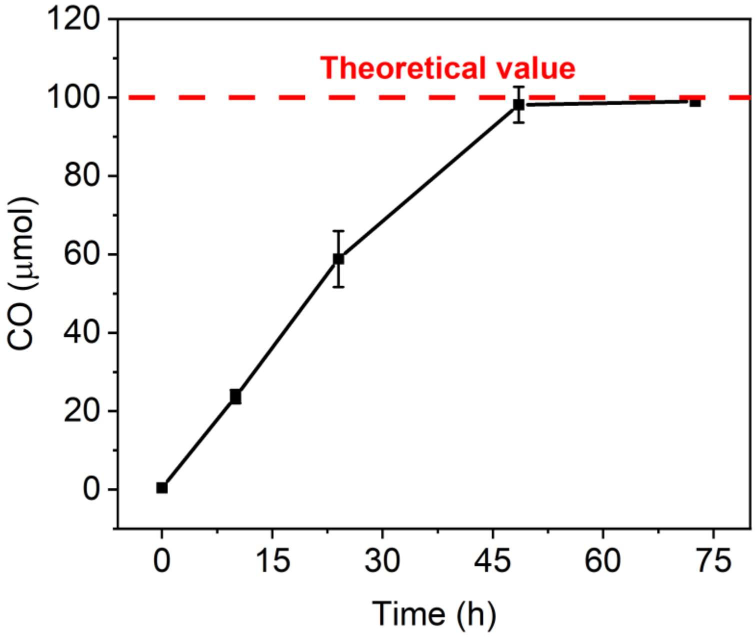

Supplementary Figure 13. Photocatalytic CO_2 reduction. Time profiles of photocatalytic CO_2 reduction in CO_2-saturated DMF solutions containing 100 μM FeTDHPP, 100 μM F_{20}Ch and 20 mM BIH under red LED (λ = 630 nm, 110 mW/cm^2) at 293 K. Error bars denote standard deviations based on at least three separated runs.

Supplementary Table 3. Quantum yields of CO production. Systems containing 50 µM F_xCh, 5 µM FeTDHPP, and 50 mM BIH in CO_2-saturated DMF under irradiation with red LEDs (λ = 630 nm, 110 mW/cm^2) for 15 h. Error bars denote standard deviations, based on at least three separated runs.

PS	CO (µmol)	Δ P (×10^{-4} W cm^{-2})	ϕ_{CO} (%)
F_0Ch	1.33 ± 0.026	27.7	0.05 ± 0.001
F_4Ch	28.78 ± 1.451	35.3	0.91 ± 0.046
$F_{12}Ch$	4.45 ± 0.328	105.0	0.05 ± 0.003
F_{20} Ch	44.98 ± 1.739	57.0	0.88 ± 0.034

Supplementary Table 4. Data for photocatalytic CO_2 reduction. Systems containing the same concentration of $F_{20}Ch$ and FeTDHPP with 50 mM BIH in CO_2-saturated DMF under irradiation with red LEDs (λ = 630 nm, 110 mW/cm^2) for 147 h at 293 K. a, 200 mM BIH, amount of CO and TON data collected for 242 h. Error bars denote standard deviations.

**[F_{20}Chlorin] = [FeTDHPP] (µM)**	**CO (µmol)**	**TON (CO)**	**TOF (h^{-1})**
1	2.1	420	31.8
2	10.1	1010	26.7
5	25.7	1028	16.7
10	70.2	1404	18.8
100[a]	608.1 ± 73.1	1216 ± 146	11.5 ± 1.5

Supplementary Table 5. Data for photocatalytic CO_2 reduction. Systems containing the 50 µM F_xTPP (x =0, 4, 12, 20) and 1 µM FeTDHPP and 50 mM BIH in CO_2 saturated DMF under irradiation with red LEDs (λ = 630 nm, 110 mW/cm^2) for 75 h at 293 K.

**chromophores**	**CO (µmol)**	**TON (CO)**	**TOF (h^{-1})**
F_0TPP	0.02	4	0.1
F_4TPP	0.75	150	12.7
$F_{12}TPP$	2.70	540	10.2
$F_{20}TPP$	8.70	1740	220.7

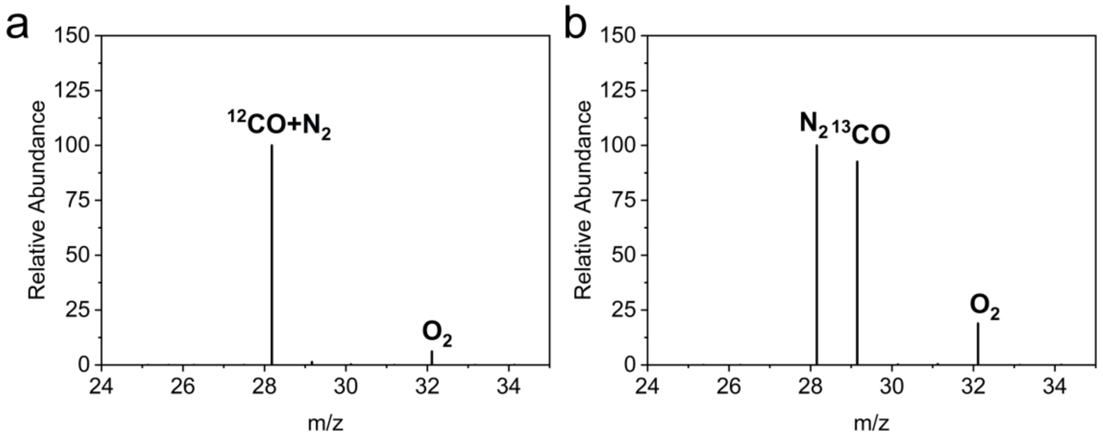

Supplementary Figure 14. GC/MS chromatograms of CO. GC-MS analyses of photocatalytic experiments under $^{12}CO_2$-saturated (a) and $^{13}CO_2$-saturated (b) in DMF solutions containing $F_{20}Ch$ (50 μM), FeTDHPP (1 μM) and BIH (50 mM) irradiated using red LED (λ = 630 nm, 110 mW/cm^2). The m/z signals of N_2 and O_2 were observed due to the presence of air.

Supplementary Table 6. Control experiments for photocatalytic CO_2 reduction[a]

Entry	Condition	CO (μmol)
1	No PS	0
2	No catalyst	trace
3	No BIH	trace
4	Under Ar instead of CO_2	0
5	No irradiation	0

[a] Standard conditions: a 5 mL CO_2-saturated DMF solution containing $F_{20}Ch$ (50 μM), FeTDHPP (1 μM) and BIH (50 mM) was irradiated using red LED (λ = 630 nm, 110 mW/cm^2) under a CO_2 atmosphere for 27 h.

Supplementary Table 7. Control experiments with inorganic salts.

Entry	Catalyst	CO (μmol)	H_2 (μmol)
1	FeTDHPP (1 μM)	8.2 ± 0.63	0
2	$Fe(NO_3)_3$ (10 μM)	0.1	trace
3	$Cu(NO_3)_2$ (10 μM)	0	0.17
4	$Ni(NO_3)_2$ (10 μM)	0.14	trace
5	$Co(NO_3)_3$ (10 μM)	0	trace
6	$RuCl_3$ (10 μM)	0.15	trace
7	$Pd(OAc)_2$ (10 μM)	0.17	16.3
8	$Fe(NO_3)_3$ (1μM) + TDHPP (1 μM)	0.12	0
9	$Cu(NO_3)_2$ (1μM) + TDHPP (1 μM)	0.11	0
10	$Ni(NO_3)_2$ (1μM) + TDHPP (1 μM)	0.13	0
11	$Co(NO_3)_3$ (1μM) + TDHPP (1 μM)	trace	0
12	$RuCl_3$ (1μM) + TDHPP (1 μM)	0.11	0
13	$Pd(OAc)_2$ (1 μM) + TDHPP (1 μM)	0	trace

Reaction conditions: a 5 mL CO_2-saturated DMF solution containing $F_{20}Ch$ (50 μM), BIH (50 mM) and metal catalyst was irradiated using red LED (λ = 630 nm, 110 mW/cm^2) under a CO_2 atmosphere for 27 h.

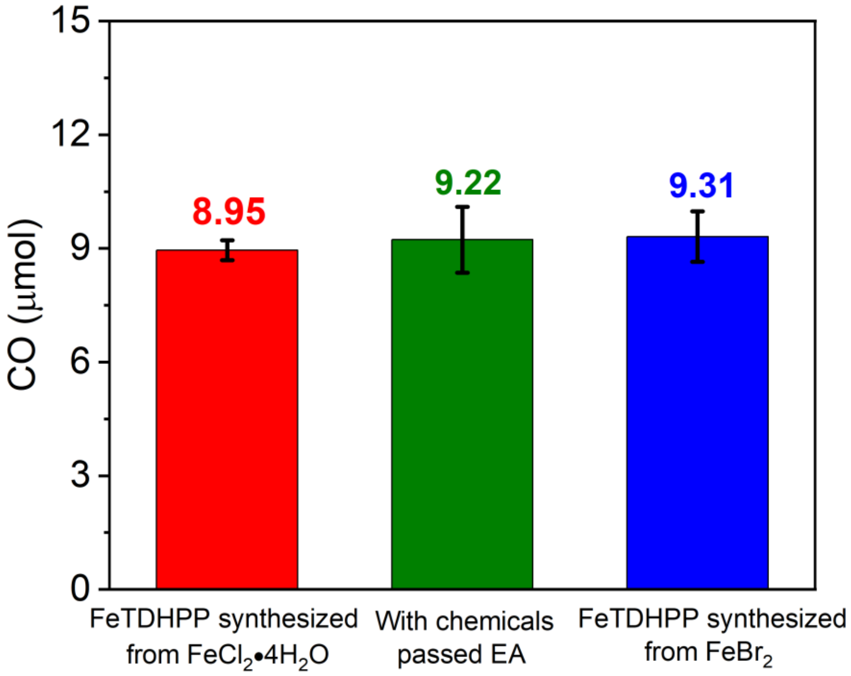

Supplementary Figure 15. Photocatalytic CO_2 reduction. Photocatalytic CO_2 reduction in CO_2-saturated DMF containing 50 µM $F_{20}Ch$, 1 µM FeTDHPP, and 50 mM BIH under red LED (λ = 630 nm, 110 mW/cm^2) at 293 K for 51 h. Error bars denote standard deviations, based on at least three separated runs. Purity of $FeCl_2 \cdot 4H_2O$ is 99.5%-101.0%. EA denotes elemental analysis. Purity of $FeBr_2$ is 99.995%.

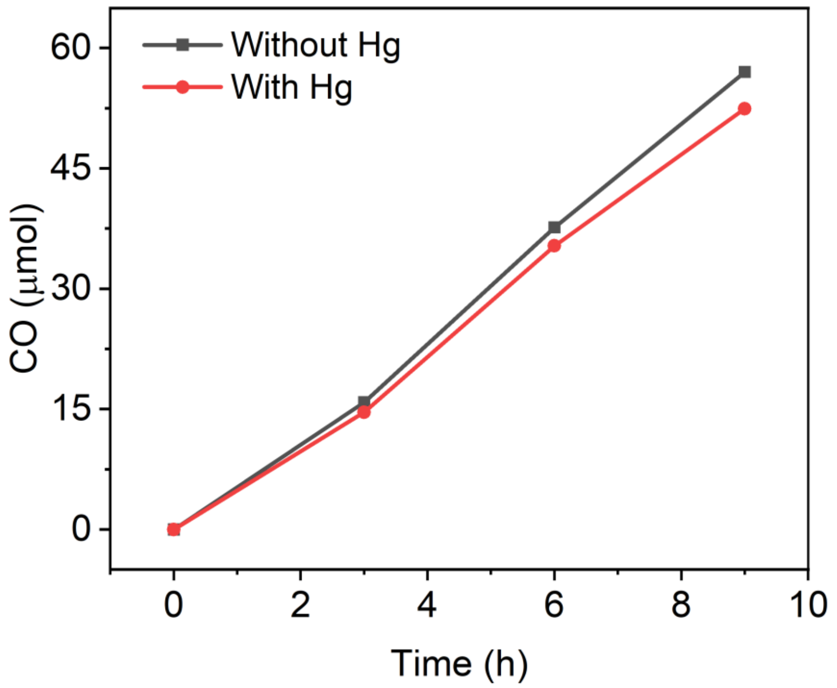

Supplementary Figure 16. Photocatalytic CO_2 reduction. Photocatalytic CO_2 reduction in the presence and absence of Hg (0.02 mL) in CO_2-saturated DMF solution containing 10 µM FeTDHPP, 50 µM $F_{20}Ch$, and 20 mM BIH under red LED (λ = 630 nm, 110 mW/cm^2) at 293 K.

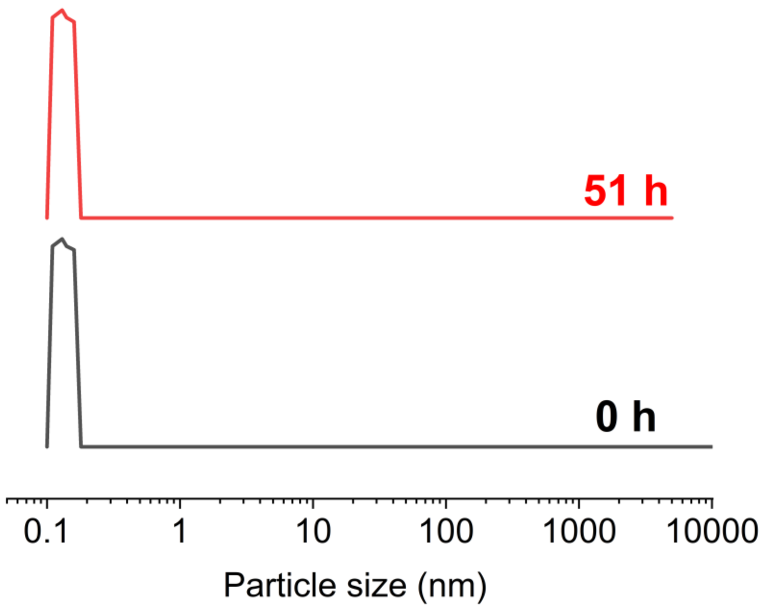

Supplementary Figure 17. Dynamic light scattering (DLS) measurement. Particle size distributions of a CO_2-saturated DMF solution containing $F_{20}Ch$ (50 µM), FeTDHPP (1 µM) and BIH (50 mM) determined by DLS measurement before and after irradiation using red LED (λ = 630 nm, 110 mW/cm^2).

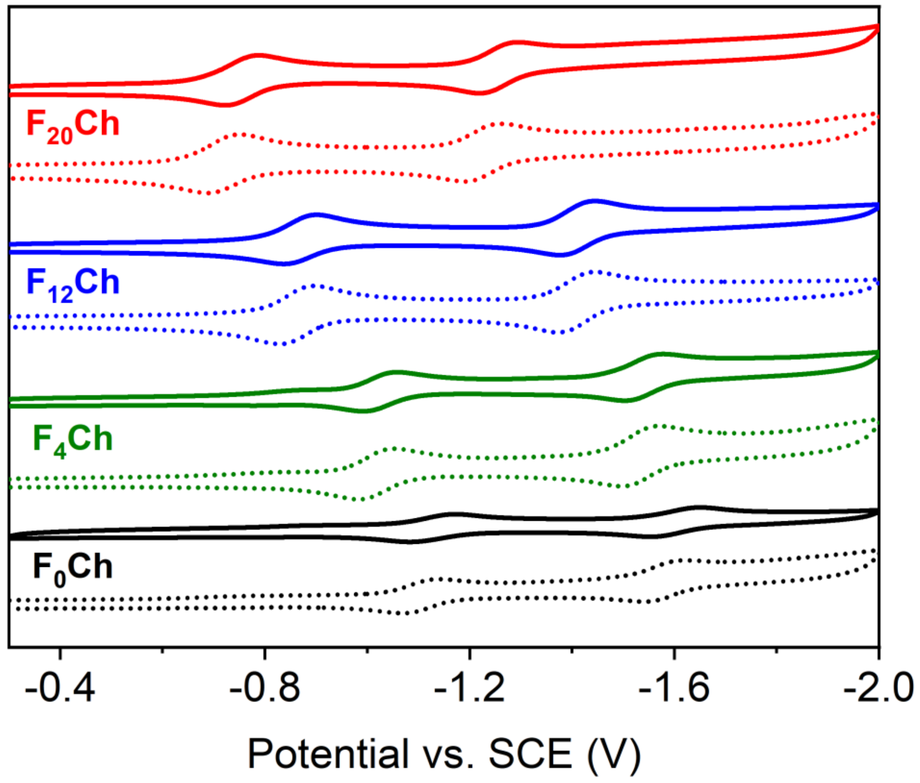

Supplementary Figure 18. Electrochemical study. Cyclic voltammograms of 0.25 mM F_0Ch (black), 0.5 mM F_4Ch (green), 0.5 mM $F_{12}Ch$ (blue), and 0.5 mM $F_{20}Ch$ (red) in DMF containing 0.1 M $TBAPF_6$ under Ar (solid) or under N_2 (short dot) at a scan rate of 0.1 V·s^{-1}.

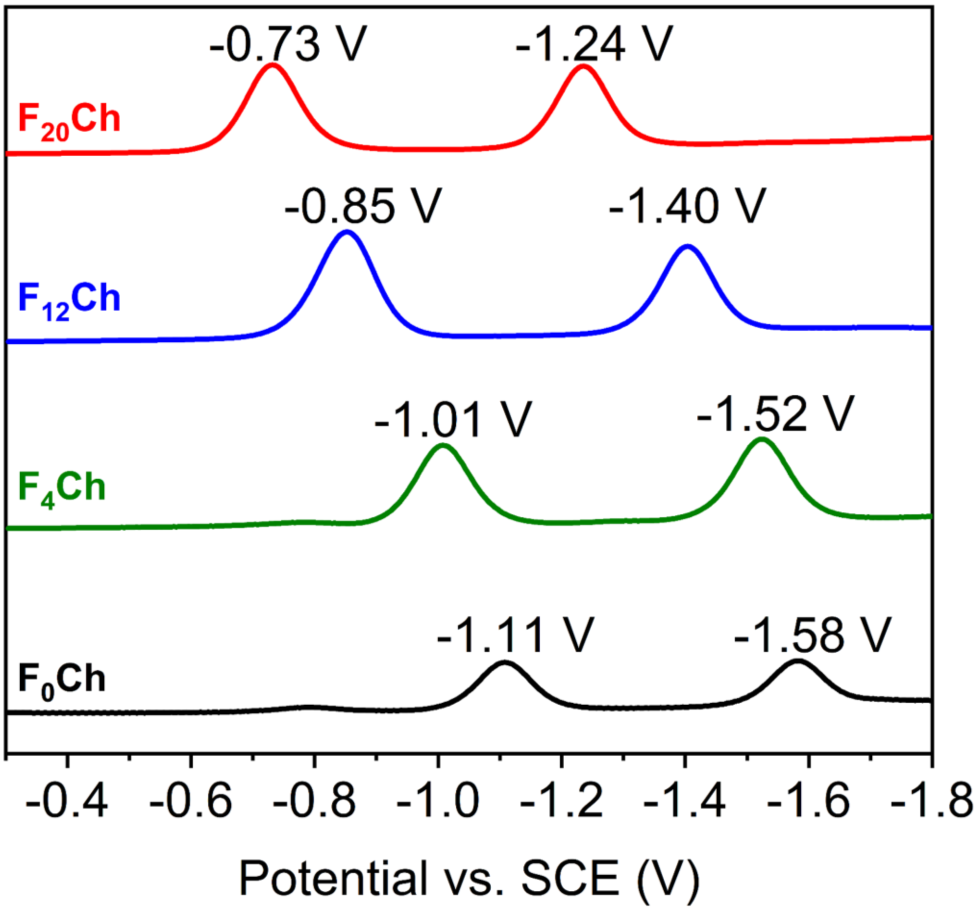

Supplementary Figure 19. Electrochemical study. Square wave voltammetry of 0.25 mM F_0Ch (black), 0.5 mM F_4Ch (green), 0.5 mM $F_{12}Ch$ (blue), and 0.5 mM $F_{20}Ch$ (red) in DMF containing 0.1 M $TBAPF_6$ at a scan rate of 0.1 $V{\cdot}s^{-1}$ under N_2.

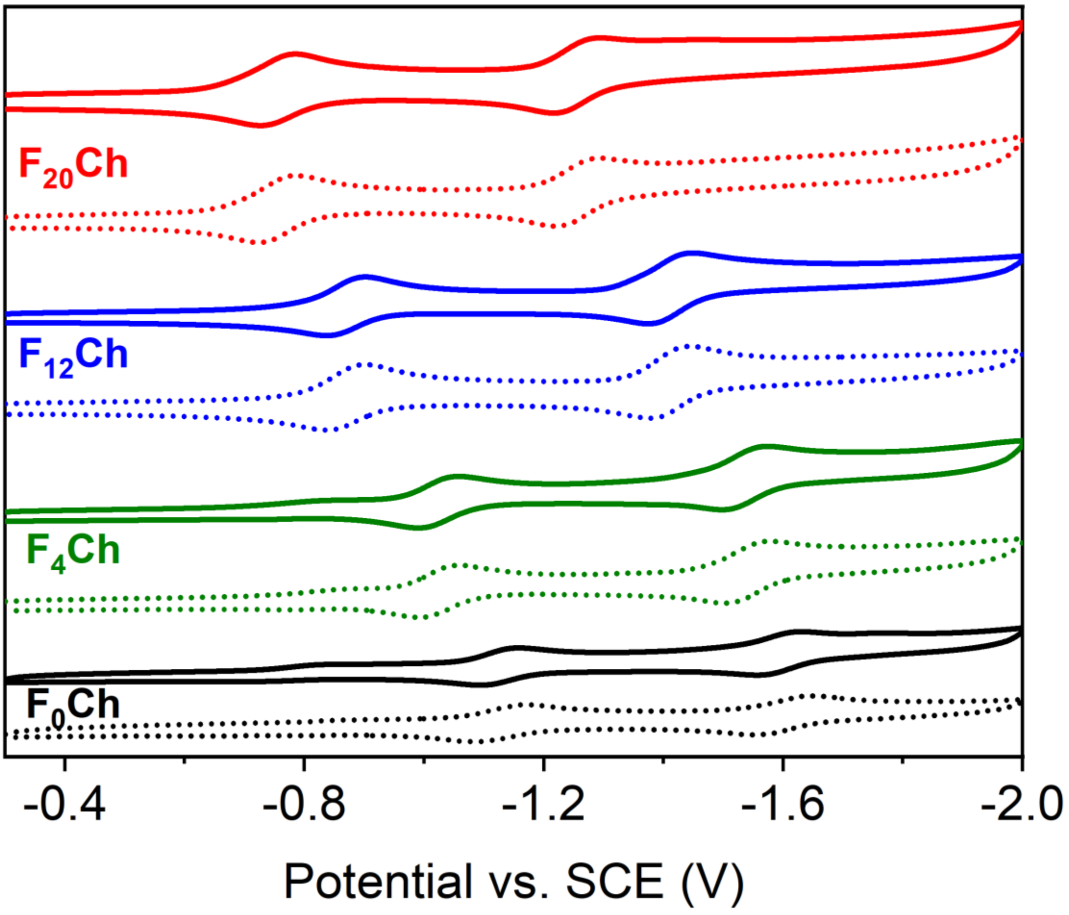

Supplementary Figure 20. Electrochemical study. Cyclic voltammograms of 0.25 mM F_0Ch (black), 0.5 mM F_4Ch (green), 0.5 mM $F_{12}Ch$ (blue), and 0.5 mM $F_{20}Ch$ (red) in DMF containing 0.1 M $TBAPF_6$ in the presence (solid) and absence (short dot) of 1% H_2O under Ar at a scan rate of 0.1 $V \cdot s^{-1}$.

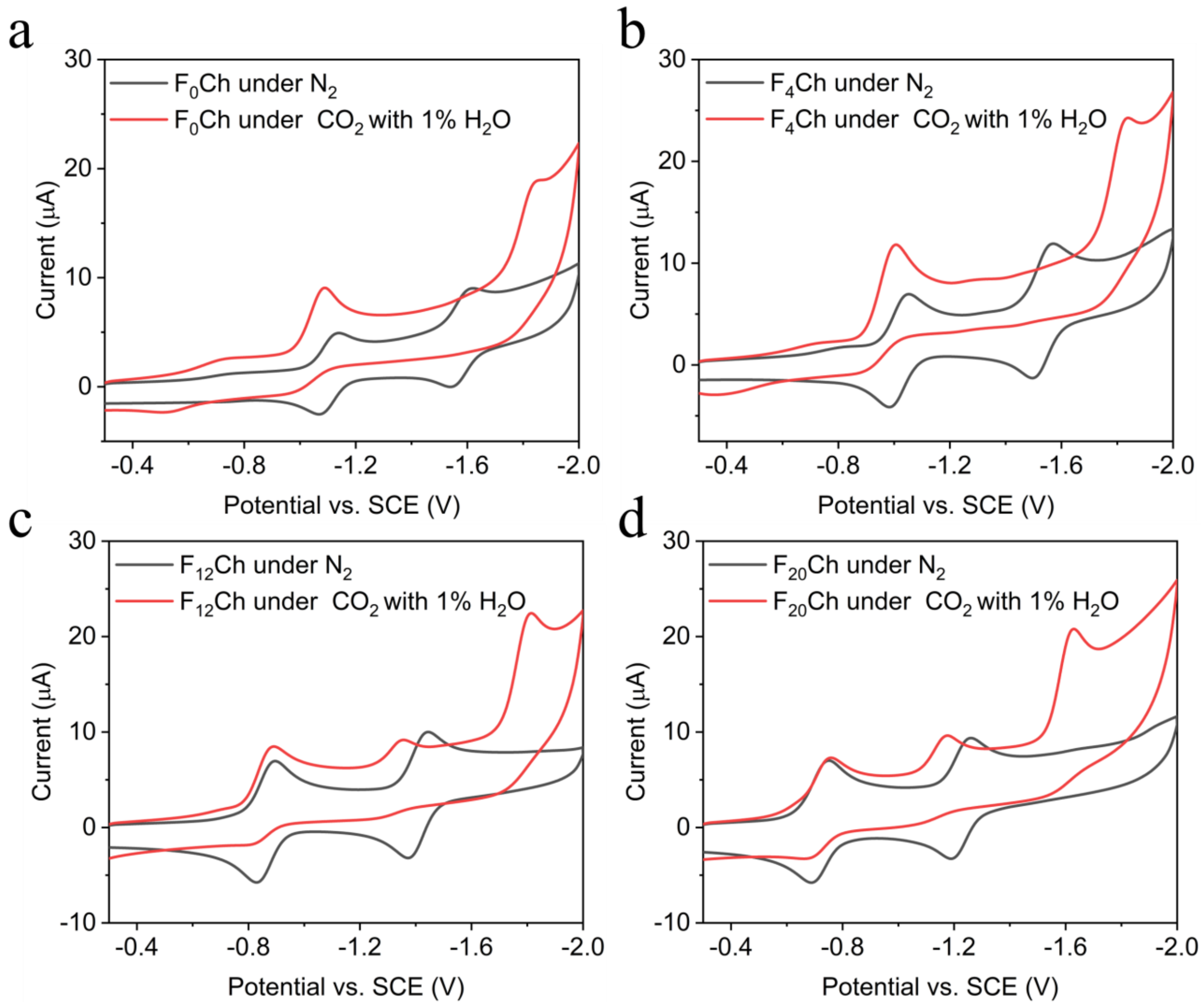

Supplementary Figure 21. Electrochemical study. Cyclic voltammograms of 0.25 mM F_0Ch (a), 0.5 mM F_4Ch (b), 0.5 mM $F_{12}Ch$ (c), and 0.5 mM $F_{20}Ch$ (d) in DMF containing 0.1 M $TBAPF_6$ at a scan rate of 0.1 $V\cdot s^{-1}$ under N_2 (black) and under CO_2 in the presence of 1% H_2O (red).

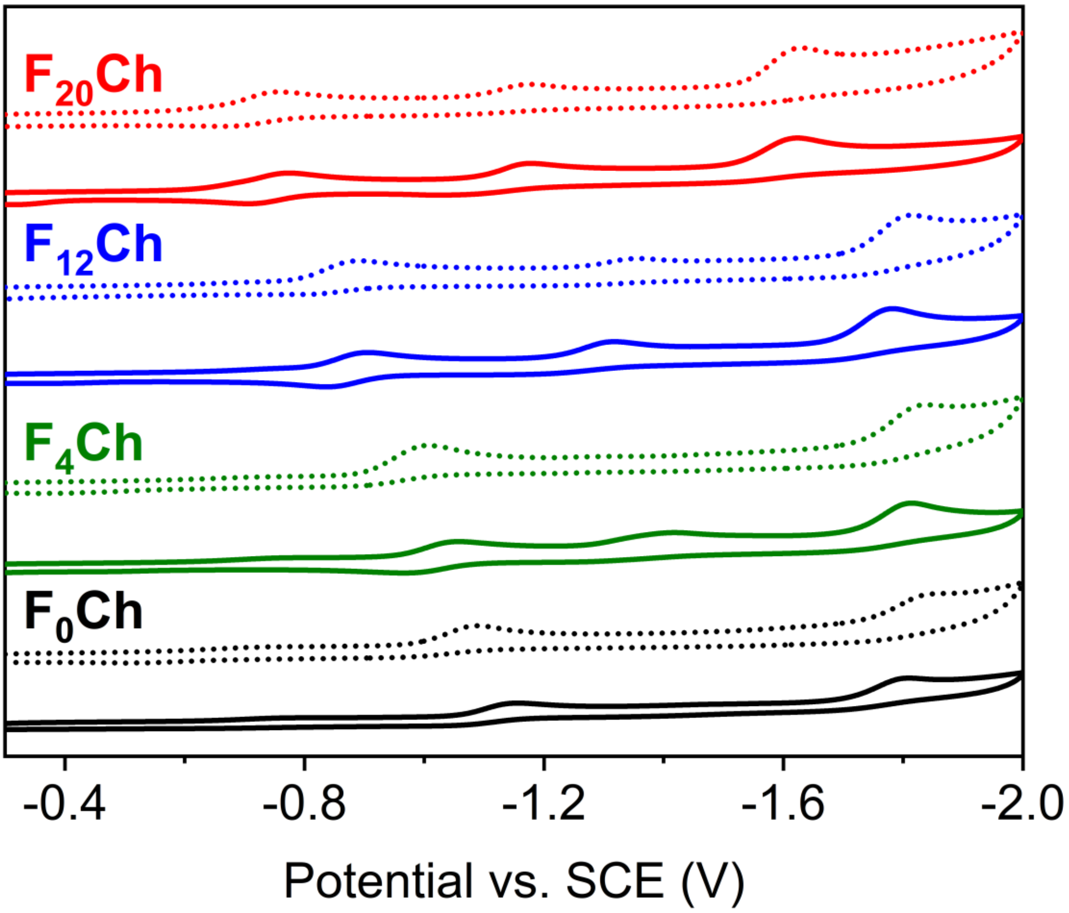

Supplementary Figure 22. Electrochemical study. Cyclic voltammograms of 0.25 mM F_0Ch (black), 0.5 mM F_4Ch (green), 0.5 mM $F_{12}Ch$ (blue), and 0.5 mM $F_{20}Ch$ (red) in DMF containing 0.1 M $TBAPF_6$ in the presence of 1.0 M TFE under N_2 (solid) and under CO_2 with 1% H_2O (short dot) at a scan rate of 0.1 $V \cdot s^{-1}$.

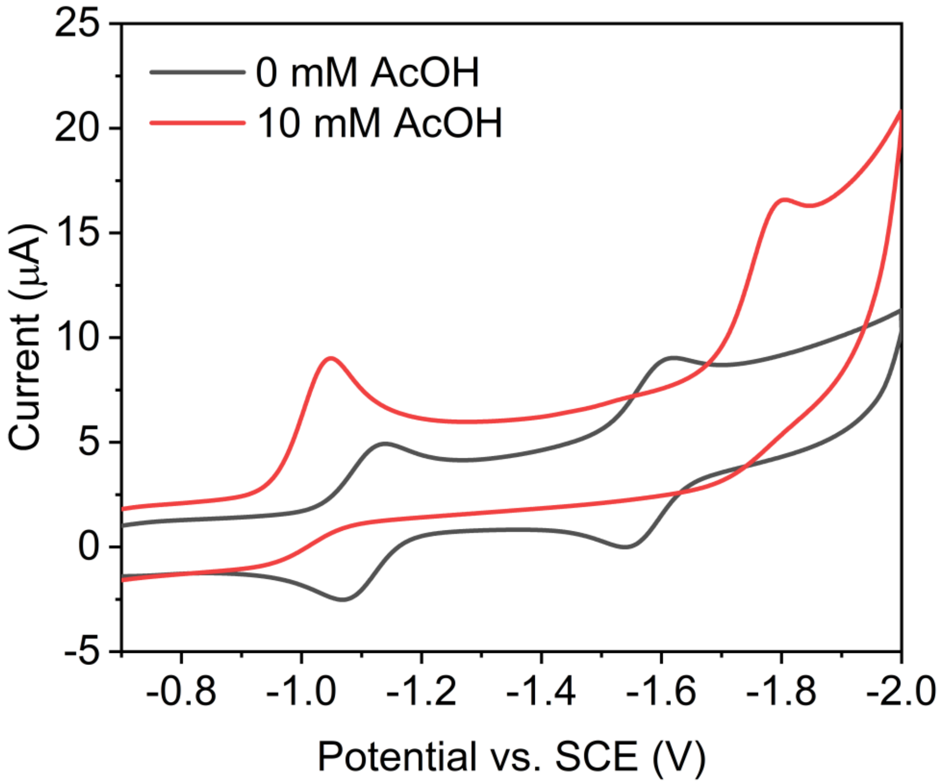

Supplementary Figure 23. Electrochemical study. Cyclic voltammograms of 0.25 mM F_0Ch in the absence and presence of 10 mM AcOH in DMF containing 0.1 M $TBAPF_6$ at a scan rate of 0.1 $V{\cdot}s^{-1}$ under N_2.

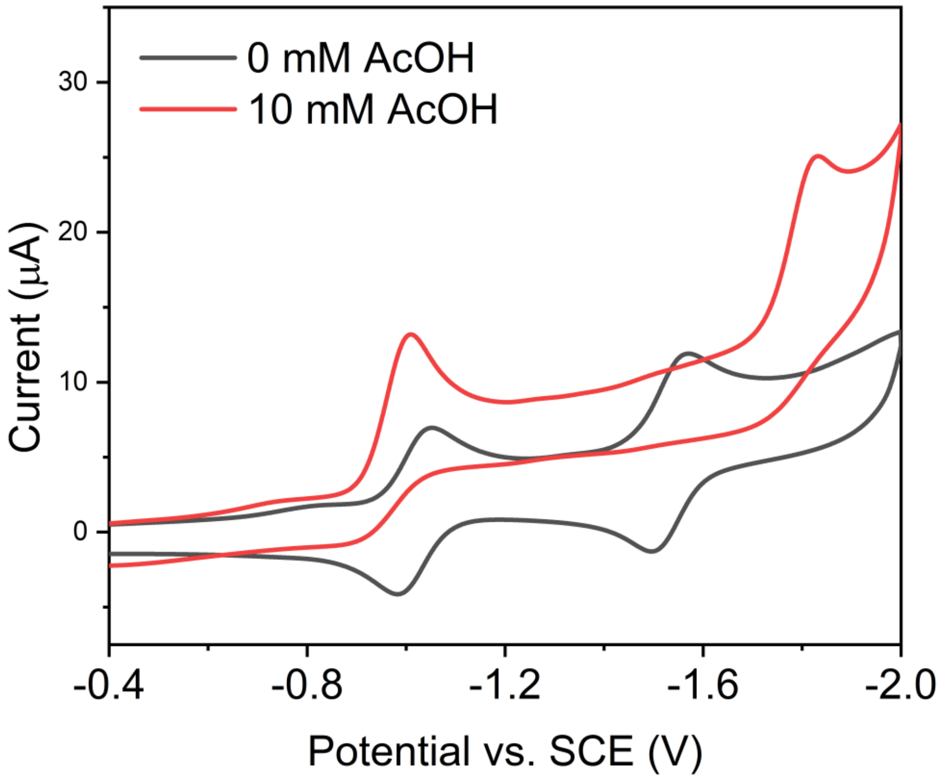

Supplementary Figure 24. Electrochemical study. Cyclic voltammograms of 0.5 mM F_4Ch in the absence and presence of 10 mM AcOH in DMF containing 0.1 M $TBAPF_6$ at a scan rate of 0.1 $V{\cdot}s^{-1}$ under N_2.

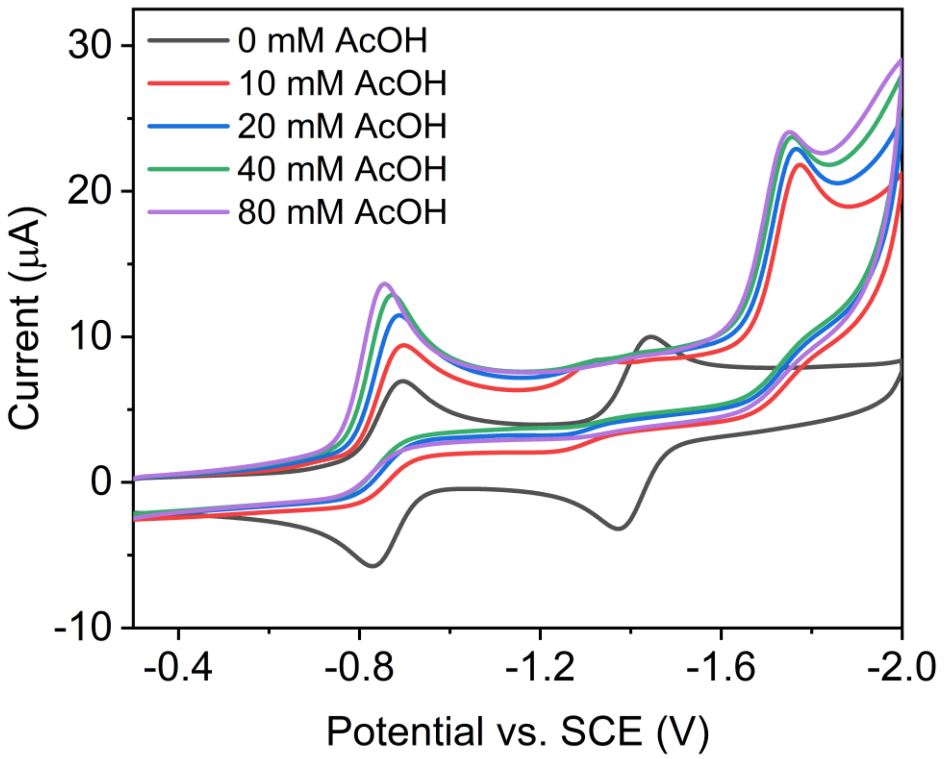

Supplementary Figure 25. Electrochemical study. Cyclic voltammograms of 0.5 mM $F_{12}Ch$ in the absence and presence of AcOH (up to 80 mM) in DMF containing 0.1 M $TBAPF_6$ at a scan rate of 0.1 $V \cdot s^{-1}$ under N_2.

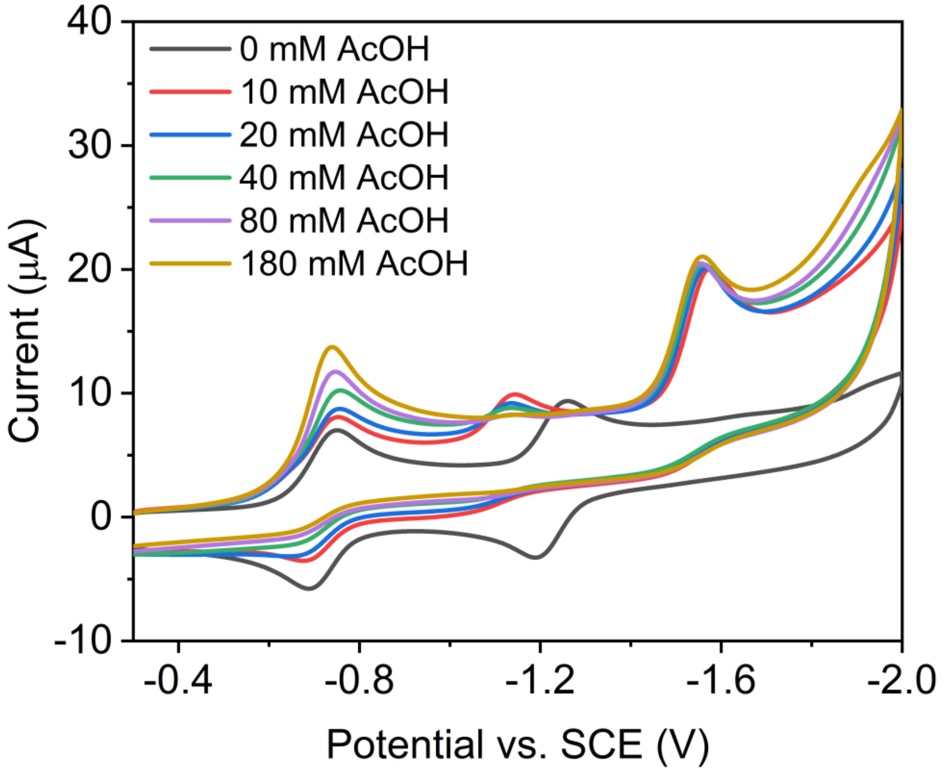

Supplementary Figure 26. Electrochemical study. Cyclic voltammograms of 0.5 mM $F_{20}Ch$ in the absence and presence of AcOH (up to 180 mM) in DMF containing 0.1 M $TBAPF_6$ at a scan rate of 0.1 $V \cdot s^{-1}$ under N_2.

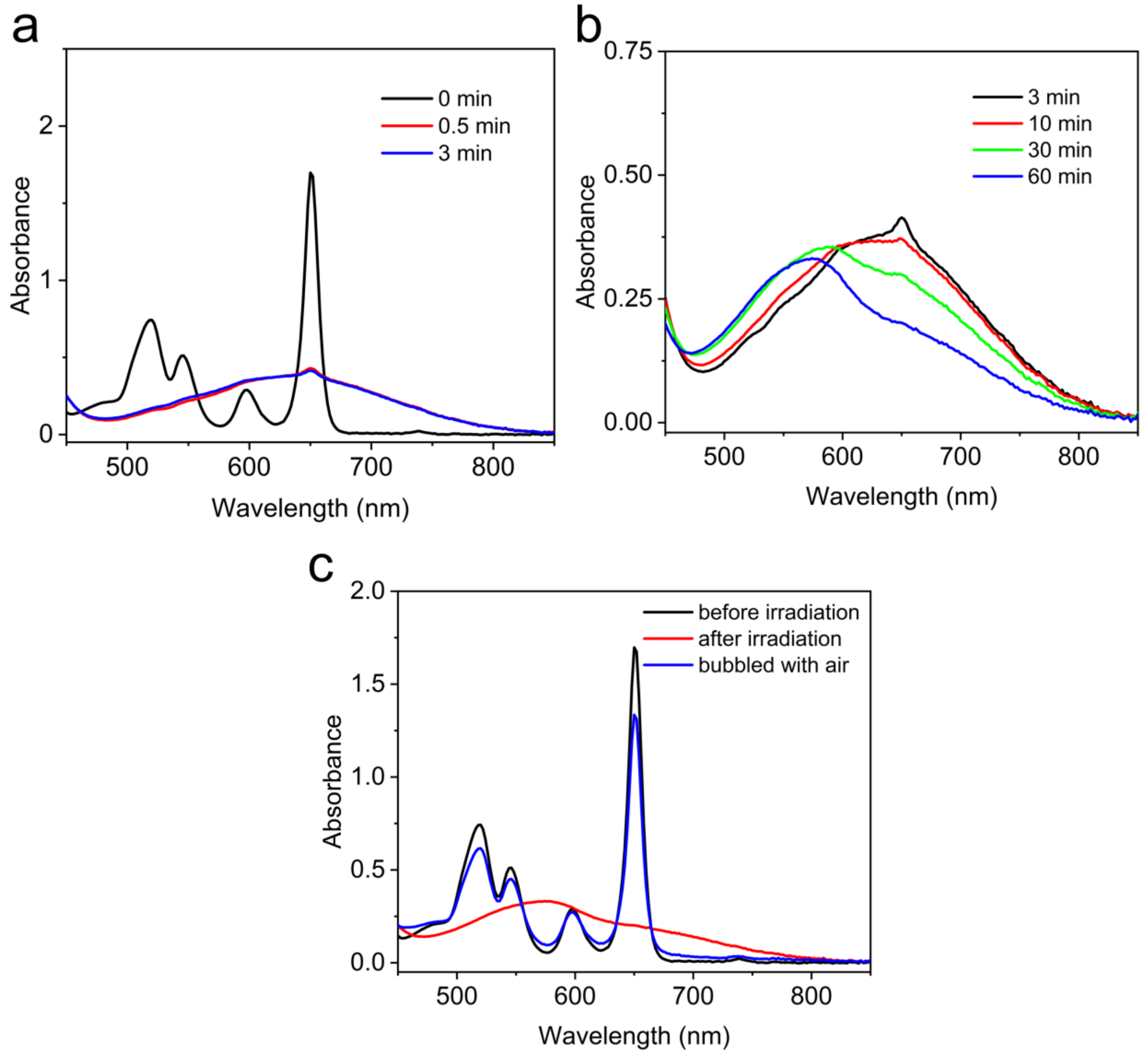

Supplementary Figure 27. UV-vis absorption spectra. UV-vis absorption spectra of systems containing 50 μM F_0Ch and 20 mM BIH in DMF under CO_2 upon irradiation with red LED light (λ = 630 nm, 110 mW/cm^2) in a quartz cuvette (10-mm path length). Irradiation time ranging from 0 to 3 min (a), and from 3 min to 60 min (b). The mixture was bubbled with air at 60 min (c). Note: 78% of the F_0Ch can be recovered by exposing the photolysis mixture to air.

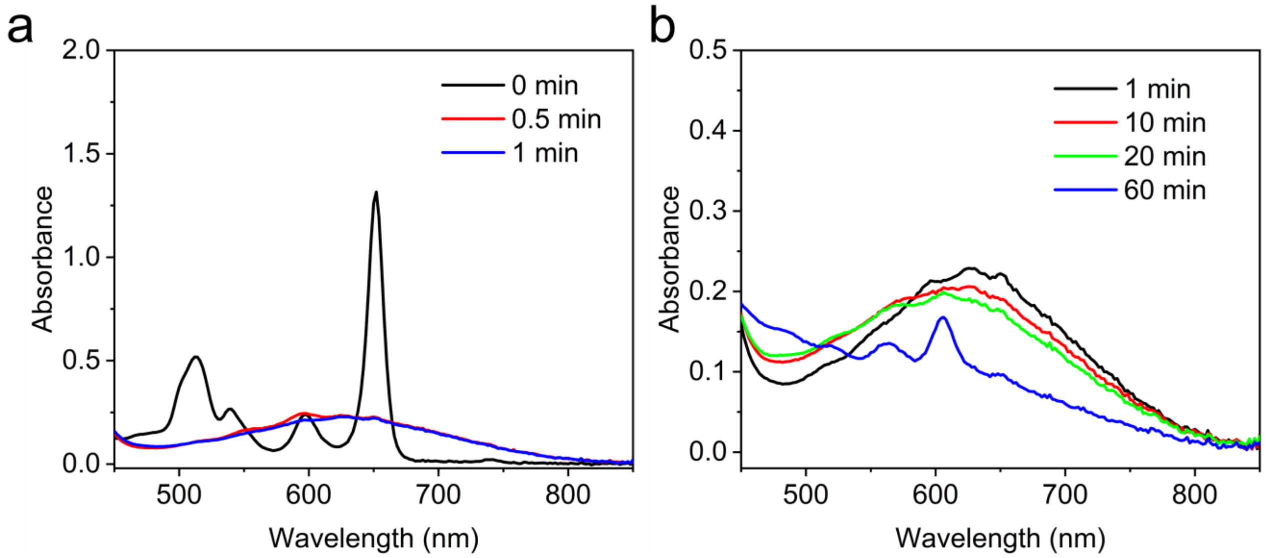

Supplementary Figure 28. UV-vis absorption spectra. UV-vis absorption spectra of systems containing 50 μM F_4Ch and 20 mM BIH in DMF under CO_2 upon irradiation with red LED light (λ = 630 nm, 110 mW/cm^2) in a quartz cuvette (10-mm path length). Irradiation time ranging from 0 to 1 min (a), and from 1 min to 60 min (b).

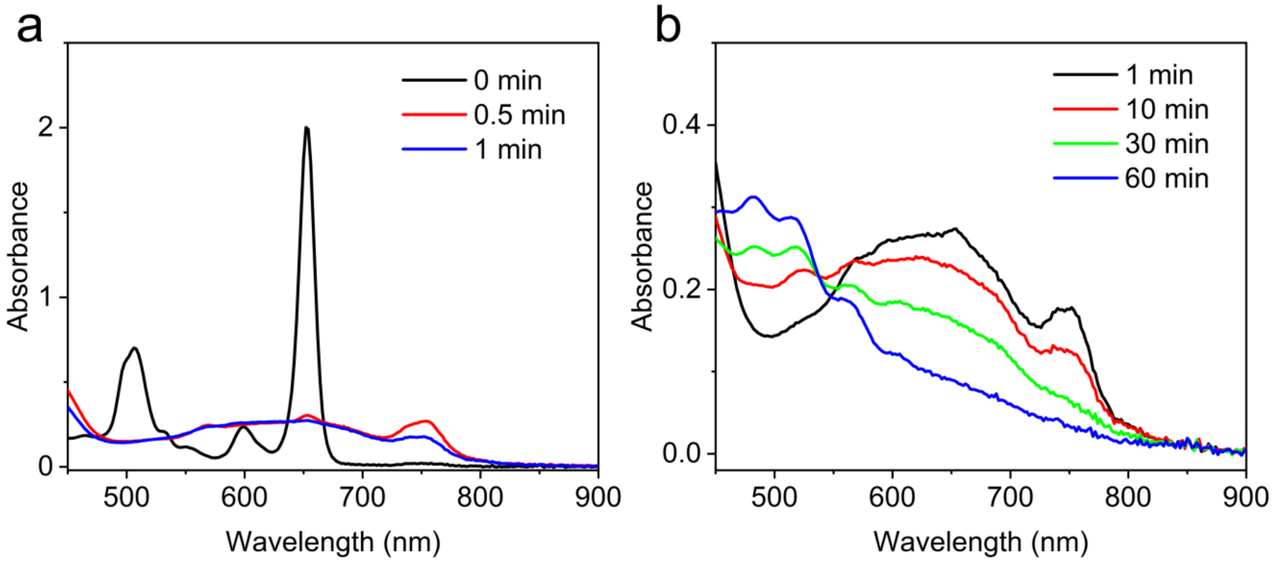

Supplementary Figure 29. UV-vis absorption spectra. UV-vis absorption spectra of systems containing 50 μM $F_{12}Ch$ and 20 mM BIH in DMF under CO_2 upon irradiation with red LED light (λ = 630 nm, 110 mW/cm^2) in a quartz cuvette (10-mm path length). Irradiation time ranging from 0 to 1 min (a), and from 1 min to 60 min (b).

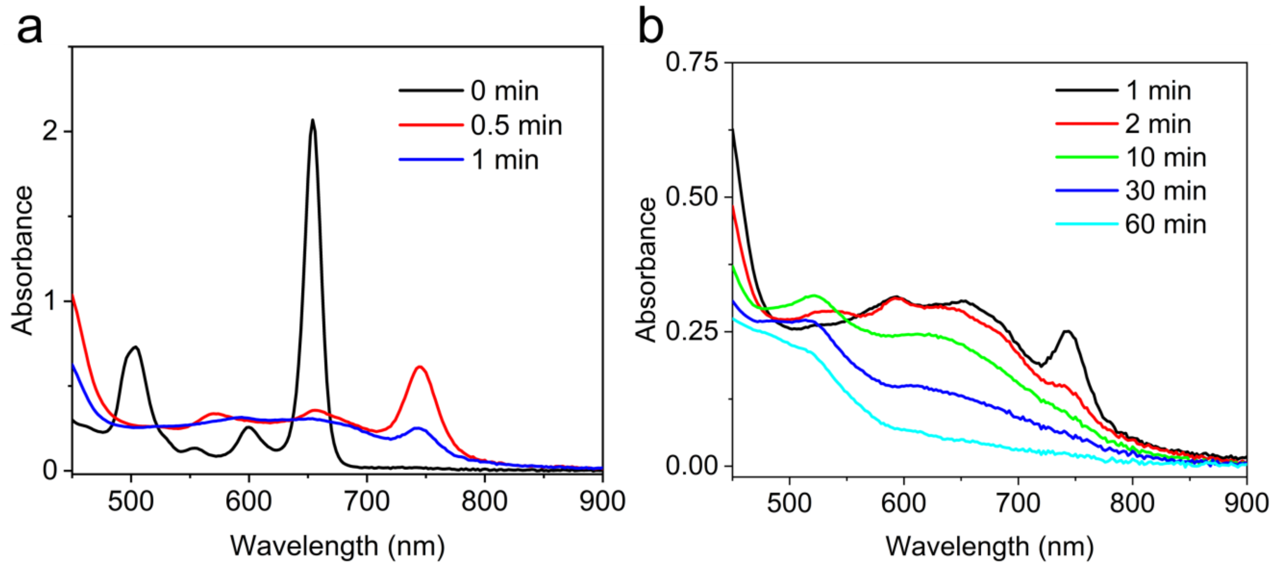

Supplementary Figure 30. UV-vis absorption spectra. UV-vis absorption spectra of systems containing 50 μM $F_{20}Ch$ and 20 mM BIH in DMF under CO_2 upon irradiation with red LED light (λ = 630 nm, 110 mW/cm^2) in a quartz cuvette (10-mm path length). Irradiation time ranging from 0 to 1 min (a), and from 1 min to 60 min (b).

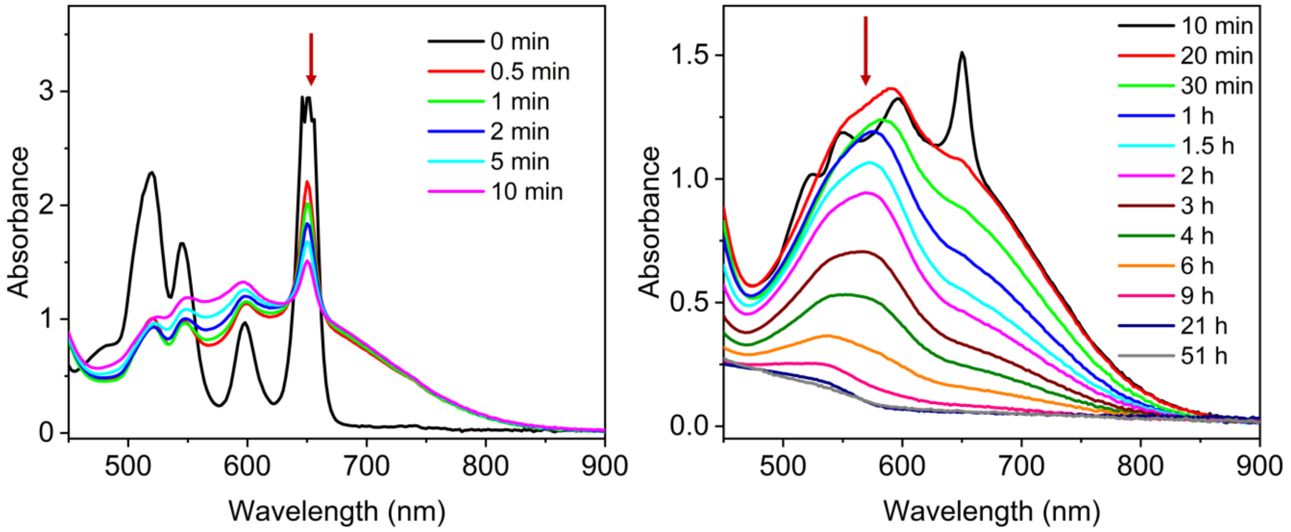

Supplementary Figure 31. UV-vis absorption spectra. UV-vis absorption spectra of CO_2-saturated DMF solution containing 50 μM F_0Ch, 1.0 μM FeTDHPP, and 50 mM BIH in the same photocatalytic flask (3.05-cm path length) used in our study for CO production. Irradiation time ranging from 0 to 10 min (left), and from 10 min to 51 h (right). Conditions: red LED light (λ = 630 nm, 110 mW/cm^2) at 293 K.

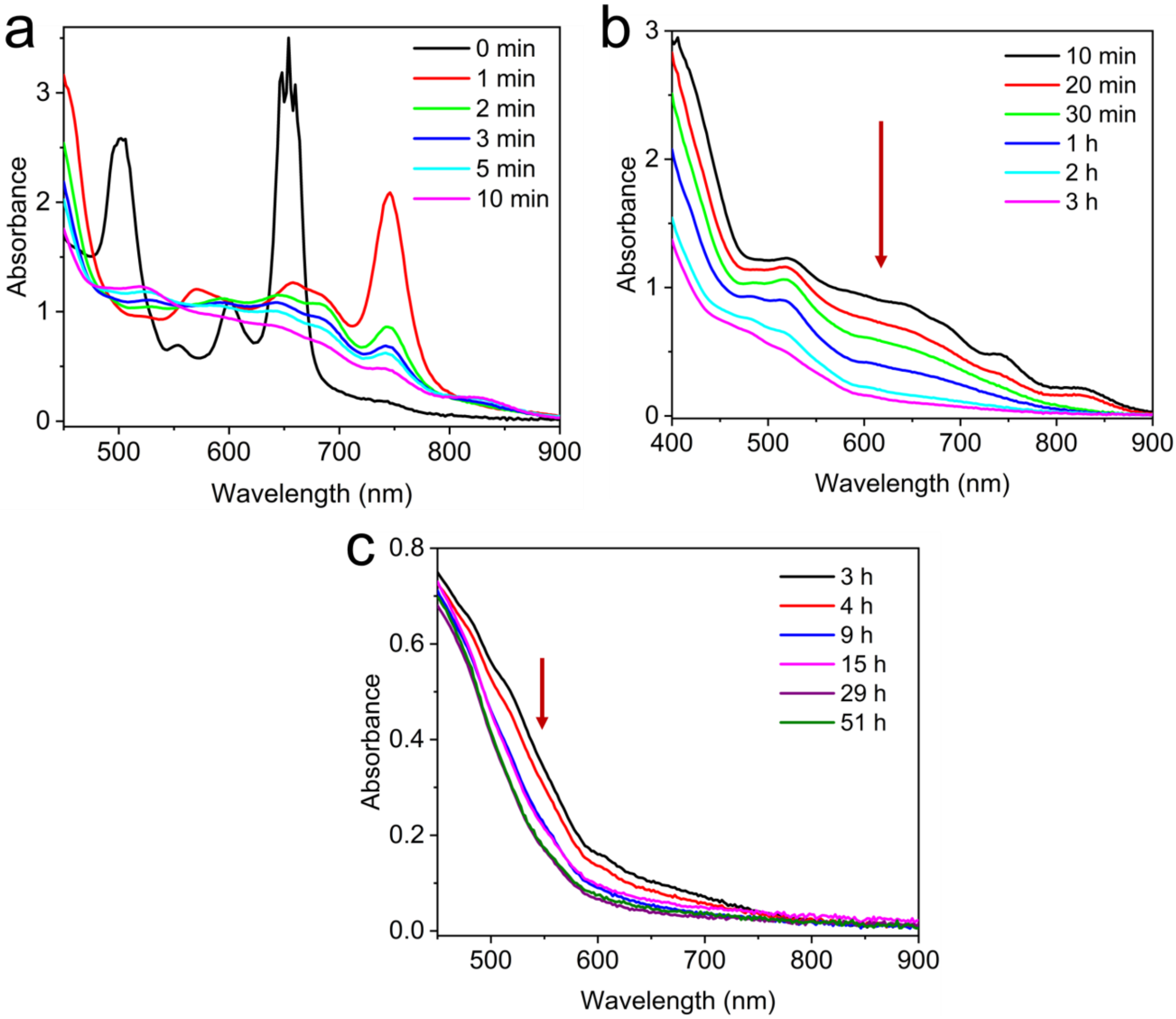

Supplementary Figure 32. UV-vis absorption spectra. UV-vis absorption spectra of CO_2-saturated DMF solution containing 50 μM $F_{20}Ch$, 1.0 μM FeTDHPP, and 50 mM BIH in the same photocatalytic flask (3.05-cm path length) used in our study for CO production. Irradiation time ranging from 0 to 10 min (a), from 10 min to 3 h (b), and from 3 h to 51 h (c). Conditions: red LED light (λ = 630 nm, 110 mW/cm^2) at 293 K.

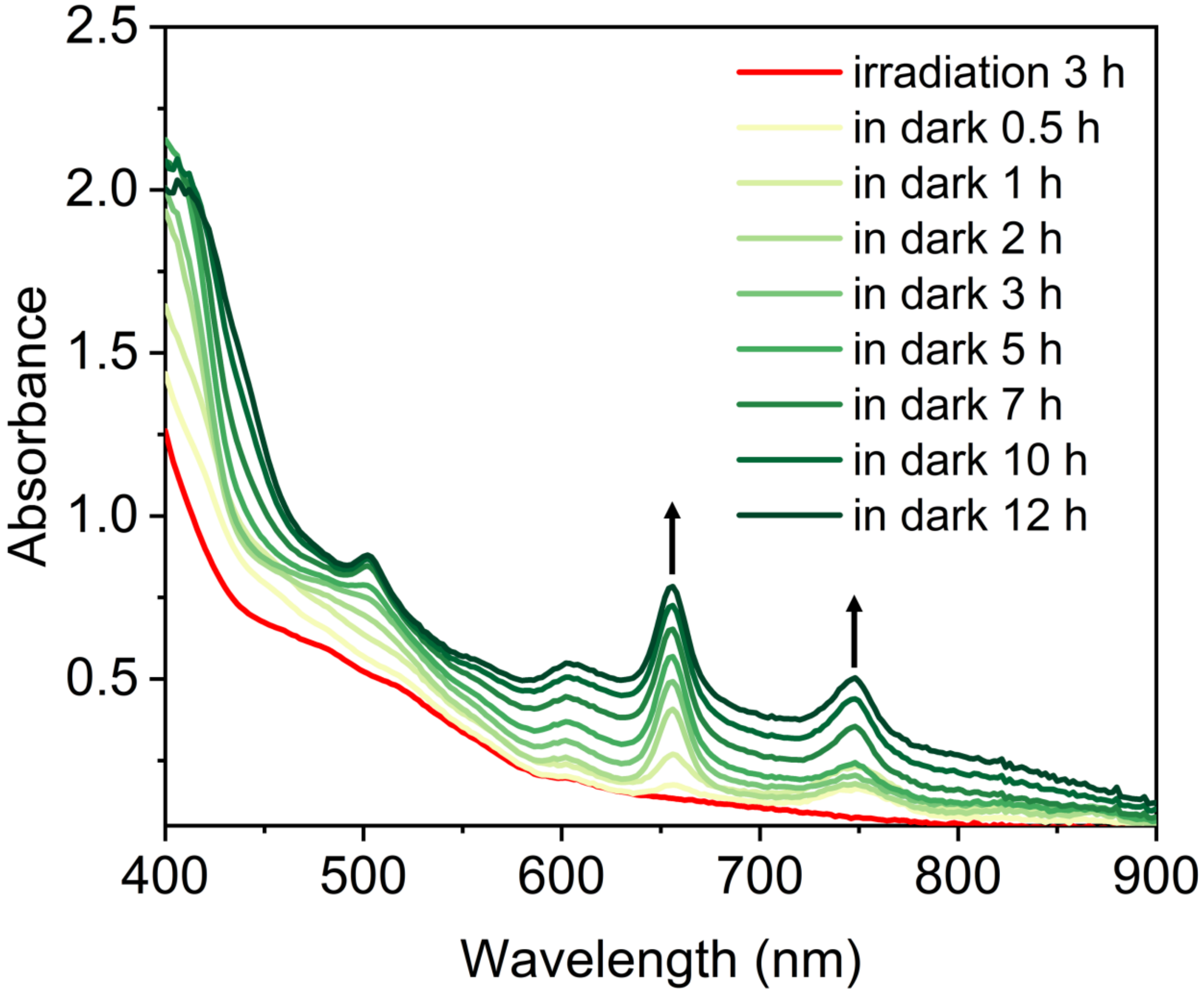

Supplementary Figure 33. UV-vis absorption spectra. UV-vis absorption spectra of a CO_2-saturated DMF solution containing 50 µM $F_{20}Ch$, 1.0 µM FeTDHPP, and 50 mM BIH in a photocatalytic flask (3.05-cm path length) upon irradiation with red LED light (λ = 630 nm, 110 mW/cm^2) for 3 h at 293 K. The mixture was then kept in the dark over 12 h. The additional amount of CO generated at 5 h was determined to be 0.165 ± 0.008 µmol (TON = 0.66 ± 0.03 vs $F_{20}Ch$). The data from 3 separated runs are shown in Supplementary Table 8:

Supplementary Table 8. Amount of CO generated in the dark after 3 h irradiation of a CO_2-saturated DMF solution containing 50 µM $F_{20}Ch$, 1.0 µM FeTDHPP, and 50 mM BIH (under the same conditions described above in supplementary Fig.33). Error bars denote standard deviations, based on at least three separated runs.

Number	CO (after 3 h irradiation) (µmol)	CO (solution kept in dark for 5 h) (µmol)	ΔCO (µmol)	TON_{CO} (vs $F_{20}Ch$)
1	4.019	4.195	0.173	0.69
2	3.067	3.224	0.157	0.63
3	3.397	3.562	0.165	0.66
Average			0.165 ± 0.008	0.66 ± 0.03

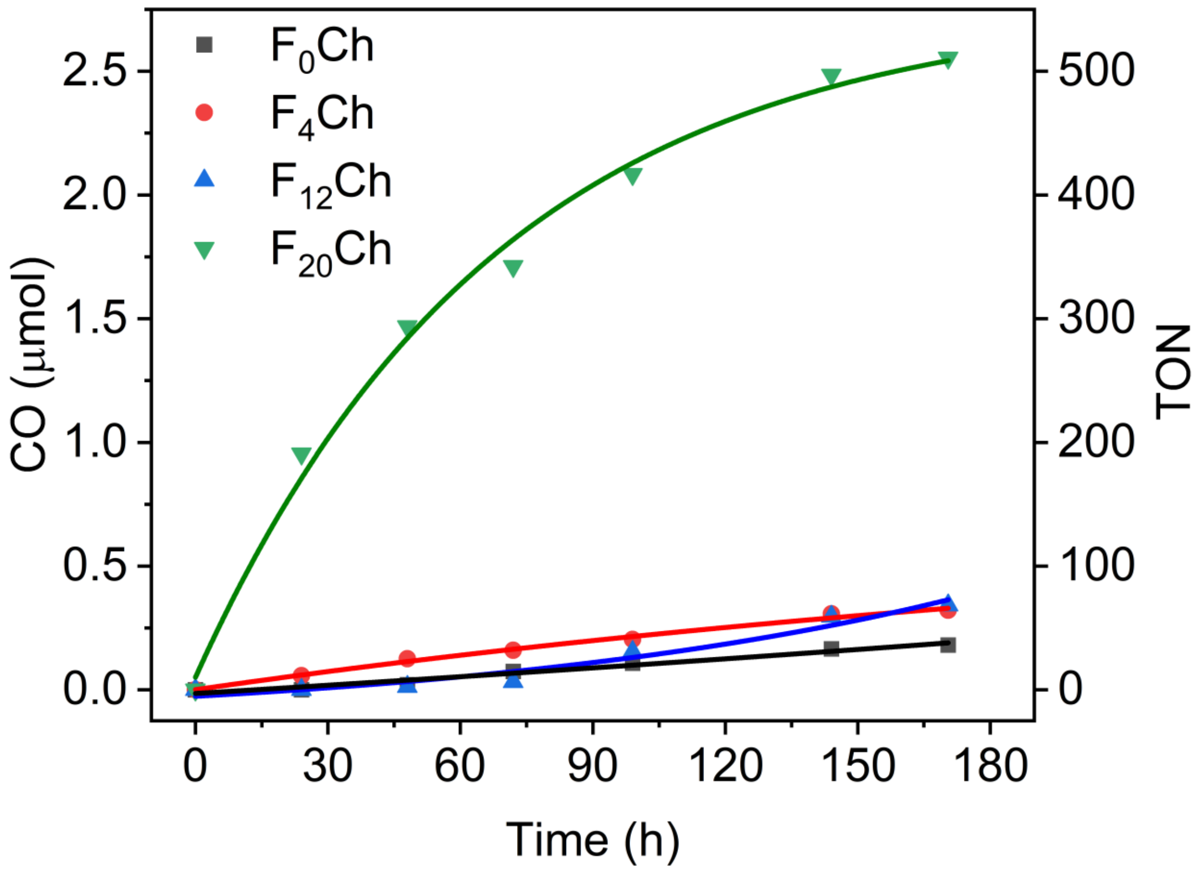

Supplementary Figure 34. Photocatalytic CO_2 reduction. Photocatalytic CO production in CO_2-saturated DMF solutions containing 50 mM BIH, 1.0 μM FeTDHPP, and 50 μM F_0Ch (black), or F_4Ch (red), or $F_{12}Ch$ (blue), or $F_{20}Ch$ (green) under irradiation with 730 nm LED (80 mW/cm^2) at 293 K.

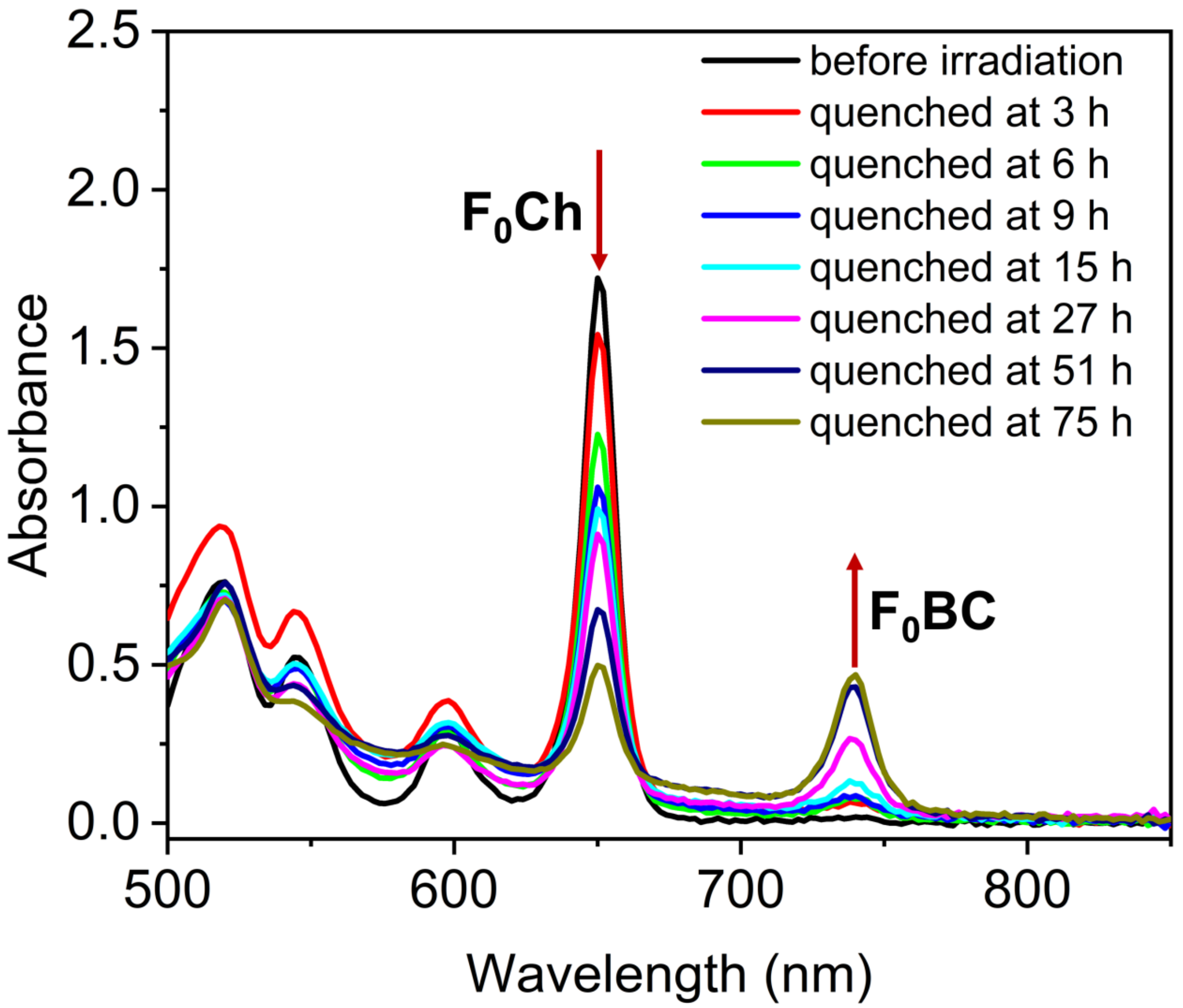

Supplementary Figure 35. UV-vis absorption spectra of photolysis solutions during CO_2RR. The spectra were recorded at different irradiation time for solutions after treatment of the catalytic solution first using excess Co(dmgH)$_2$PyCl and then exposure to the air. The initial system contains 50 µM F_0Ch, 50 mM BIH and 1.0 µM FeTDHPP in CO_2-saturated DMF irradiated with 630 nm LED. 2.52 µmol Co(dmgH)$_2$PyCl in DMF (210 µL) was injected under N_2 and allowed to stir for 3 h in the dark, then exposed to the air for 1 h prior to each UV-vis measurement.

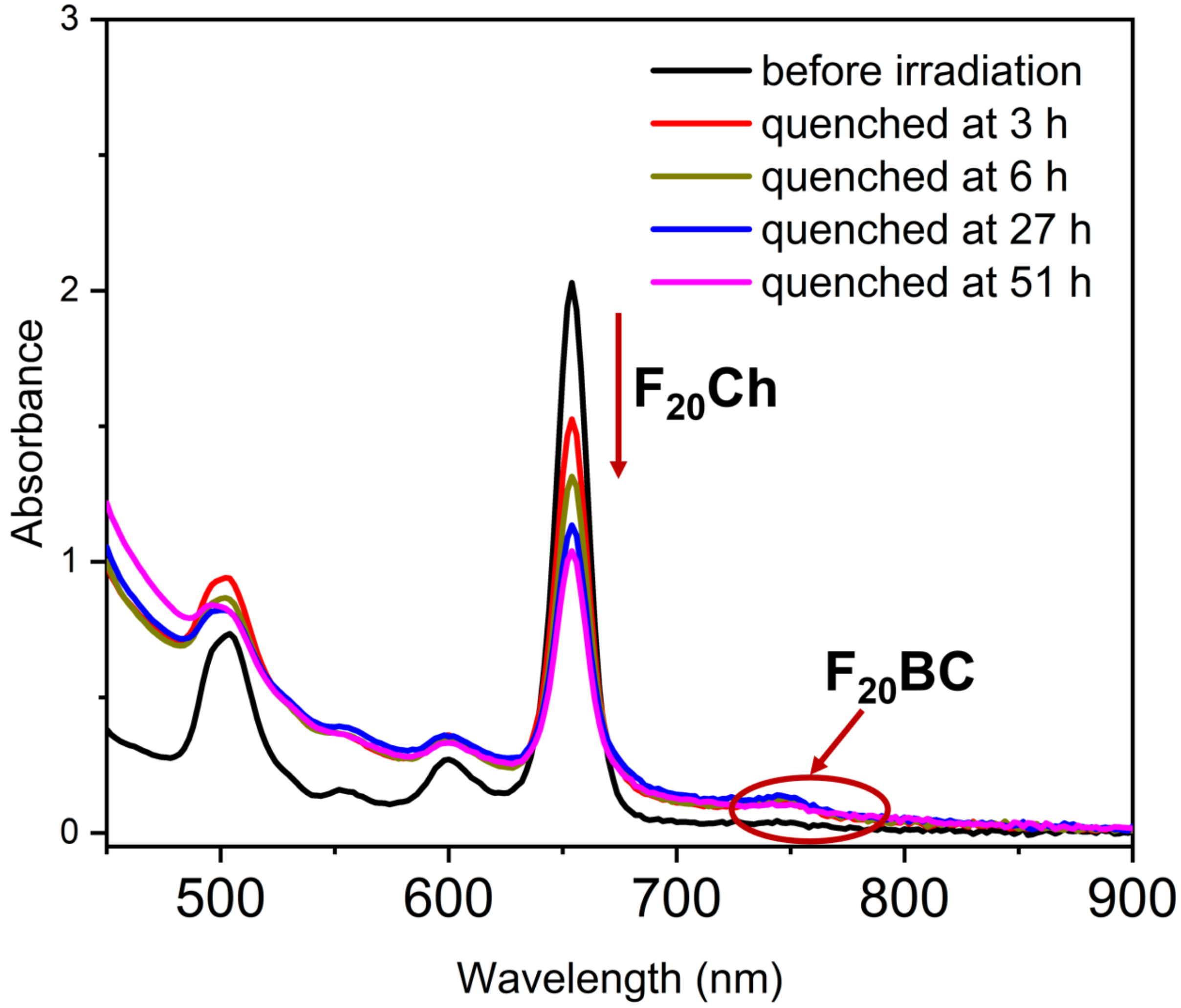

Supplementary Figure 36. UV-vis absorption spectra of photolysis solutions during CO_2RR. The spectra were recorded at different irradiation time for solutions after treatment of the catalytic solution first using excess Co(dmgH)$_2$PyCl and then exposure to the air. The initial system contains 50 μM F_{20}Ch, 50 mM BIH and 1.0 μM FeTDHPP in CO_2-saturated DMF irradiated with 630 nm LED. 2.52 μmol Co(dmgH)$_2$PyCl in DMF (210 μL) was injected under N_2 and allowed to stir for 3 h in the dark, then exposed to the air for 1 h prior to each UV-vis measurement.

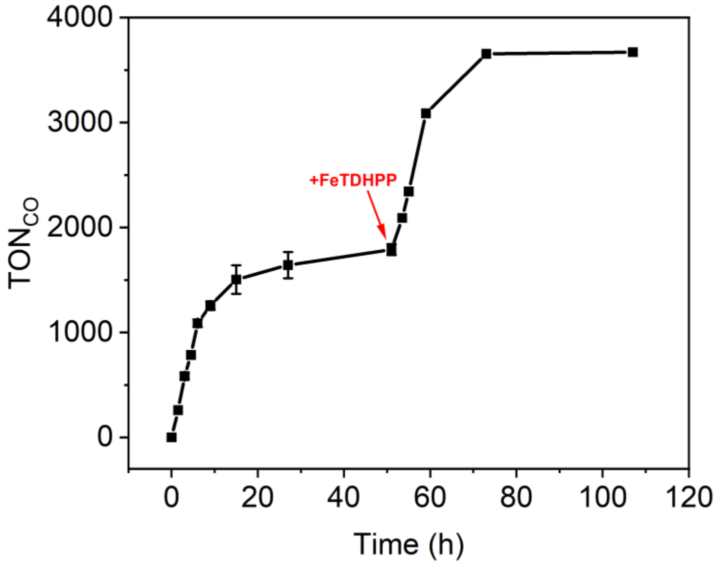

Supplementary Figure 37. Photocatalytic CO_2 reduction. Photocatalytic systems containing 50 μM F_{20}Ch, 50 mM BIH, and 1 μM FeTDHPP under red LED (λ = 630 nm, 110 mW/cm^2) at 293 K. The same amount of FeTDHPP in DMF was added to the system under CO_2 at 51 h. Error bars denote standard deviations, based on at least three separated runs.

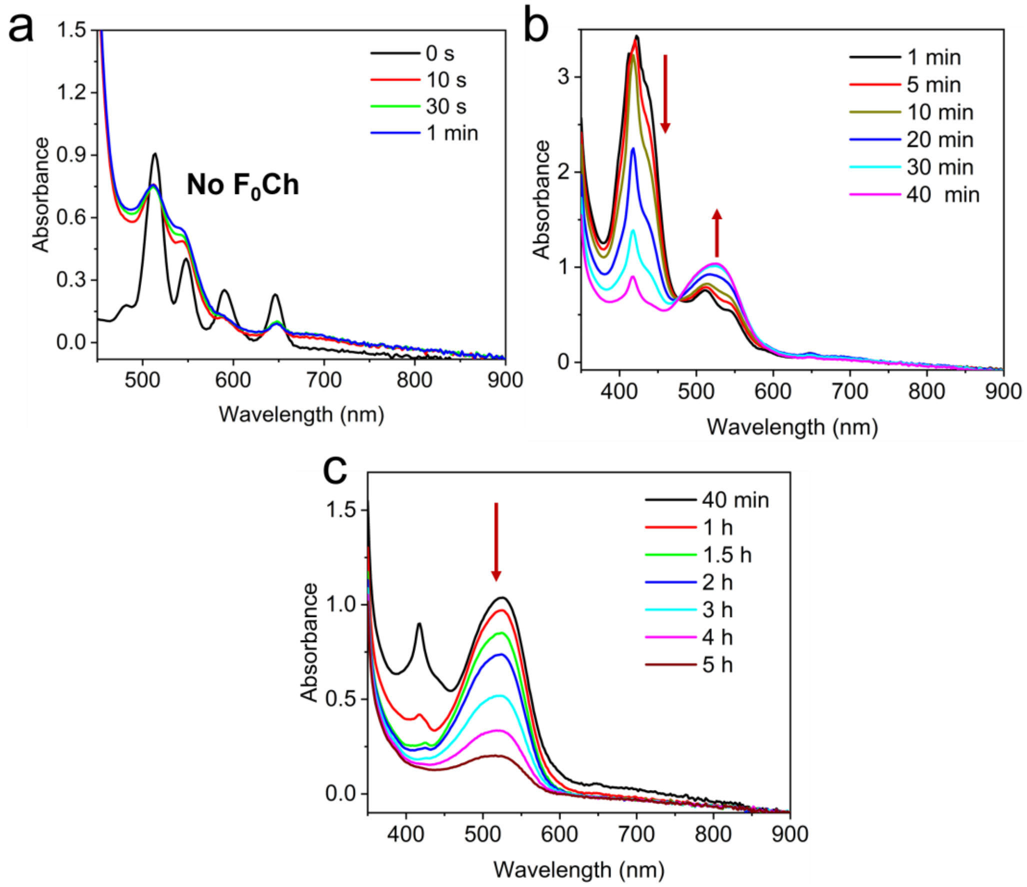

Supplementary Figure 38. UV-vis absorption spectra. UV-vis absorption spectra of systems containing 50 μM F_0TPP, 1 μM FeTDHPP, and 20 mM BIH in DMF under CO_2 upon irradiation with red LED light (λ = 630 nm, 110 mW/cm^2) in a quartz cuvette (10-mm path length). Irradiation time ranging from 0 to 1 min (a), from 1 min to 40 min (b), and from 40 min to 5 h (c). Note: no F_0Ch was observed.

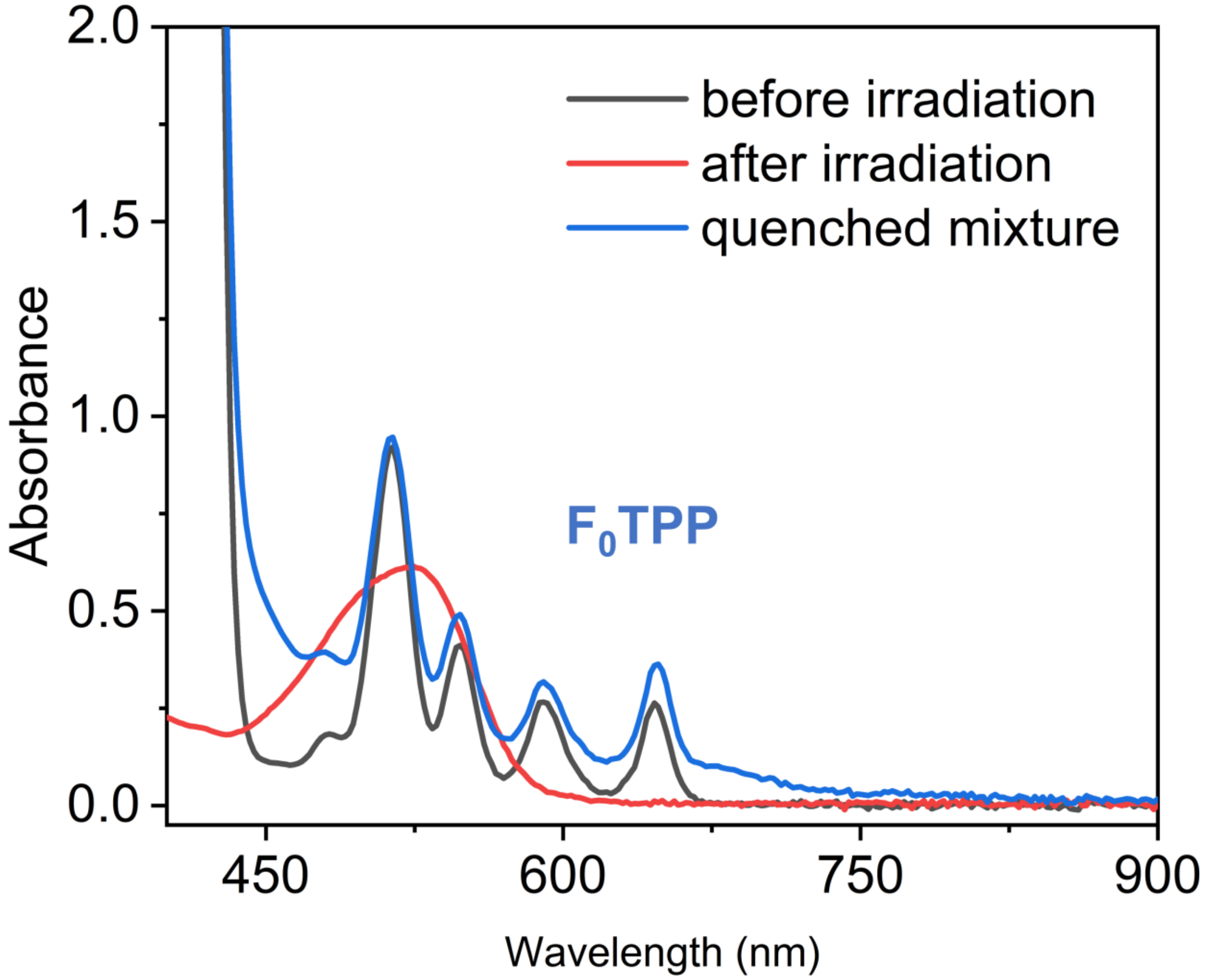

Supplementary Figure 39. UV-vis absorption spectra of photolysis solutions during CO_2RR. The spectra were recorded for solutions before and after a 4 h irradiation, and after treatment of the catalytic solution first using excess Co(dmgH)$_2$PyCl and then exposure to the air. The initial system contains 50 µM F_0TPP, 50 mM BIH and 1.0 µM FeTDHPP in CO_2-saturated DMF irradiated with 630 nm LED. 2.52 µmol Co(dmgH)$_2$PyCl in DMF (210 µL) was injected at 4 h under N_2 and allowed to stir for 3 h in the dark, then exposed to the air for 1 h prior to UV-vis measurement

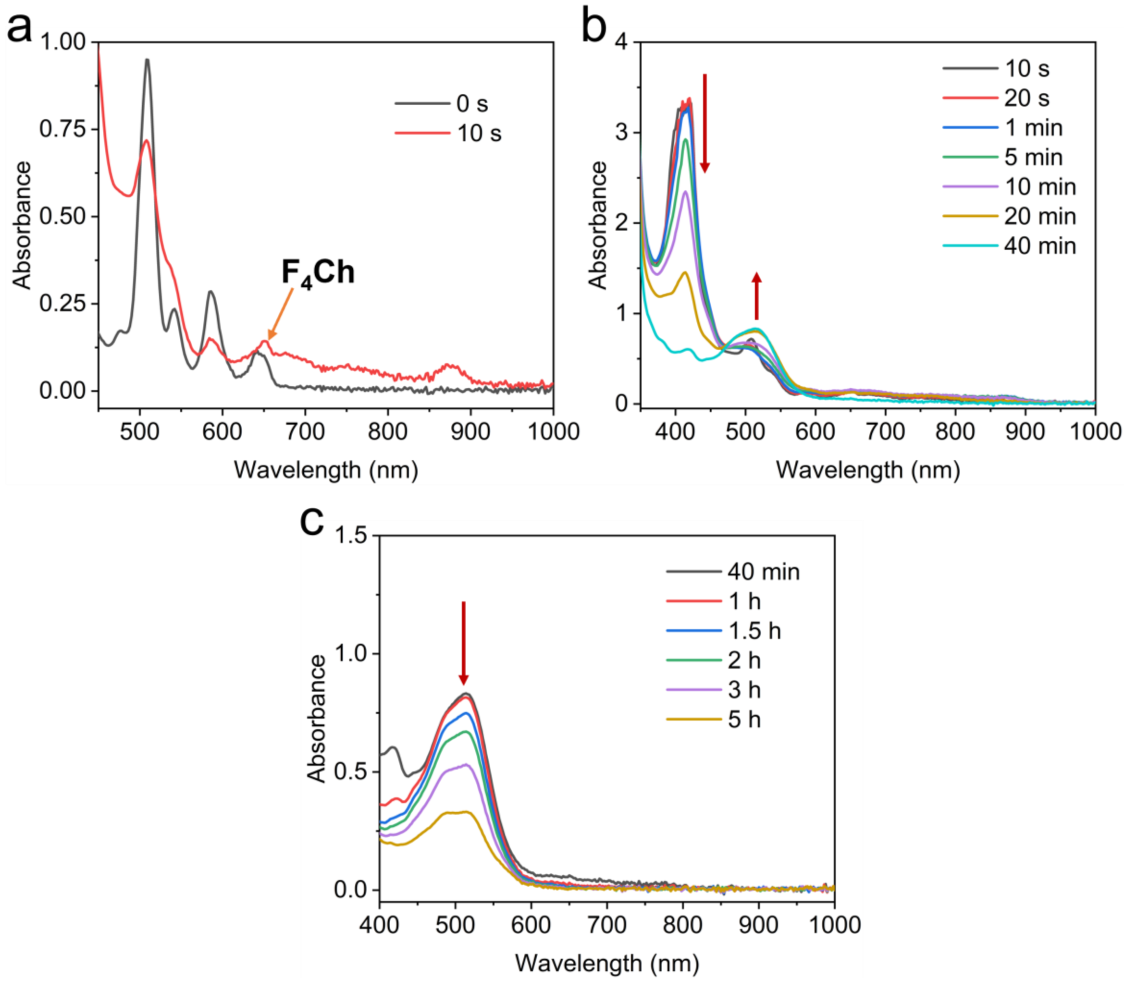

Supplementary Figure 40. UV-vis absorption spectra. UV-vis absorption spectra of systems containing 50 μM F_4TPP, 1.0 μM FeTDHPP, and 20 mM BIH in DMF under CO_2 upon irradiation with red LED light (λ = 630 nm, 110 mW/cm^2) in a quartz cuvette (10-mm path length). Irradiation time ranging from 0 to 10 s (a), from 10 s to 40 min (b), and from 40 min to 5 h (c).

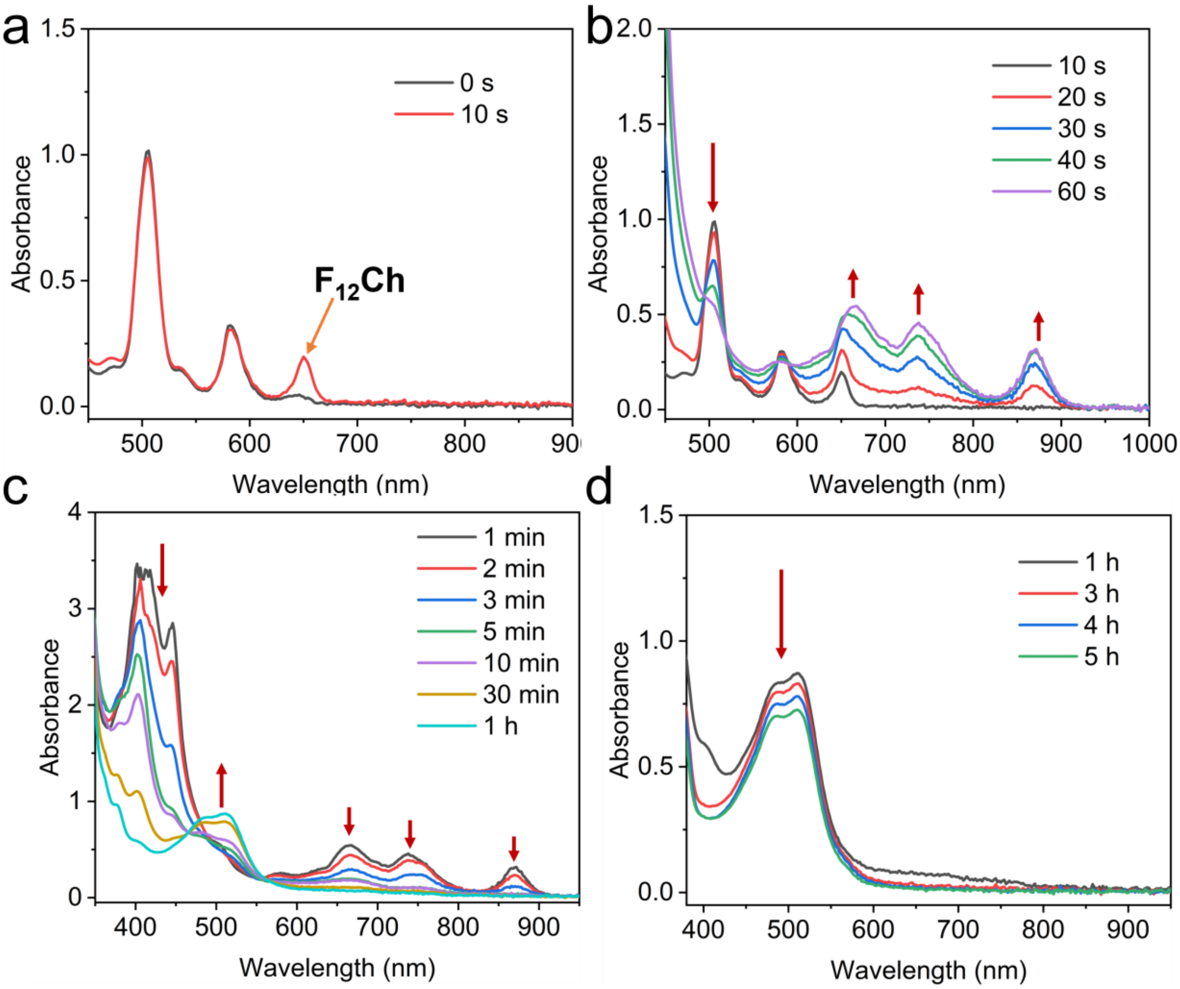

Supplementary Figure 41. UV-vis absorption spectra. UV-vis absorption spectra of systems containing 50 µM F_{12}TPP, 1.0 µM FeTDHPP, and 20 mM BIH in DMF under CO_2 upon irradiation with red LED light (λ = 630 nm, 110 mW/cm^2) in a quartz cuvette (10-mm path length). Irradiation time ranging from 0 to 10 s (a), from 10 s to 60 s (b), from 1 min to 1 h (c), and from 1 h to 5 h (d).

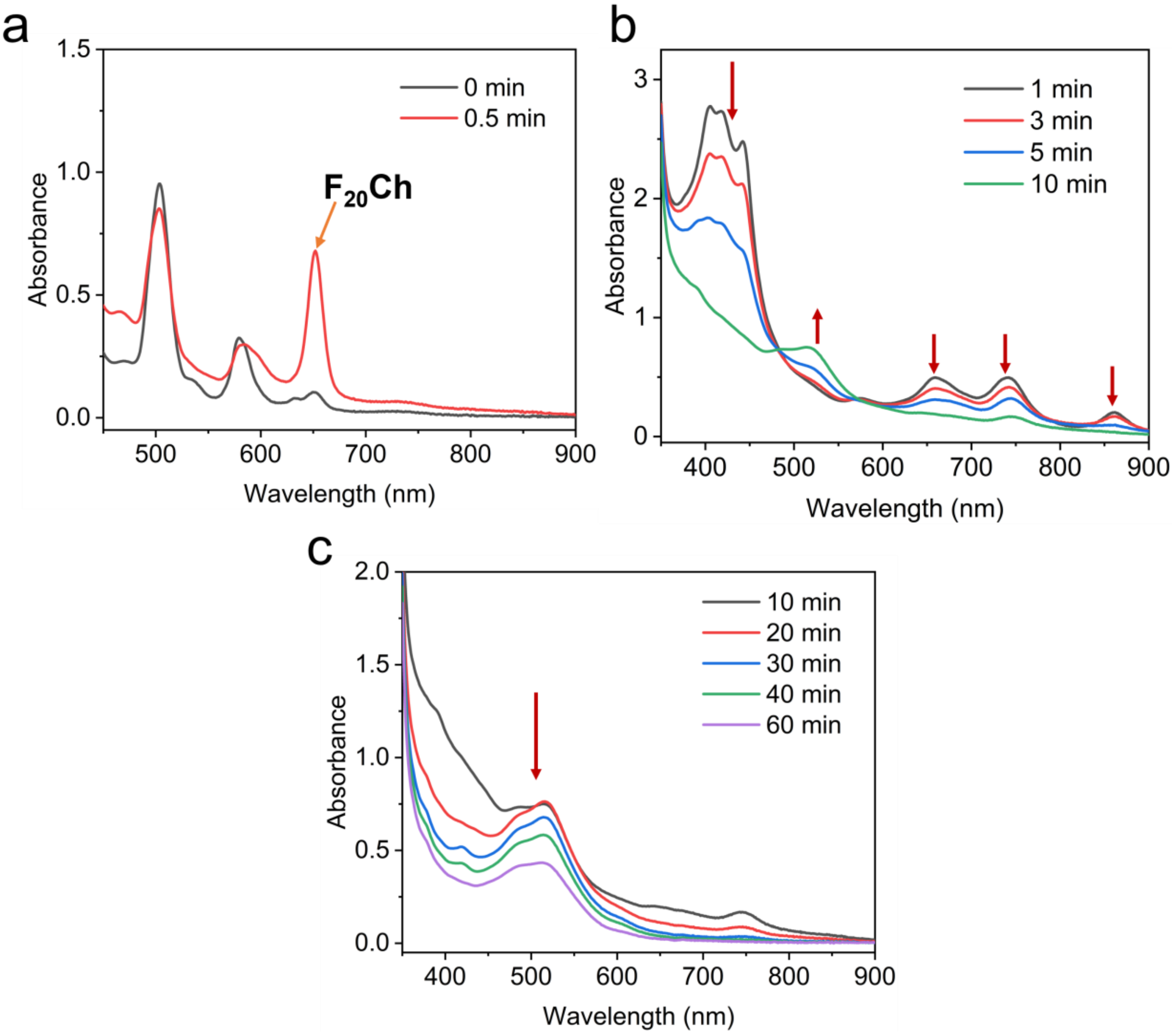

Supplementary Figure 42. UV-vis absorption spectra. UV-vis absorption spectra of systems containing 50 μM F_{20}TPP, 1.0 μM FeTDHPP, and 20 mM BIH in DMF under CO_2 upon irradiation with red LED light (λ = 630 nm, 110 mW/cm^2) in a quartz cuvette (10-mm path length). Irradiation time ranging from 0 to 0.5 min (a), from 1 min to 10 min (b), and from 10 min to 60 min (c).

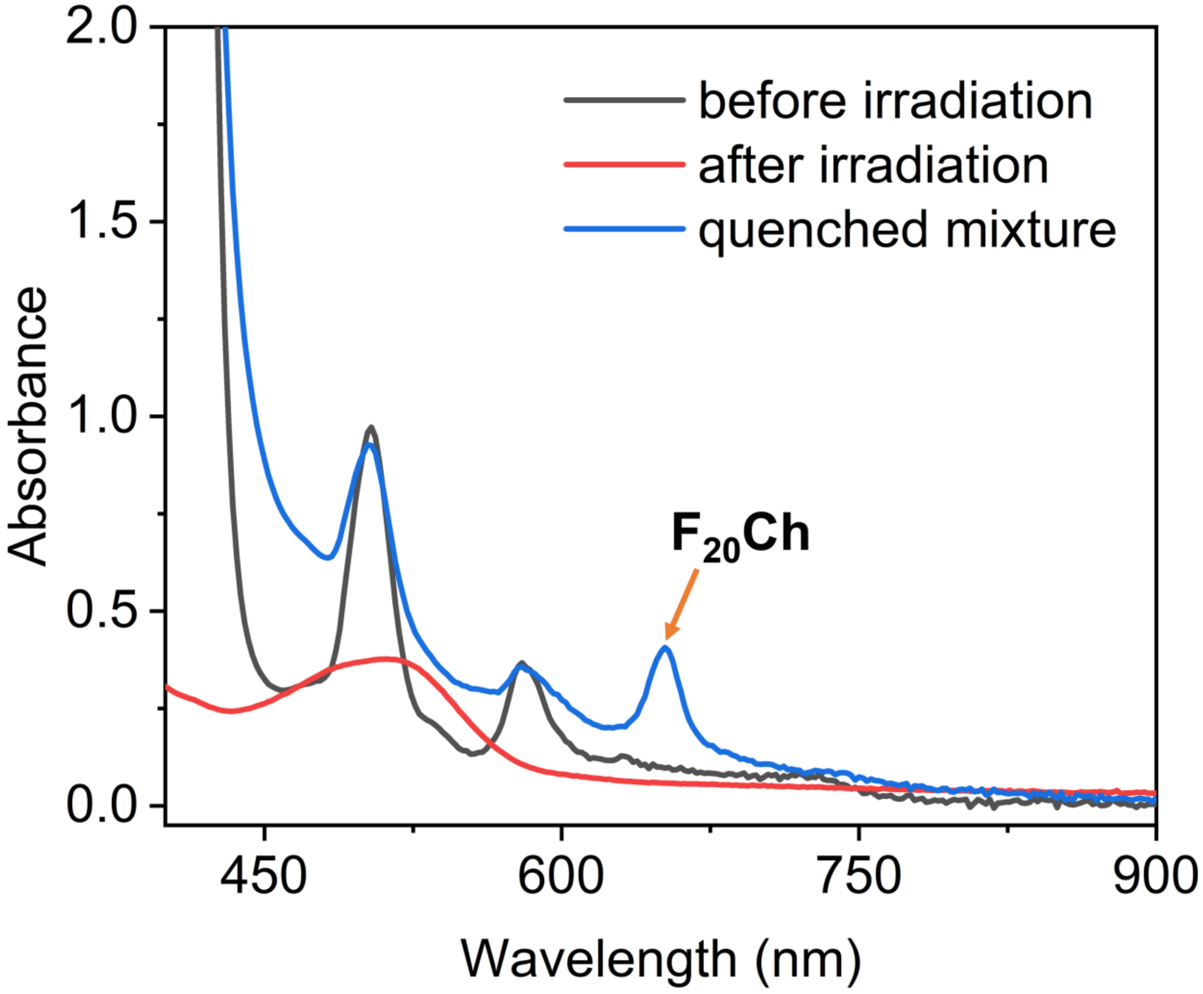

Supplementary Figure 43. UV-vis absorption spectra of photolysis solutions during CO_2RR. The spectra were recorded for solutions before and after a 4 h irradiation, and after treatment of the catalytic solution first using excess Co(dmgH)$_2$PyCl and then exposure to the air. The initial system contains 50 µM F_{20}TPP, 50 mM BIH and 1.0 µM FeTDHPP in CO_2-saturated DMF irradiated with 630 nm LED. 2.52 µmol Co(dmgH)$_2$PyCl in DMF (210 µL) was injected at 4 h under N_2 and allowed to stir for 3 h in the dark, then exposed to the air for 1 h prior to UV-vis measurement.

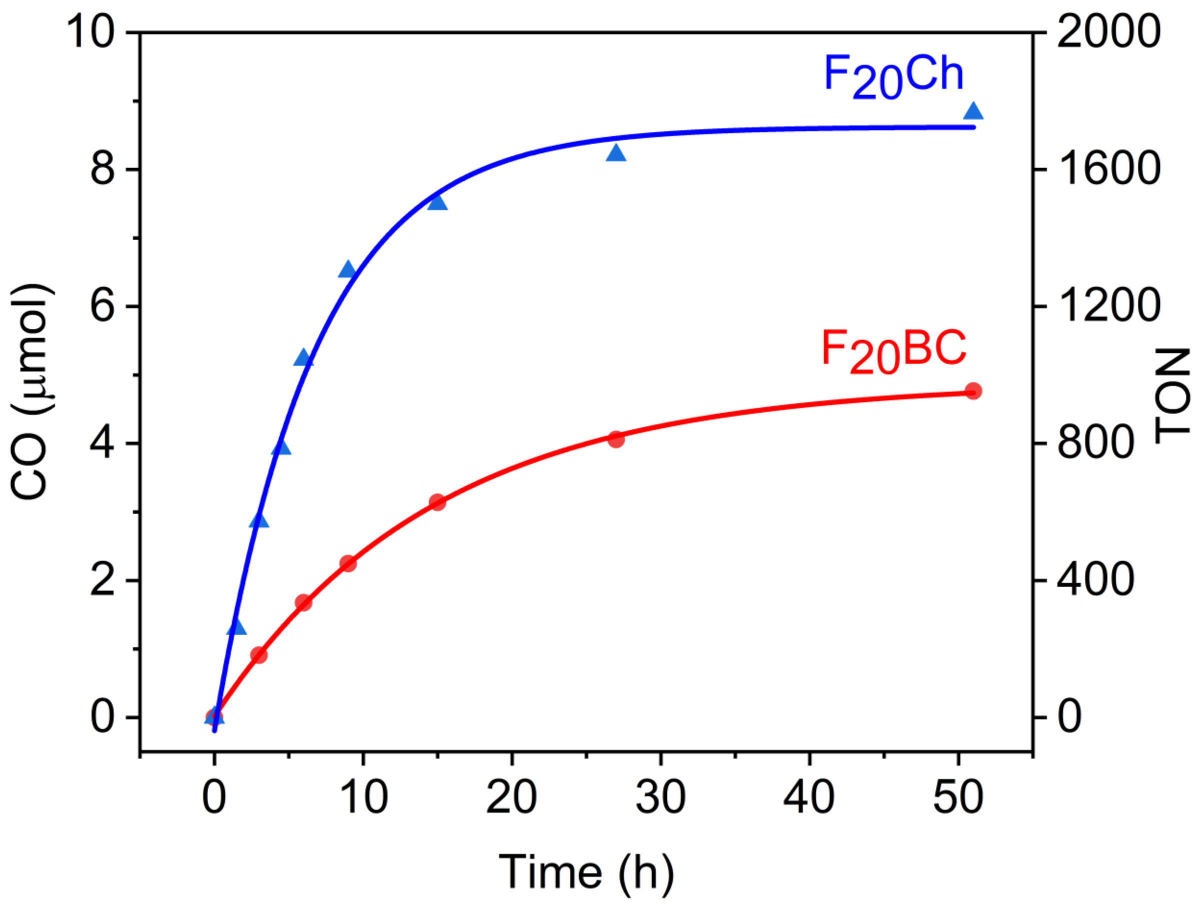

Supplementary Figure 44. Photocatalytic CO_2 reduction. Photocatalytic CO production in CO_2-saturated DMF solutions containing 50 mM BIH, 1.0 μM FeTDHPP, and 50 μM F_{20}Ch (blue) or F_{20}BC (red), under irradiation with red LEDs (λ = 630 nm, 110 mW/cm^2) at 293 K.

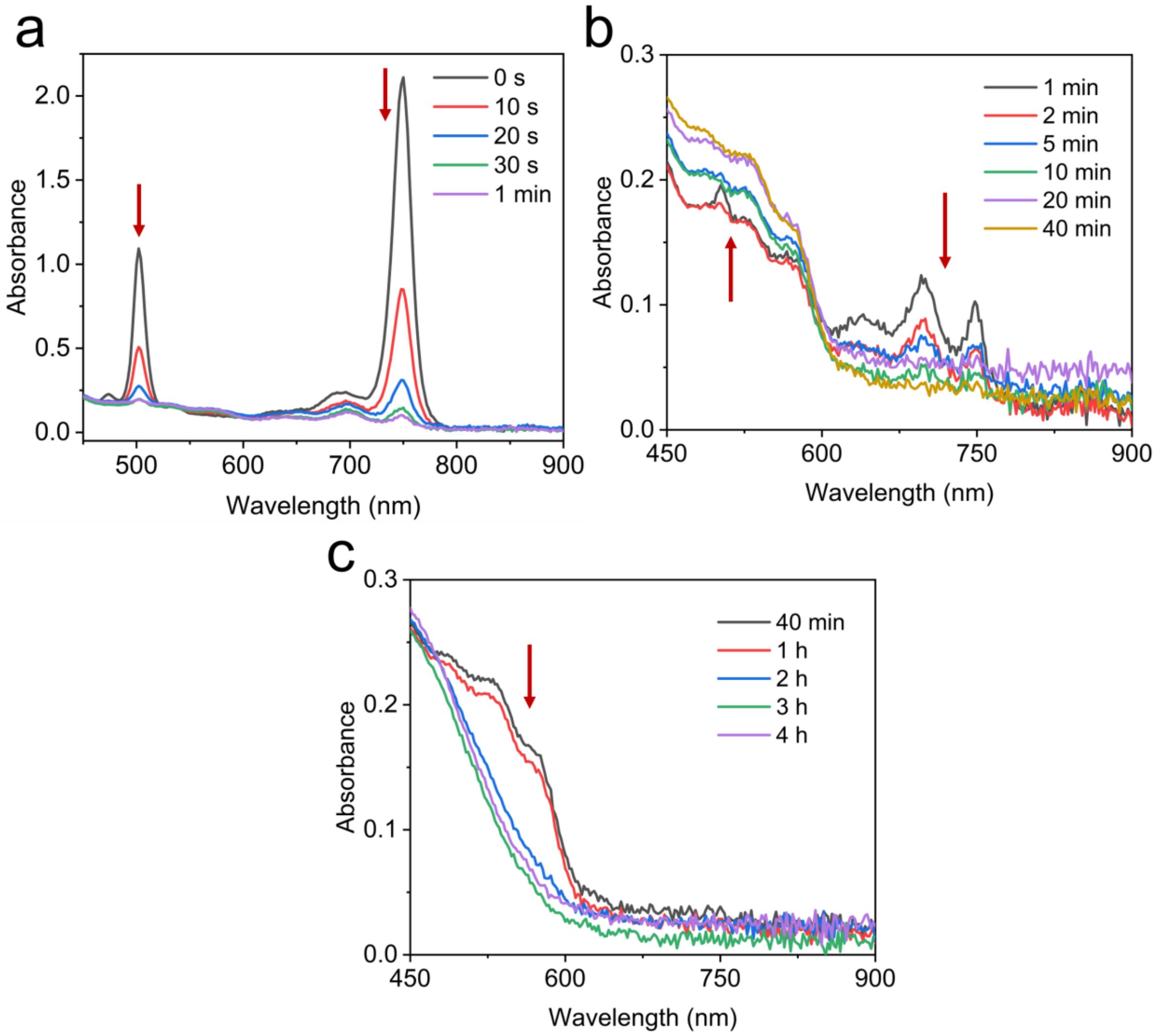

Supplementary Figure 45. UV-vis absorption spectra. UV-vis absorption spectra of CO_2-saturated DMF solution containing 10 µM $F_{20}BC$, 1.0 µM FeTDHPP, and 20 mM BIH in the same photocatalytic flask (3.05-cm path length) used in our study for CO production. Irradiation time ranging from 0 to 1 min (a), from 1 min to 40 min (b), and from 40 min to 4 h (c). Conditions: red LED light (λ = 630 nm, 110 mW/cm^2) at 293 K.

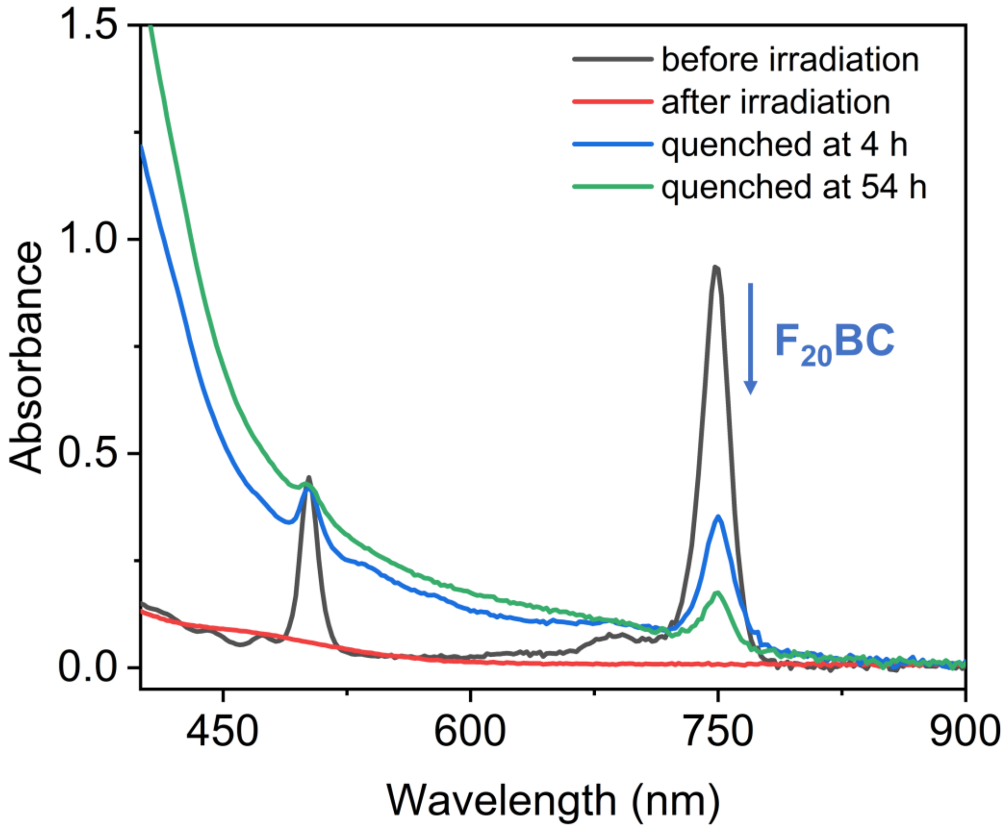

Supplementary Figure 46. UV-vis absorption spectra of photolysis solutions during CO_2RR. The spectra were recorded for solutions before and after irradiation, and after treatment of the catalytic solution first using excess Co(dmgH)$_2$PyCl and then exposure to the air. The initial system contains 10 μM F_{20}BC, 50 mM BIH and 1.0 μM FeTDHPP in CO_2-saturated DMF irradiated with 730 nm LED. 2.52 μmol Co(dmgH)$_2$PyCl in DMF (210 μL) was injected at 4 h or 54 h under N_2 and allowed to stir for 3 h in the dark, then exposed to the air for 1 h prior to UV-vis measurement.

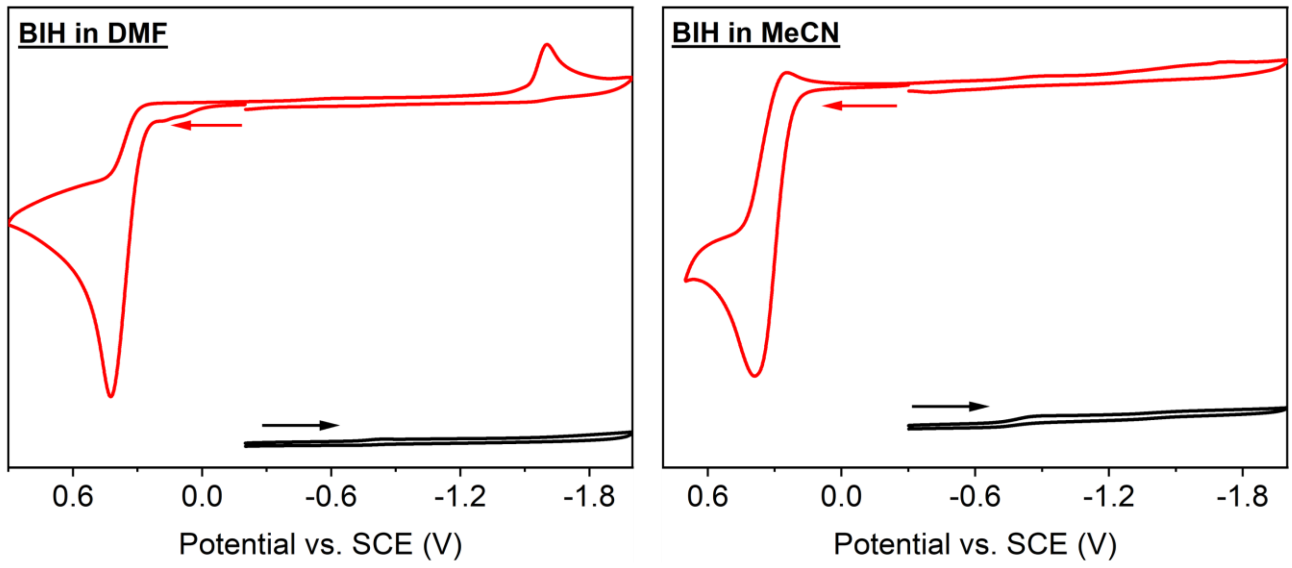

Supplementary Figure 47. Electrochemical study. Cyclic voltammograms of 5 mM BIH in DMF (left) or MeCN (right) containing 0.1 M $TBAPF_6$ under Ar at a scan rate of 0.1 $V \cdot s^{-1}$.

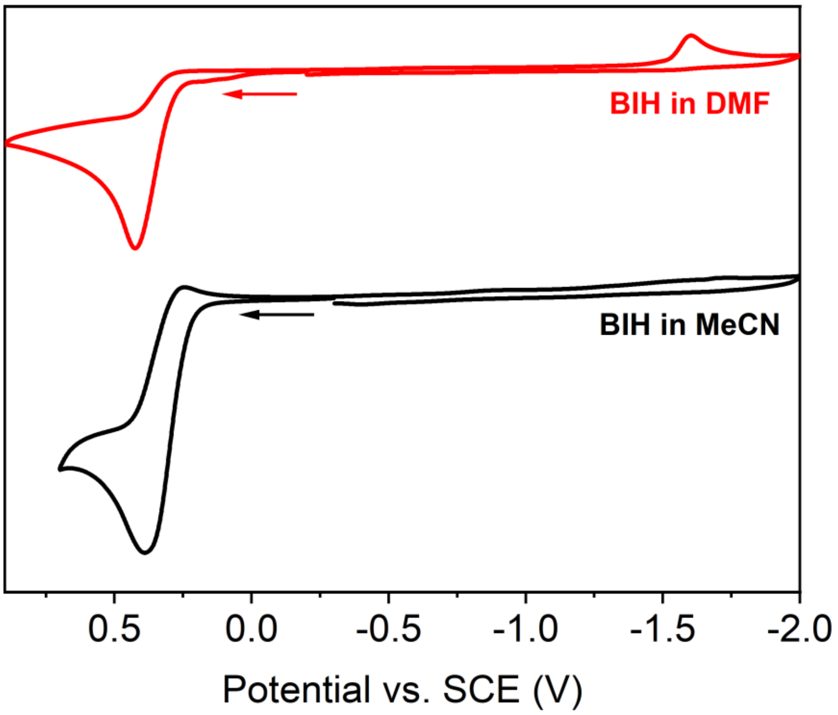

Supplementary Figure 48. Electrochemical study. Cyclic voltammograms of 5 mM BIH in MeCN (bottom) or DMF (top) containing 0.1 M $TBAPF_6$ under Ar at a scan rate of 0.1 $V\cdot s^{-1}$

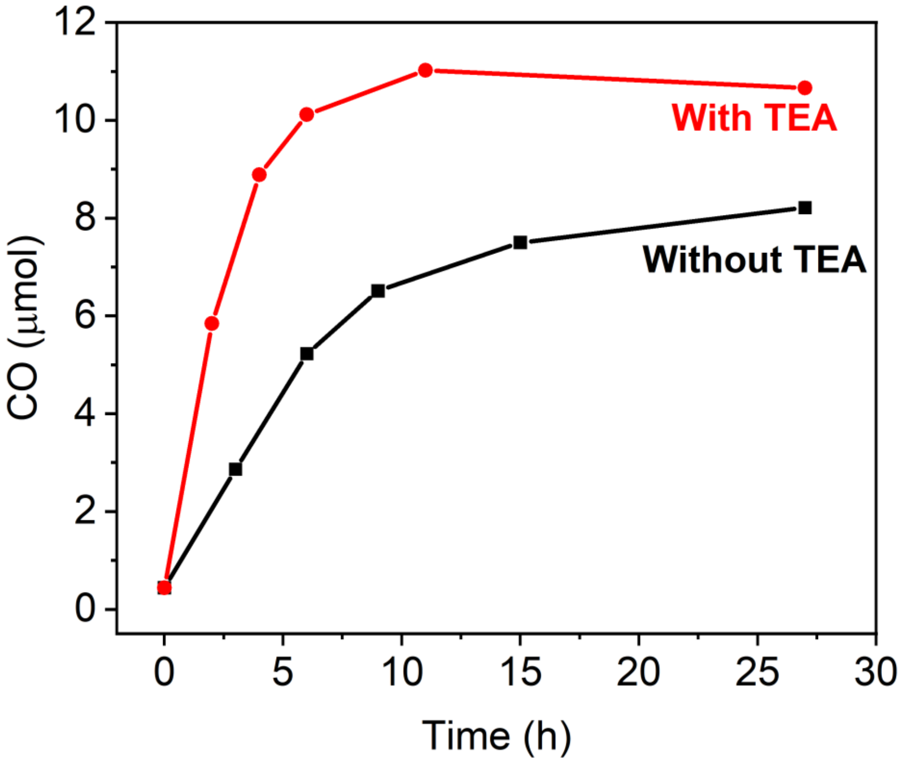

Supplementary Figure 49. Photocatalytic CO_2 reduction. Time profiles of photocatalytic CO_2 reduction in CO_2-saturated DMF (black) or DMF/TEA = 4 (red) solutions containing 50 µM F_{20}Ch, 1 µM FeTDHPP and 50 mM BIH under red LED (λ = 630 nm, 110 mW/cm^2) at 293 K.

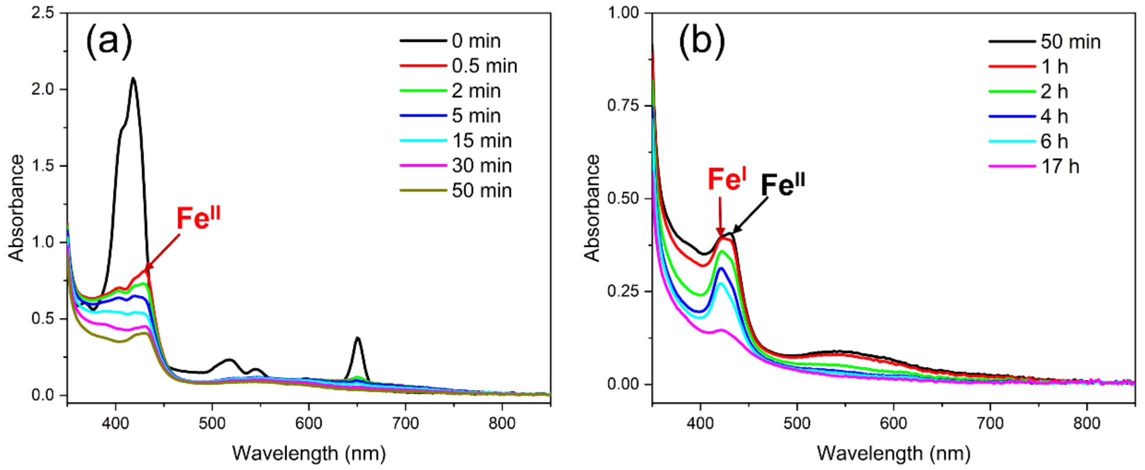

Supplementary Figure 50. UV-vis absorption spectra. Systems containing 10 μM F_0Ch, 10 μM FeTDHPP and 10 mM BIH in DMF under CO_2 upon irradiation with red LED light (λ = 630 nm, 110 mW/cm^2) in a quartz cuvette (10-mm path length). Irradiation time ranging from 0 to 50 min (a), and from 50 min to 17 h (b).

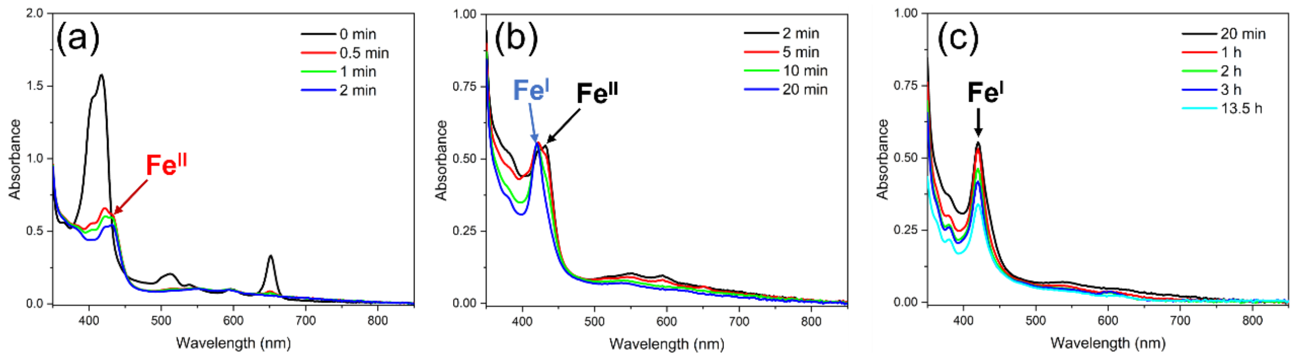

Supplementary Figure 51. UV-vis absorption spectra. Systems containing 10 μM F_4Ch, 10 μM FeTDHPP and 10 mM BIH in DMF under CO_2 upon irradiation with red LED light (λ = 630 nm, 110 mW/cm^2) in a quartz cuvette (10-mm path length). Irradiation time ranging from 0 to 2 min (a), from 2 min to 20 min (b), and from 20 min to 13.5 h (c).

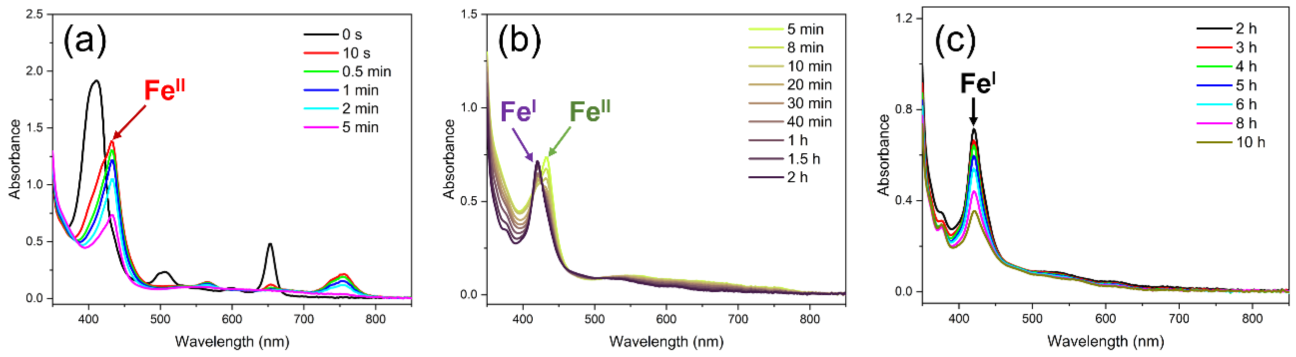

Supplementary Figure 52. UV-vis absorption spectra. Systems containing 10 μM F_{12}Ch, 10 μM FeTDHPP and 10 mM BIH in DMF under CO_2 upon irradiation with red LED light (λ = 630 nm, 110 mW/cm^2) in a quartz cuvette (10-mm path length). Irradiation time ranging from 0 to 5 min (a), from 5 min to 2 h (b), and from 2 h to 10 h (c).

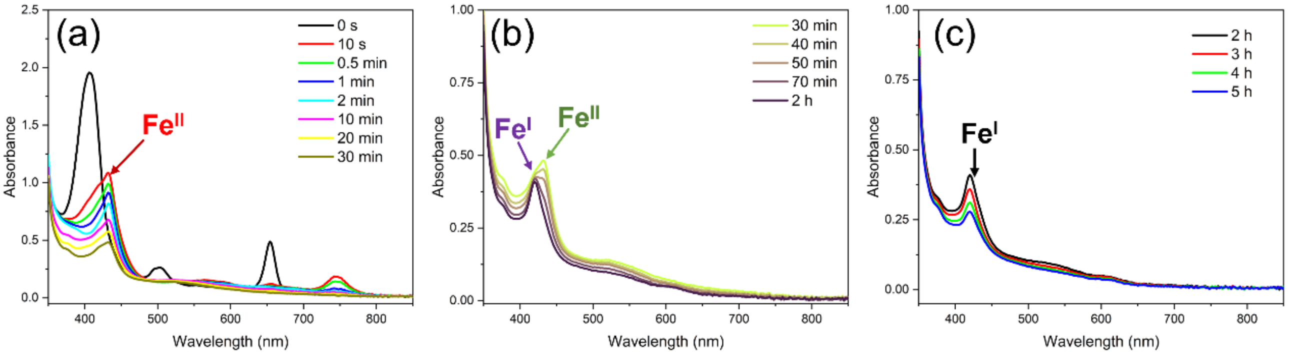

Supplementary Figure 53. UV-vis absorption spectra. Systems containing 10 μM F_{20}Ch, 10 μM FeTDHPP and 10 mM BIH in DMF under CO_2 upon irradiation with red LED light (λ = 630 nm, 110 mW/cm^2) in a quartz cuvette (10-mm path length). Irradiation time ranging from 0 to 30 min (a), from 30 min to 2 h (b), and from 2 h to 5 h (c).

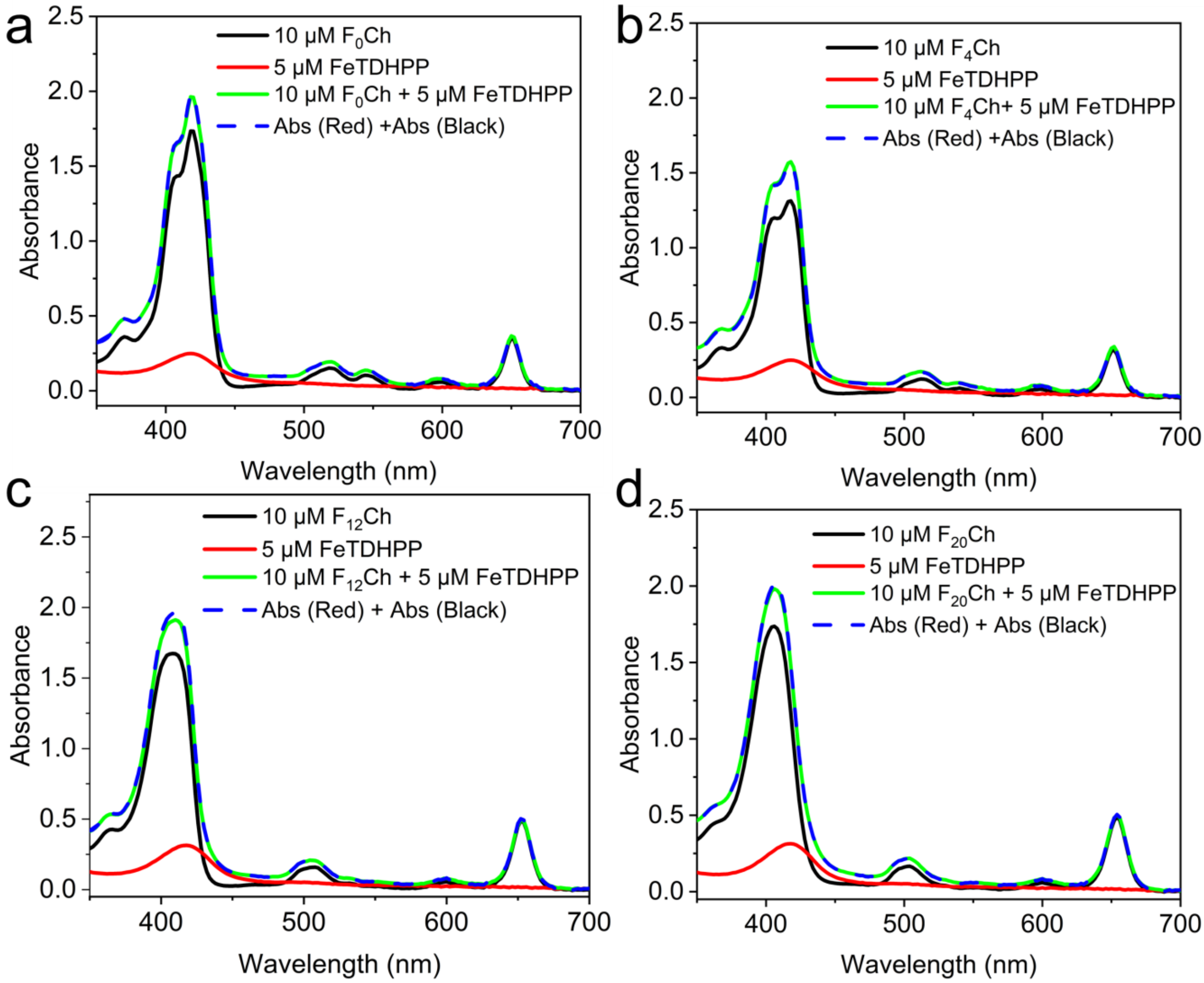

Supplementary Figure 54. UV-vis absorption spectra. Systems containing 10 μM chromophores (black), 5 μM FeTDHPP (red), a mixture of 10 μM chromophores and 5 μM FeTDHPP (green), and sum of the spectra of chromophore and FeTDHPP (blue). chromophores: F_0Ch (a); F_4Ch (b); F_{12}Ch (c); F_{20}Ch (d).

Supplementary Table 9. Crystal data and structure refinement for F_{20}BC.

CCDC	2289797
Empirical formula	$C_{50}H_{28}F_{20}N_4O$
Formula weight	1080.76
Temperature/K	149.99(10)
Crystal system	monoclinic
Space group	$P2_1/c$ (14)
a/Å	12.6102(3)
b/Å	11.7815(3)
c/Å	15.0690(4)
α/°	90
β/°	102.937(3)
γ/°	90
Volume/Å^3	2181.93(10)
Z	2
ρ_{calc}g/cm^3	1.645
μ/mm^{-1}	1.418
F(000)	1088
Crystal size/mm^3	0.15×0.12×0.1
Radiation	CuK_α (λ = 1.54178 Å)
2θ range for data collection/°	7.19 to 145.59 (0.81 Å)
Index ranges	$-15 \le h \le 14$, $-14 \le k \le 13$, $-11 \le l \le 18$
Reflections collected	8720
Independent reflections	4231 [R_{int} = 0.0396, R_{sigma} = 0.0404]
Data/restraints/parameters	4231/30/357
Goodness-of-fit on F^2	1.035
Final R indexes [I >= 2σ (I)]	R_1 = 0.0557, wR_2 = 0.1546
Final R indexes [all data]	R_1 = 0.0620, wR_2 = 0.1631
Largest peak/hole / e Å^{-3}	0.71/-0.33

Supplementary Table 10. Comparison of bond lengths. The C-C and C=C bond lengths in the pyrrole ring for **$F_{20}BC$** and similar structures in the literature. The C_7-C_8 bond distance of 1.504(3) Å in **$F_{20}BC$** is characteristic of a single bond, which is consistent with previous reported chlorins[13] and bacteriochlorins[14].

**CSD Refcodes of reported Chlorins**	**Distance of C-C**	**Distance of C=C**	**Distance of C=C**	**Distance of C=C**
JITMIL	1.521(9)	1.356(8)	1.368(9)	1.360(9)
GELGUZ	1.525	1.368	1.348	1.361
GELJEM	1.519	1.359	1.363	1.357
GELQAP	1.548	1.366	1.350	1.345
SAZROC	1.545(2)	1.366(2)	1.346(2)	1.364(2)
BAXWOQ	1.5380(16)	1.3651(18)	1.3508(16)	1.36029(17)
VIQWAU	1.539(2)	1.353(2)	1.352(3)	1.339(3)
**CSD Refcodes of reported bacteriochlorins**	**Distance of C-C**	**Distance of C-C**	**Distance of C=C**	**Distance of C=C**
$F_{20}BC$ (CCDC: 2289797)	1.504(3)	1.504(3)	1.387(3)	1.387(3)
FUBJIV	1.536(4)	1.497(5)	1.366(5)	1.364(4)
SAZRES	1.5365(18)	1.5365(18)	1.3663(18)	1.3663(18)
SAZRIW	1.540(7)	1.532(7)	1.377(7)	1.360(7)
SAZRUI	1.542(3)	1.542(3)	1.373(3)	1.373(3)

Supplementary Table 11. Molecular systems for CO_2 reduction with TEA additive.

System	Major product	Solvent	Sacrificial donor	TON (time)	TOF (h^{-1})	Light source (nm)	Ref
F_{20}Ch(50 μM) + FeTDHPP (1 μM)	CO	20% TEA/DMF	BIH (50 mM)	2132 (27 h)	584	630	This work
[TATA]PF_6 (0.2 mM) + $[Co(qpy)(OH_2)_2]^{2+}$ (2 μM)	CO<br>HCOOH	0.15 M TEA/MeCN	BIH (100 mM)	182 (8 h)<br>419 (8 h)	-	450	15
$[Ru(phen)_3]^{2+}$ (0.2 mM) + $[Co_2biqpy]^{4+}$ (50 μM)	HCOOH	20% TEA/MeCN	BIH (25 mM)	386 (23 h)	-	460	16
Re(PyNHC-PhCF_3)$(CO)_3$Br (100 uM) +*fac*-Ir$(ppy)_3$ (100 uM)	CO	5% TEA/MeCN	BIH (10 mM)	51 (4 h)	12.8	a solar simulator (AM1.5 filter)	17
CuBCP (500 uM) + CoFPC (0.5 μM)	CO	CH_3CN/TEA = 5	BIH (20 mM)	9185 (-)	-	425	18
IrQPY (100 uM) + CoTPA (100 uM)	CO	2.5% TEA /MeCN	BIH (80 mM)	391 ± 7 (4 h)	-	450 ± 5	19
CuPYBCP (100 uM)) + CoPYN5 (100 uM))	CO	1 M TEA/MeCN	BIH (20 mM)	338.0 ± 43.5 (2 h)	263.7 ± 16.1	425	20
Ir$(ppy)_3$ (100 uM) + Au-NHC (**2**^{Cl}) (0.1 uM)	CO	5% TEA/MeCN	BIH (20 mM)	270 (2 h)	28.2	380-750	21
CuPPBCP (500 uM) + CoTCPc (0.5 uM)	CO	CH_3CN/TEA = 5	BIH (50 mM)	11800 ± 1400 (4 h)	11.8 ± 1.4	425	22

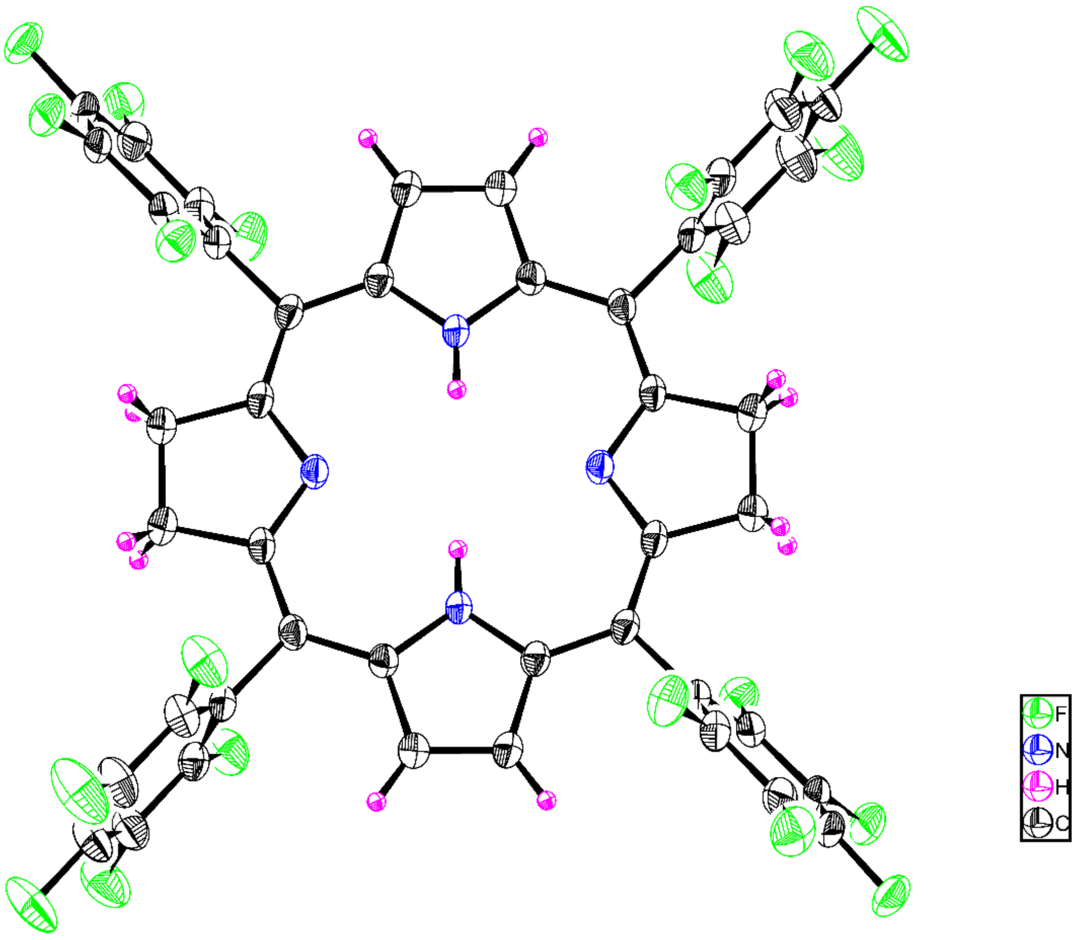

Supplementary Figure 55. Crystal structure of $F_{20}BC$. A ORTEP diagram of $F_{20}BC$ at 50% probability. Cocrystallized solvent molecules are not shown for clarity.

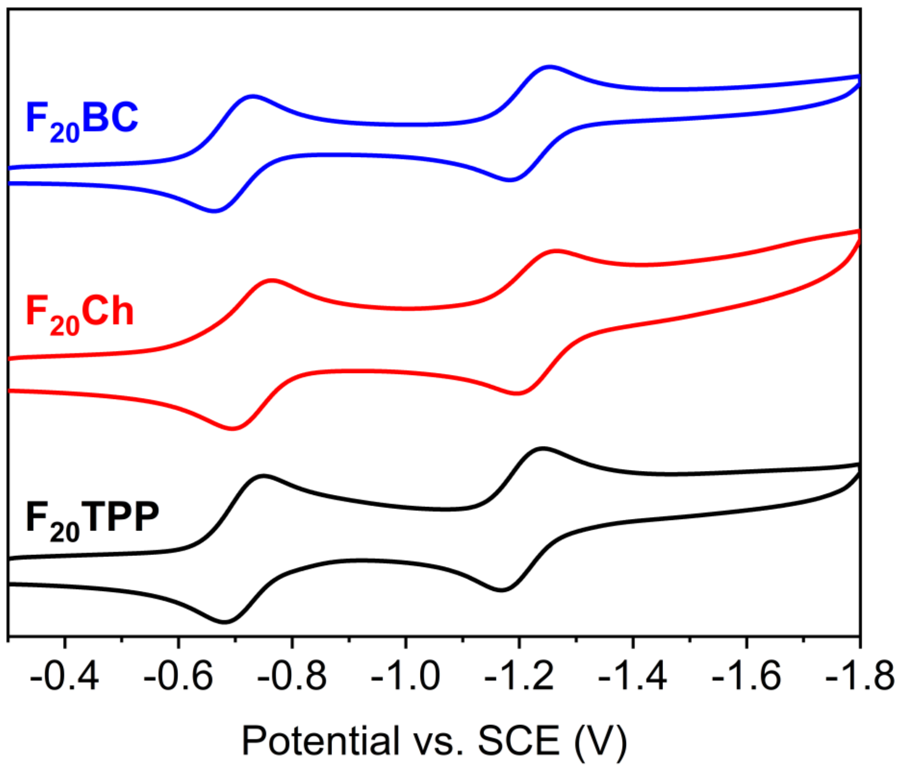

Supplementary Figure 56. Electrochemical study. Cyclic voltammograms for 0.5 mM $F_{20}TPP$ (black), 0.5 mM $F_{20}Ch$ (red), and 0.5 mM $F_{20}BC$ (blue) in DMF containing 0.1 M $TBAPF_6$ at a scan rate of 0.1 $V \cdot s^{-1}$ under N_2.

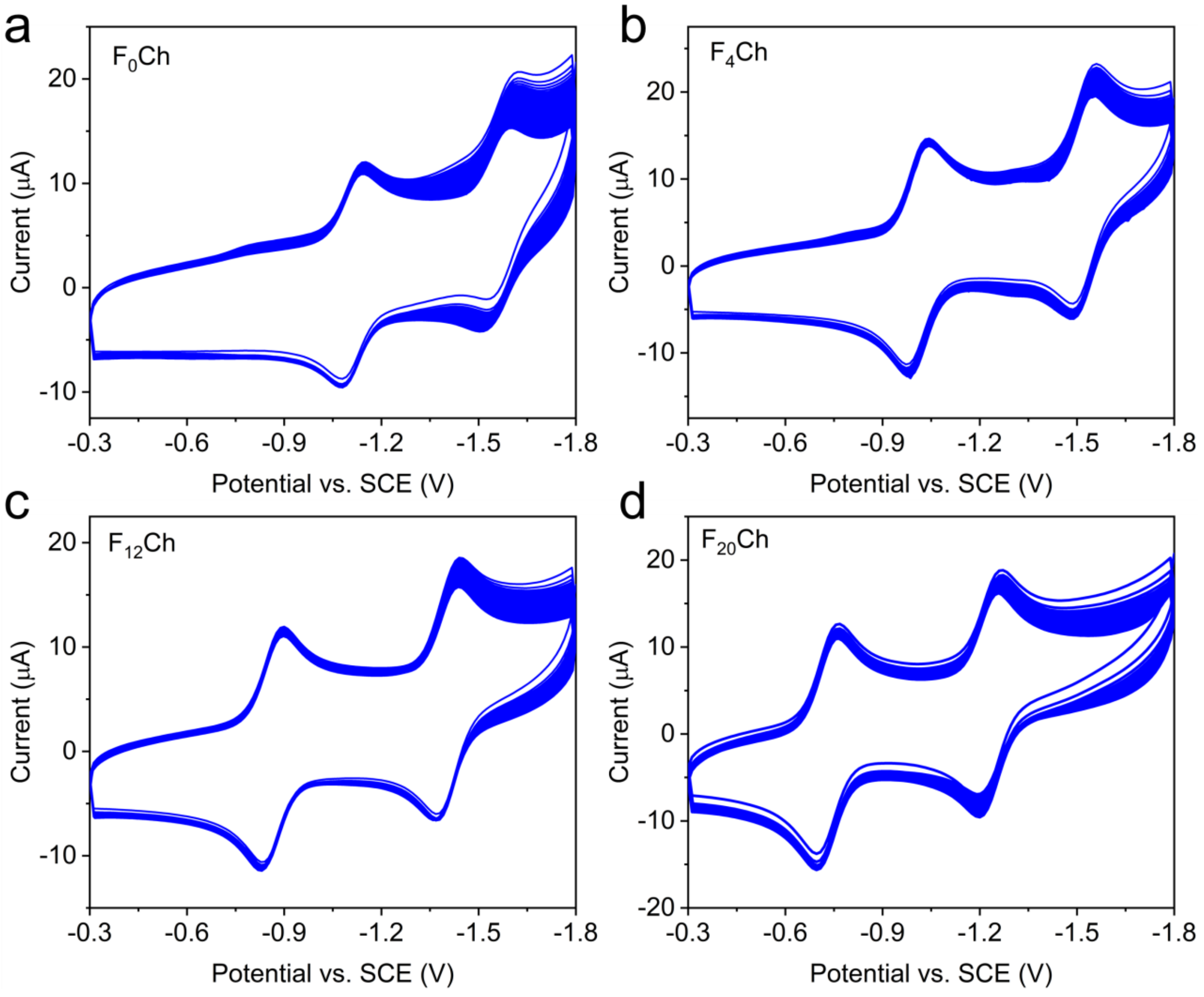

Supplementary Figure 57. Electrochemical study. Cyclic voltammograms for 0.25 mM F_0Ch (a), 0.5 mM F_4Ch (b), 0.5 mM $F_{12}Ch$ (c), and 0.5 mM $F_{20}Ch$ (d) with 1000 cyclic scans in DMF containing 0.1 M $TBAPF_6$ at a scan rate of 0.5 $V{\cdot}s^{-1}$ under N_2.

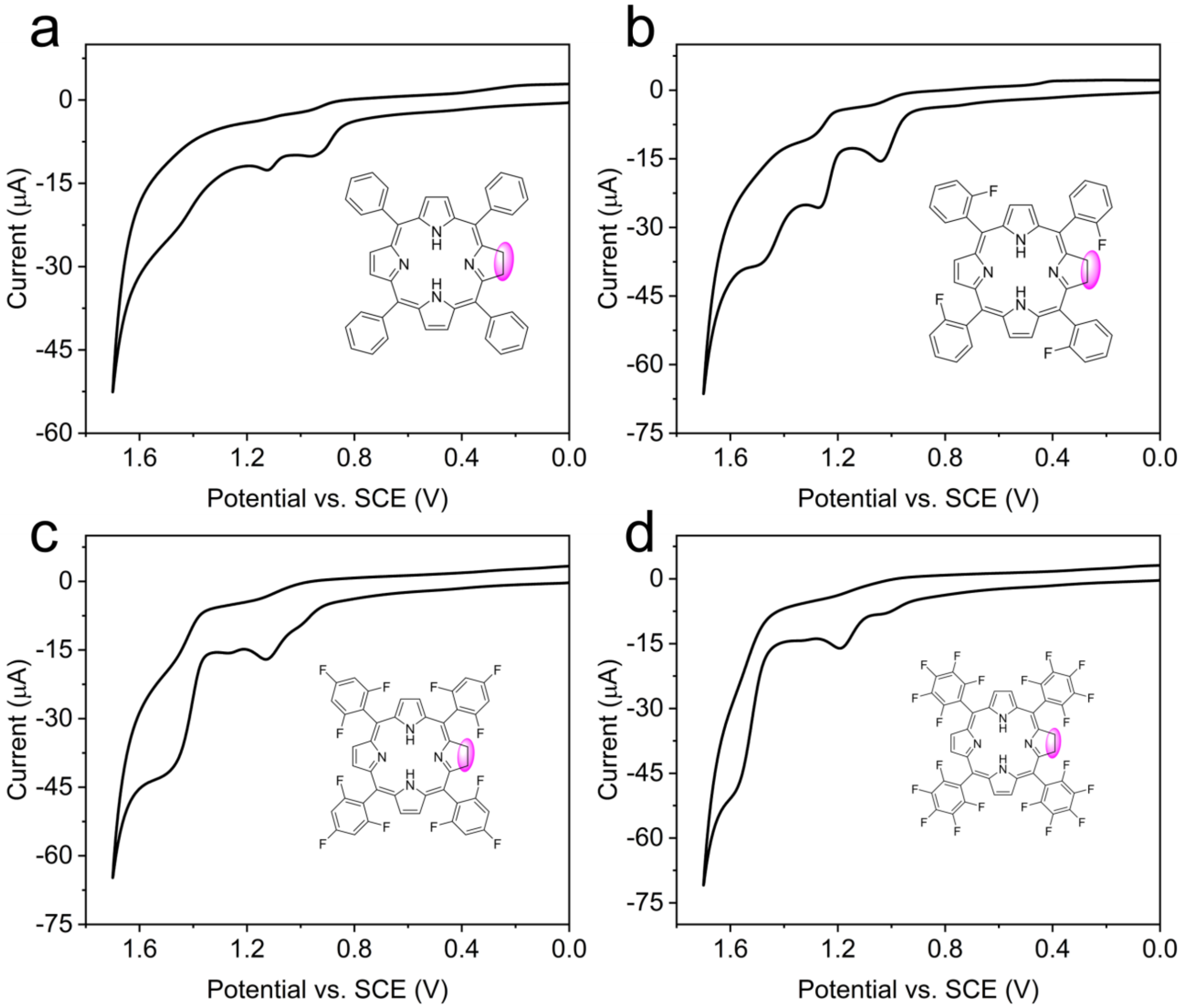

Supplementary Figure 58. Electrochemical study. Oxidative scans of cyclic voltammetry for 0.25 mM F_0Ch (a), 0.5 mM F_4Ch (b), 0.5 mM $F_{12}Ch$ (c), and 0.5 mM $F_{20}Ch$ (d) in DMF containing 0.1 M $TBAPF_6$ at a scan rate of 0.1 $V \cdot s^{-1}$ under N_2.

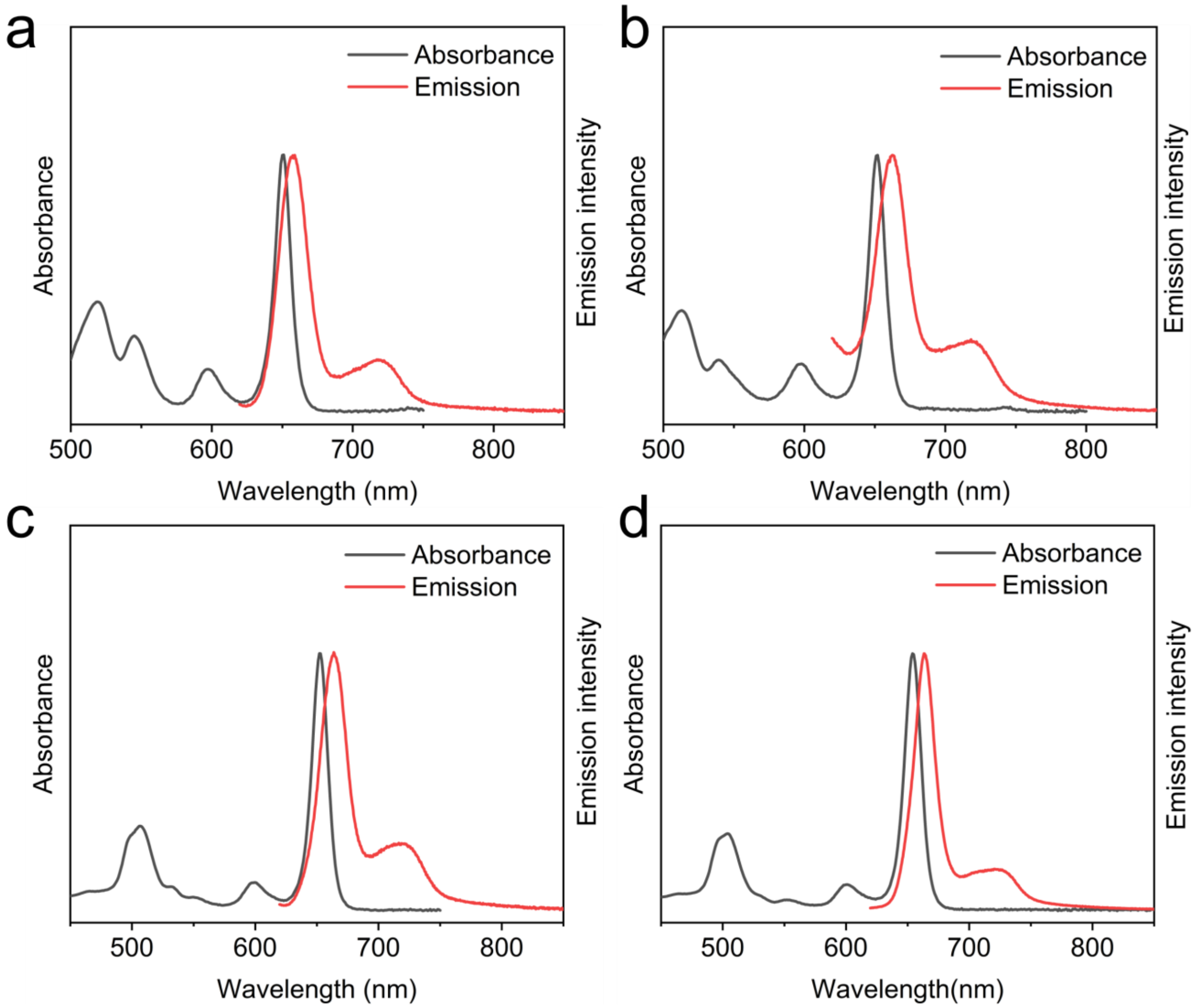

Supplementary Figure 59. UV-vis and emission spectra. Normalized emission (λ_{ex} = 600 nm) and absorbance spectra of F_0Ch (a), F_4Ch (b), $F_{12}Ch$ (c), and $F_{20}Ch$ (d) at 298 K in DMF.

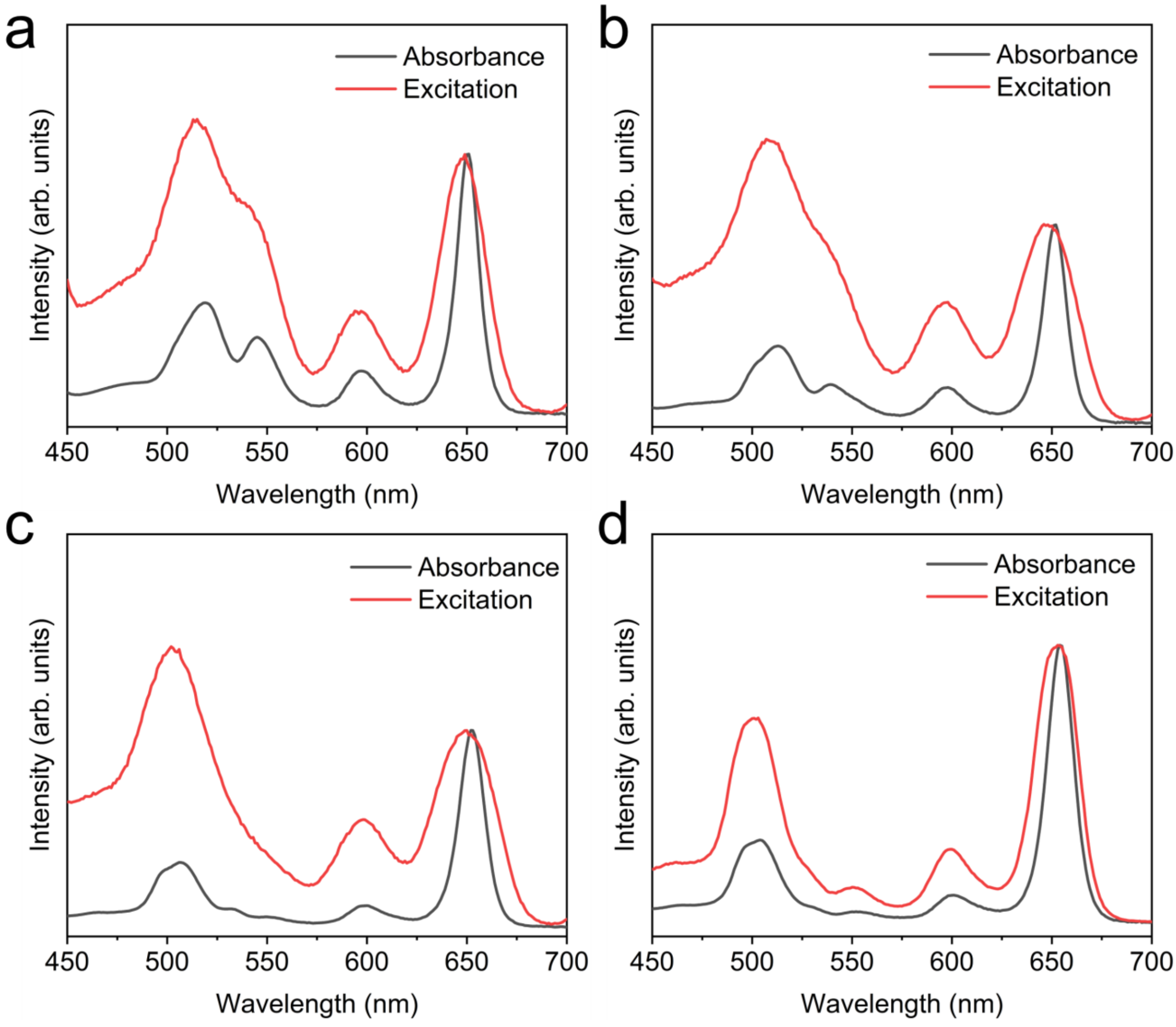

Supplementary Figure 60. UV-vis and excitation spectra. Normalized excitation and absorbance spectra of F_0Ch (a), F_4Ch (b), $F_{12}Ch$ (c), and $F_{20}Ch$ (d) at 298 K in DMF.

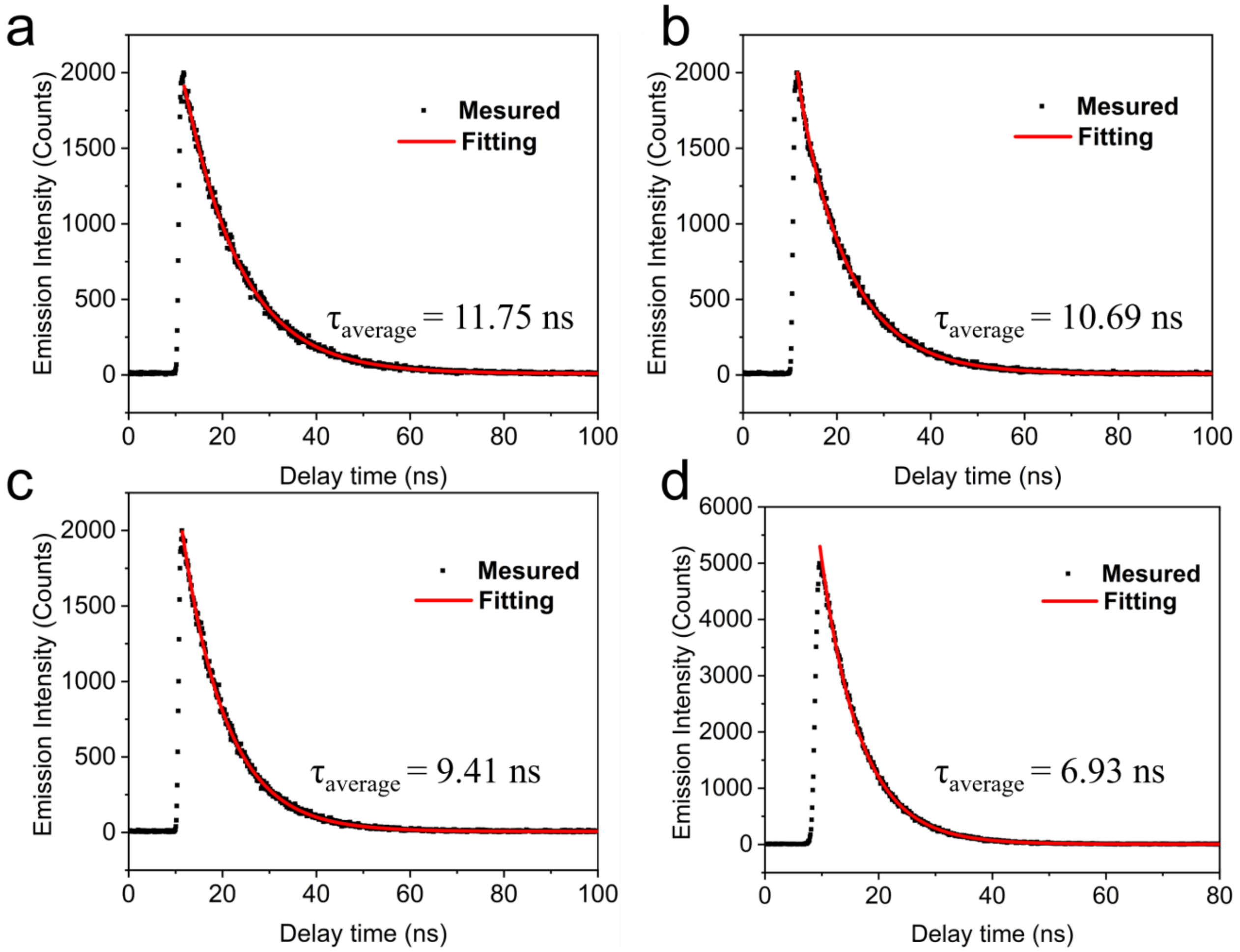

Supplementary Figure 61. Emission decay. Emission decay of 40 µM F_0Ch (a), 40 µM F_4Ch (b), 40 µM $F_{12}Ch$ (c), and 20 µM $F_{20}Ch$ (d) in DMF under N_2 at 298K. The decay curve of F_0Ch and F_4Ch was fitted with a double exponential equation ($I(\tau) = A_1 exp(-\tau/\tau_1)) + A_2 exp(-\tau/\tau_2)$) and the decay curve of $F_{12}Ch$ and $F_{20}Ch$ was fitted with a single exponential equation ($I(\tau) = A_1 exp(-\tau/\tau_1)$) (parameters in the table below).

PS	Lifetime (ns) τ_1	Lifetime (ns) τ_2	Coefficient A_1	Coefficient A_2	Lifetime average (ns) $\tau_{average}$
F_0Ch	2.0169	11.6900	-74.1901	1989.6708	11.75
F_4Ch	0.7200	10.7076	60.0638	1957.4949	10.69
$F_{12}Ch$	9.4114	-	2005.3789	-	9.41
$F_{20}Ch$	6.9291	-	5363.903	-	6.93

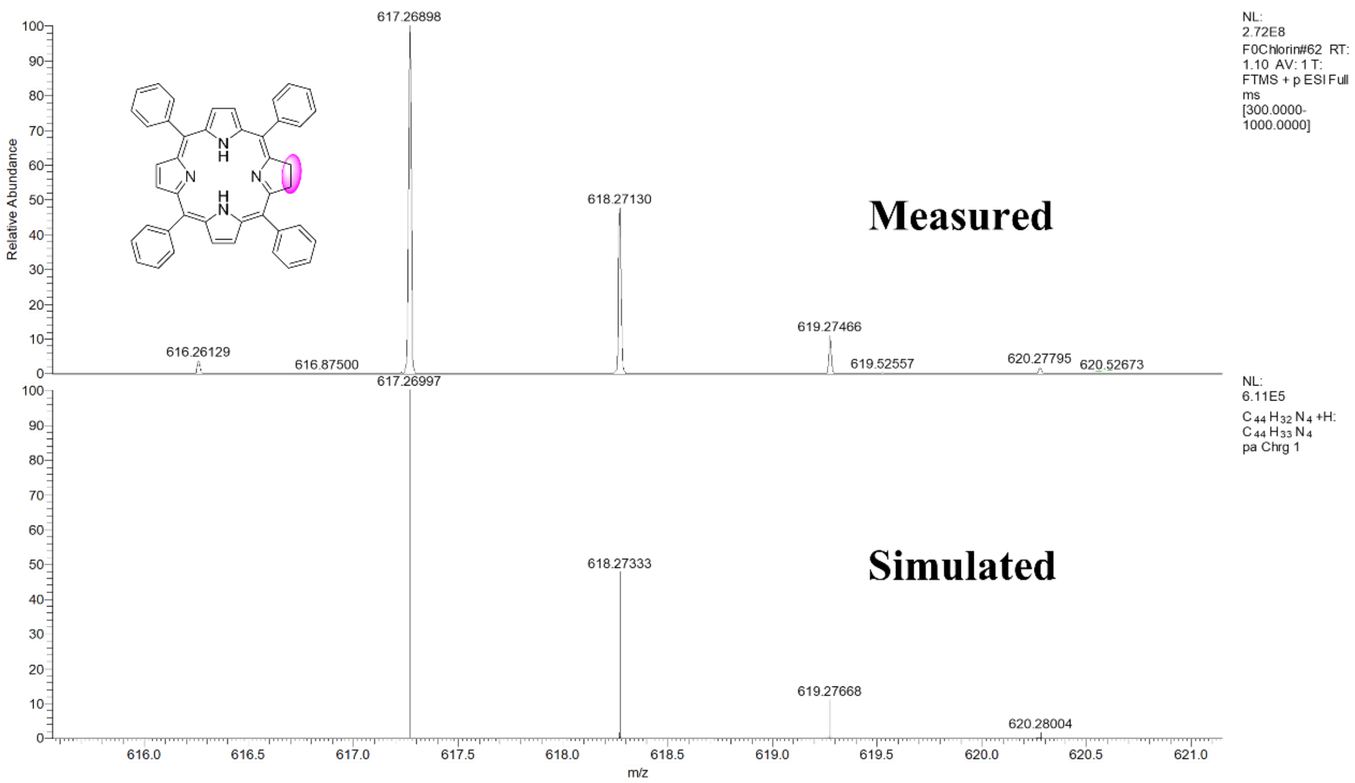

Supplementary Figure 62. HRMS spectrum. HRMS spectrum of F_0Ch in CH_2Cl_2 and CH_3OH (positive ion mode).

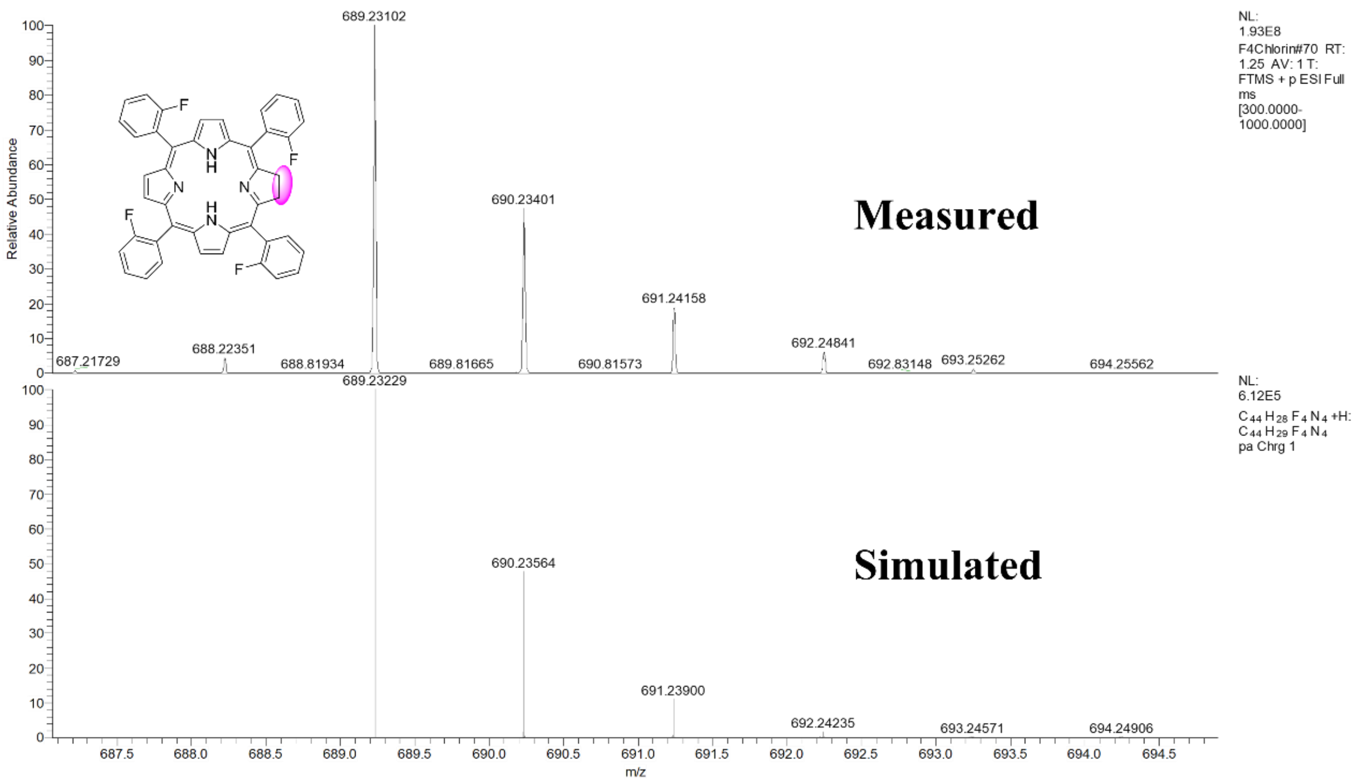

Supplementary Figure 63. HRMS spectrum. HRMS spectrum of F_4Ch in CH_2Cl_2 and CH_3OH (positive ion mode).

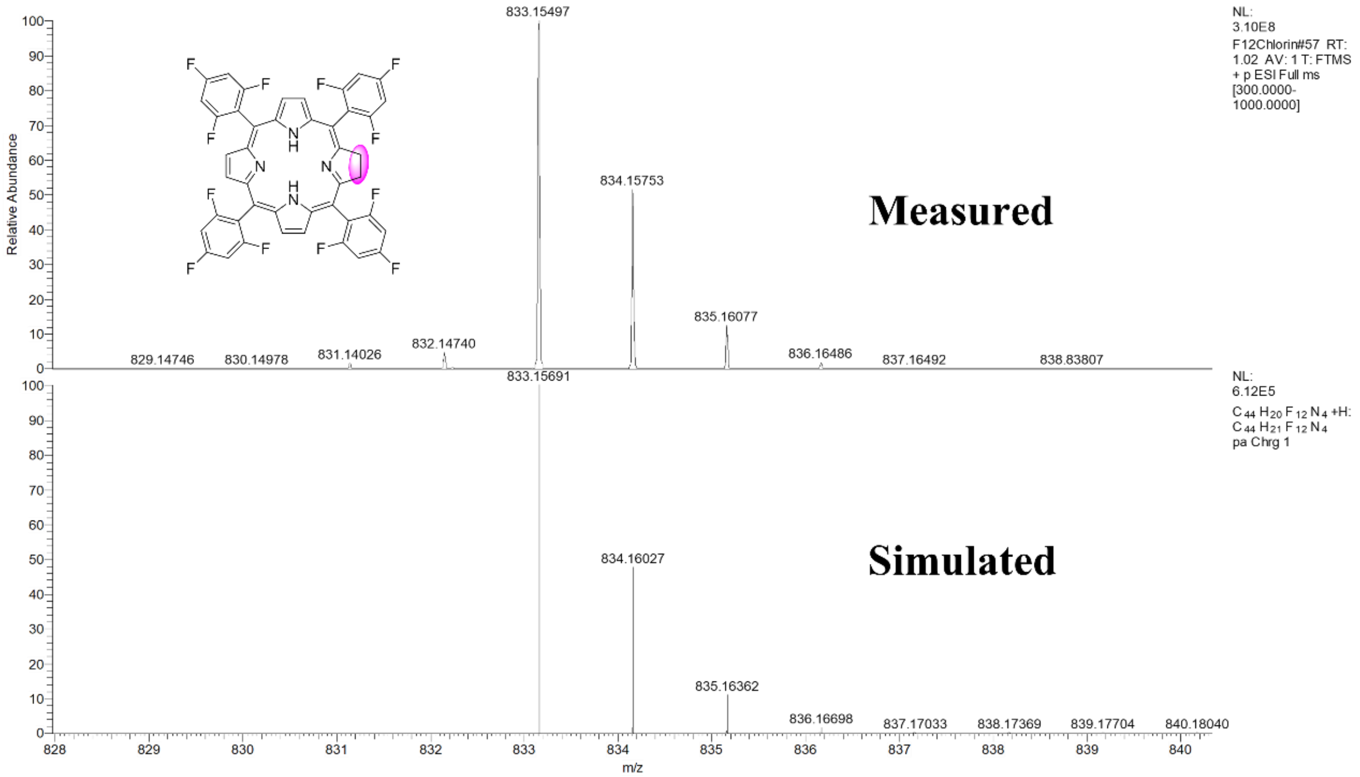

Supplementary Figure 64. HRMS spectrum. HRMS spectrum of $F_{12}Ch$ in CH_2Cl_2 and CH_3OH (positive ion mode).

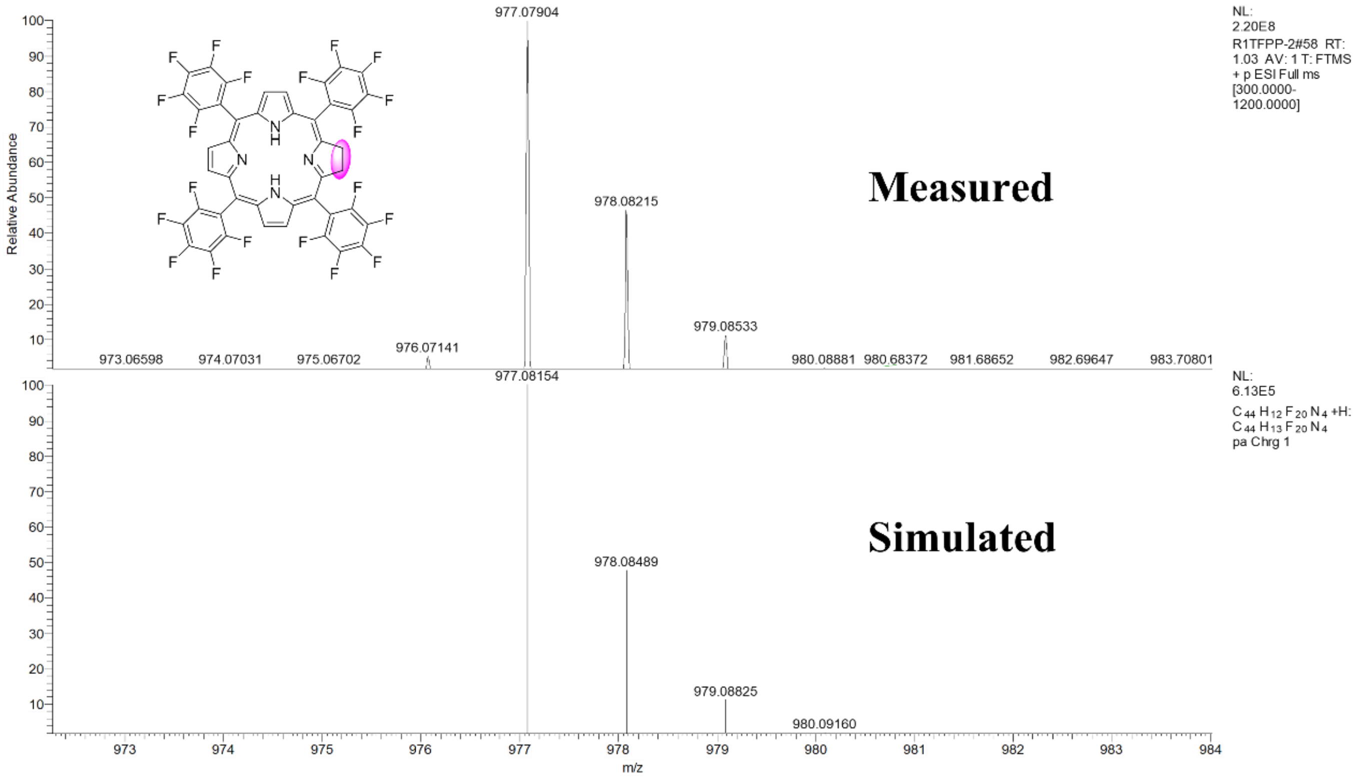

Supplementary Figure 65. HRMS spectrum. HRMS spectrum of $F_{20}Ch$ in CH_2Cl_2 and CH_3OH (positive ion mode).

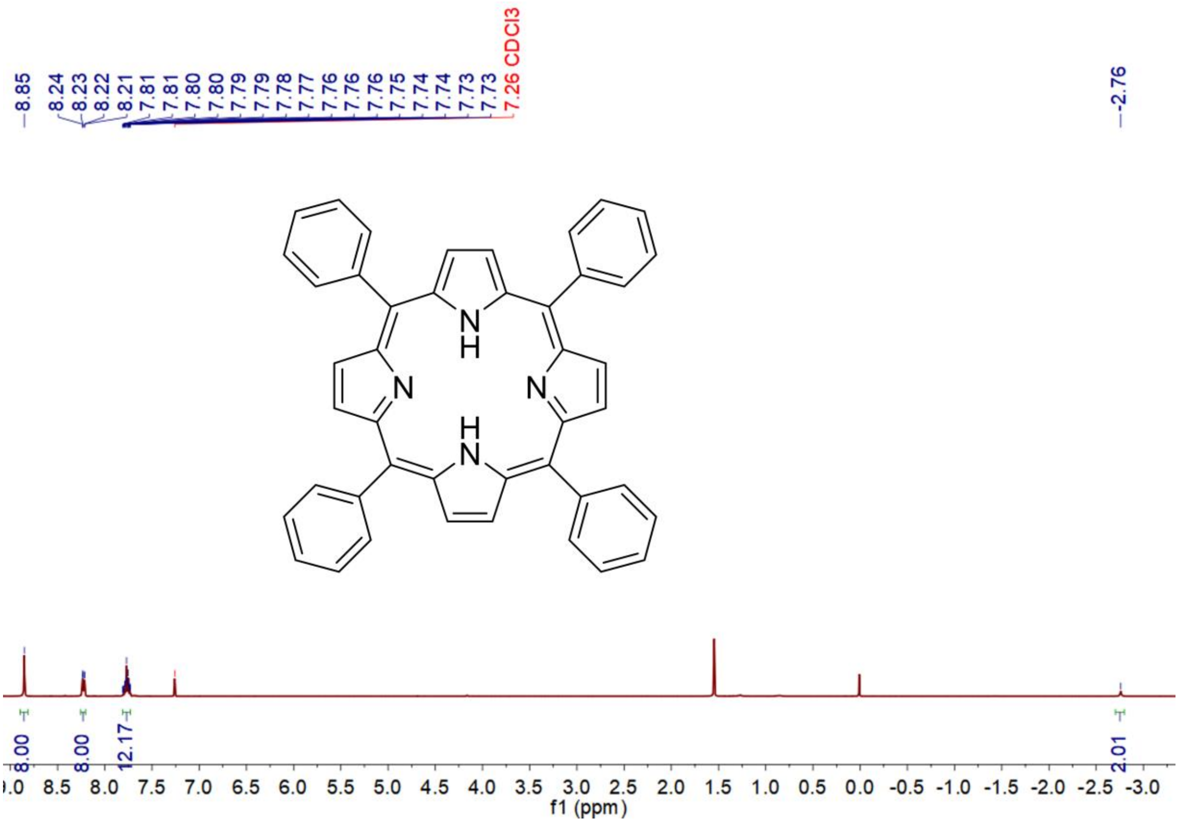

Supplementary Figure 66. ^{1}H NMR spectrum. ^{1}H NMR spectrum of F_0TPP in $CDCl_3$.

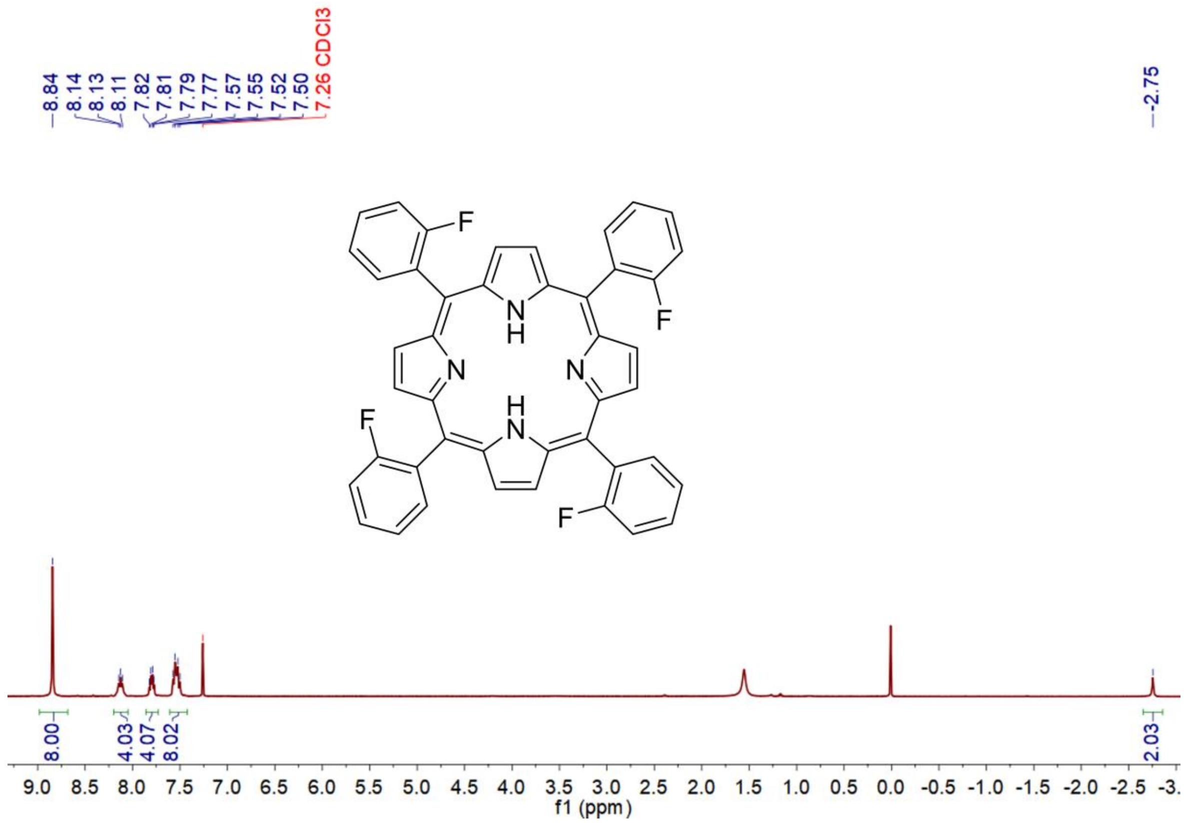

Supplementary Figure 67. ^{1}H NMR spectrum. ^{1}H NMR spectrum of F_4TPP in $CDCl_3$.

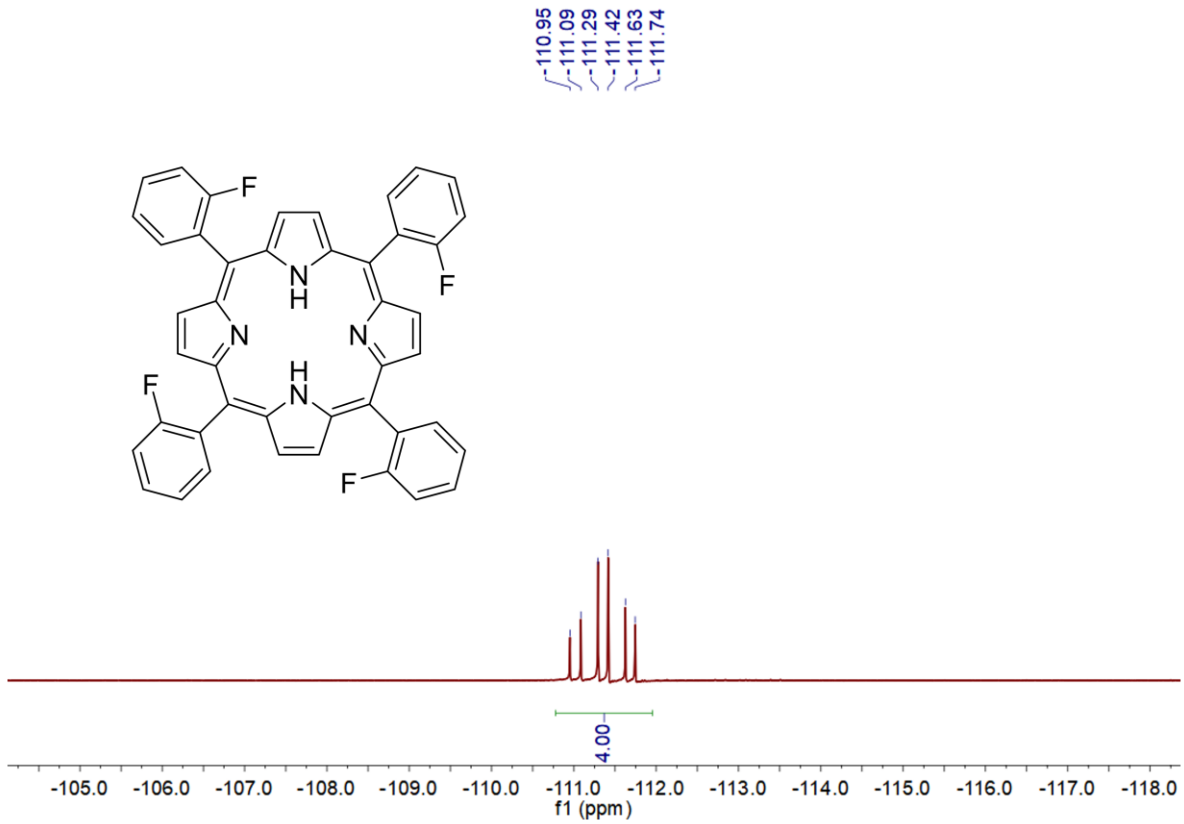

Supplementary Figure 68. ^{19}F NMR spectrum. ^{19}F NMR spectrum of F_4TPP in $CDCl_3$.

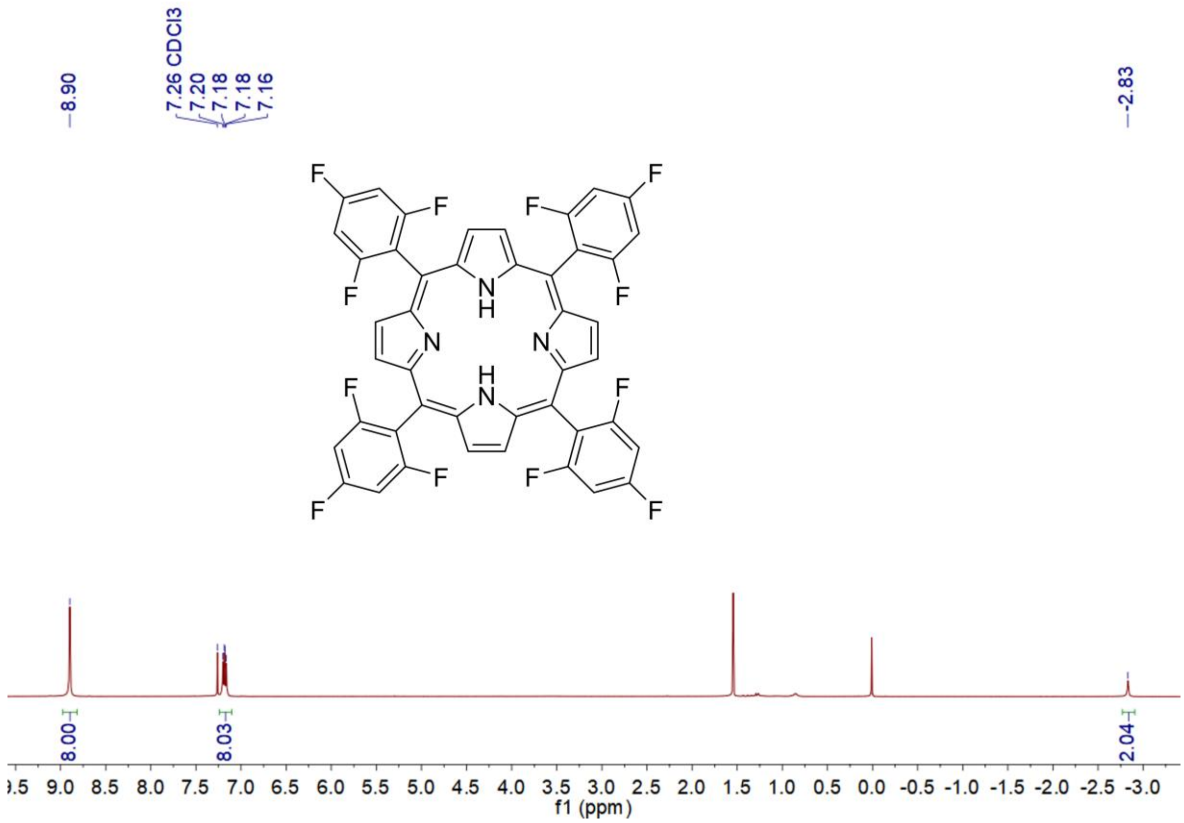

Supplementary Figure 69. ^{1}H NMR spectrum. ^{1}H NMR spectrum of F_{12}TPP in $CDCl_3$.

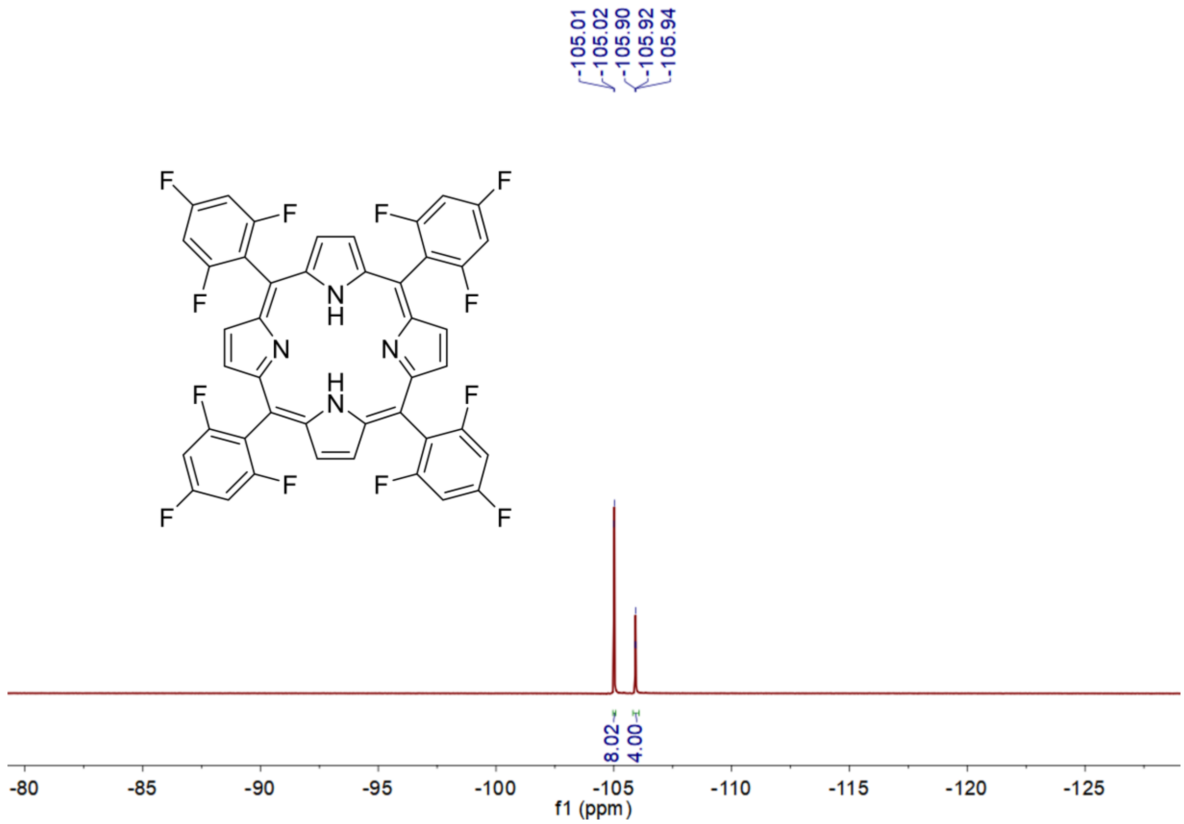

Supplementary Figure 70. ^{19}F NMR spectrum. ^{19}F NMR spectrum of F_{12}TPP in $CDCl_3$.

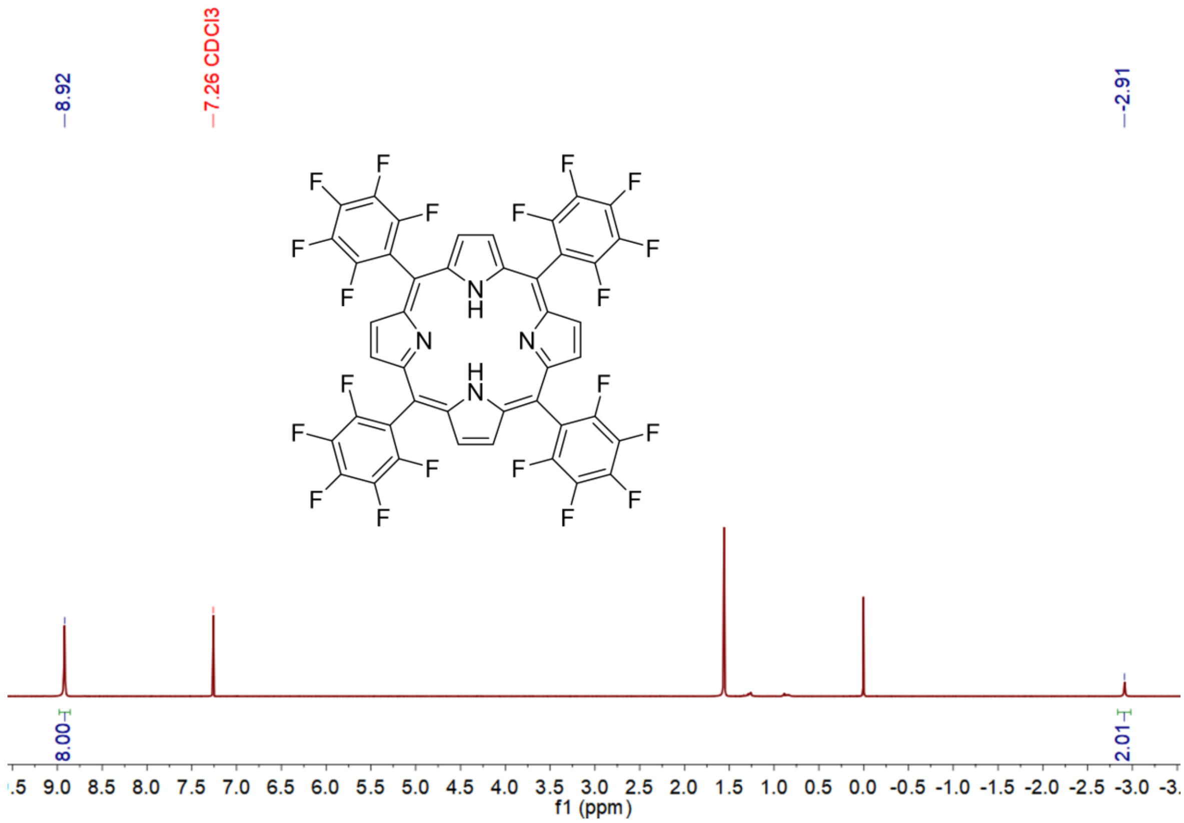

Supplementary Figure 71. ^{1}H NMR spectrum. ^{1}H NMR spectrum of F_{20}TPP in $CDCl_3$.

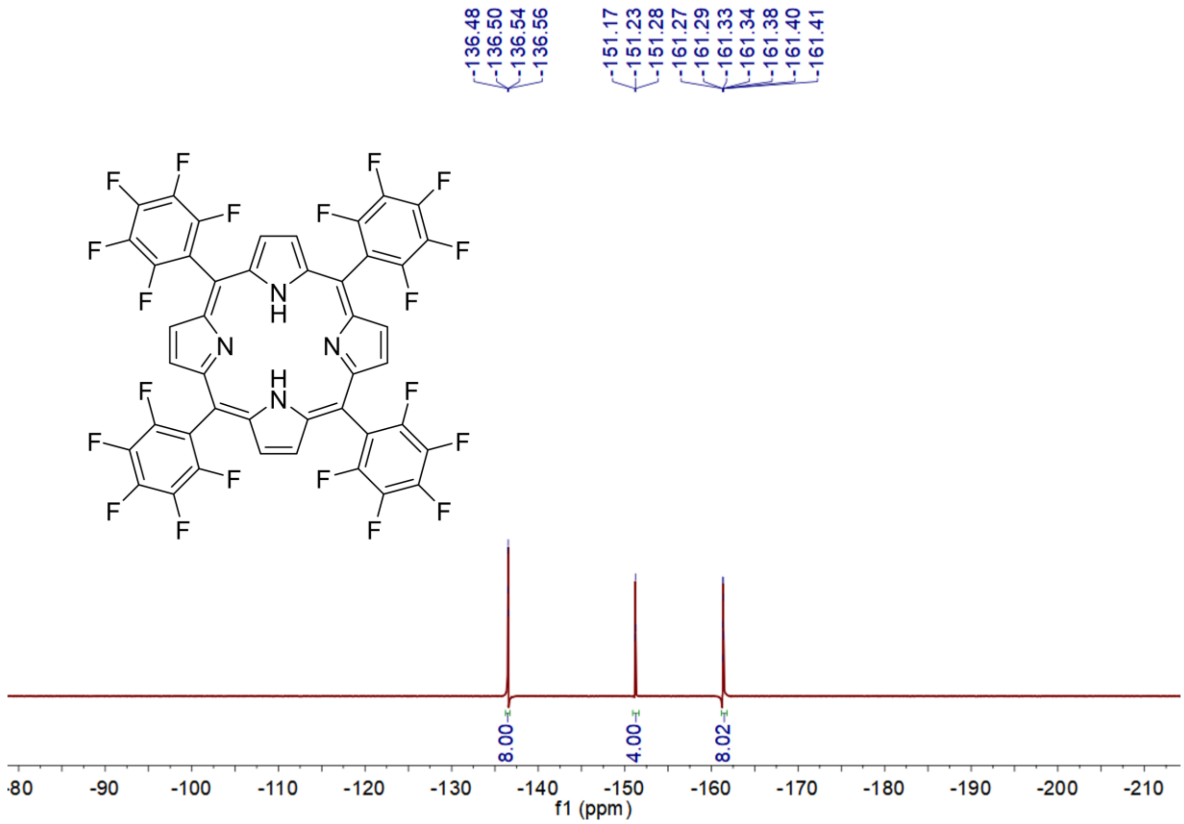

Supplementary Figure 72. ^{19}F NMR spectrum. ^{19}F NMR spectrum of F_{20}TPP in $CDCl_3$.

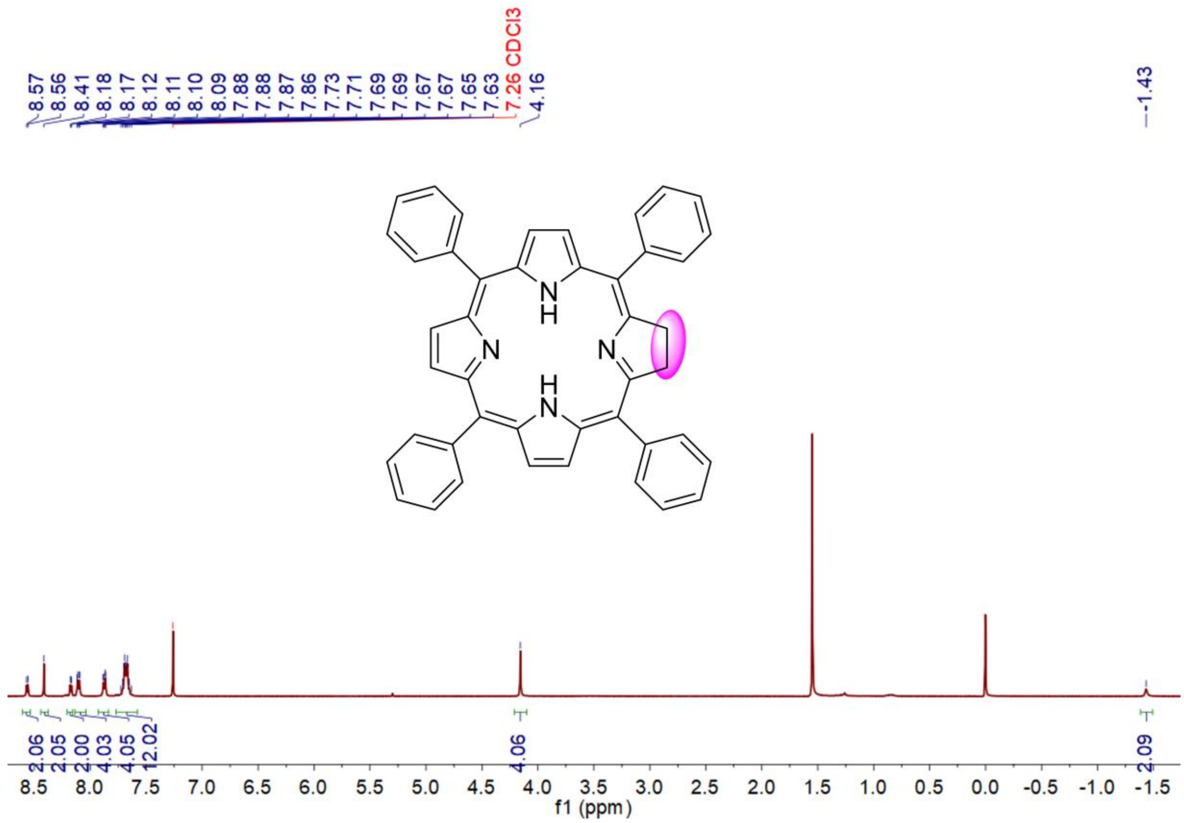

Supplementary Figure 73. ^{1}H NMR spectrum. ^{1}H NMR spectrum of F_0Ch in $CDCl_3$.

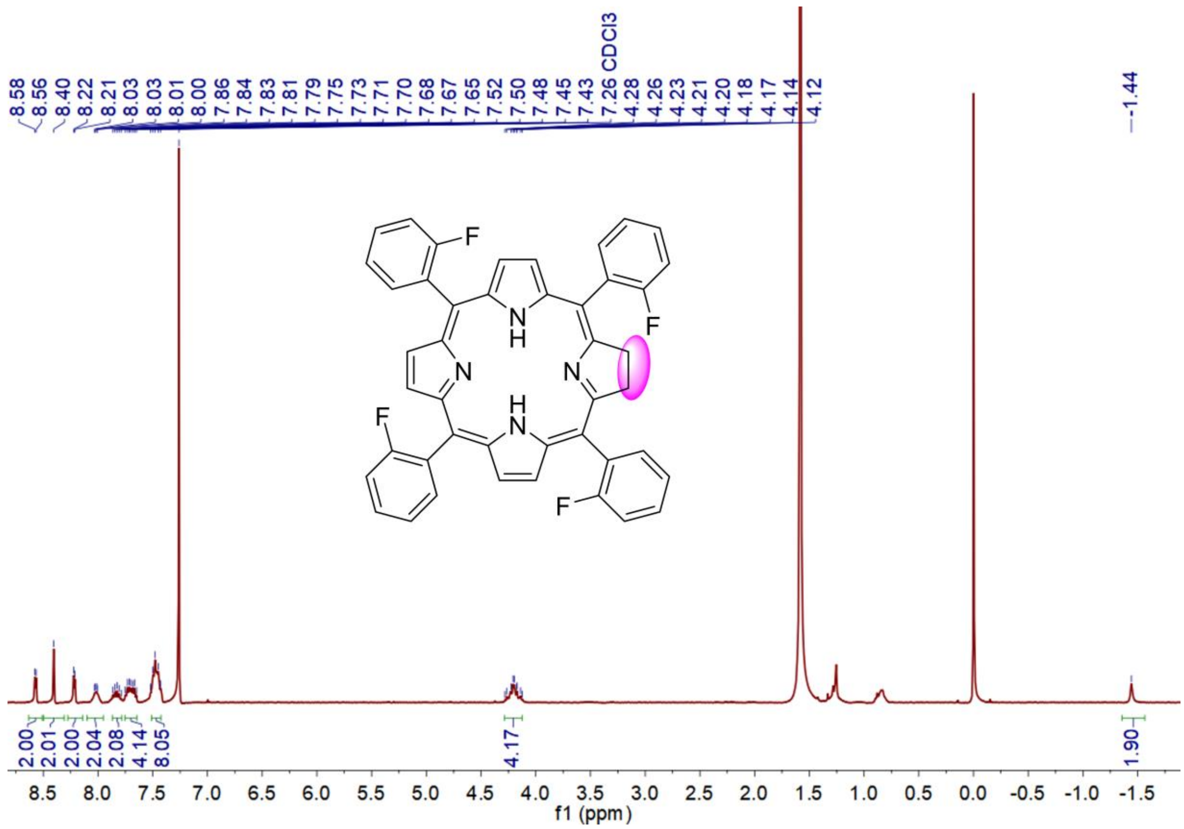

Supplementary Figure 74. ^{1}H NMR spectrum. ^{1}H NMR spectrum of F_4Ch in $CDCl_3$.

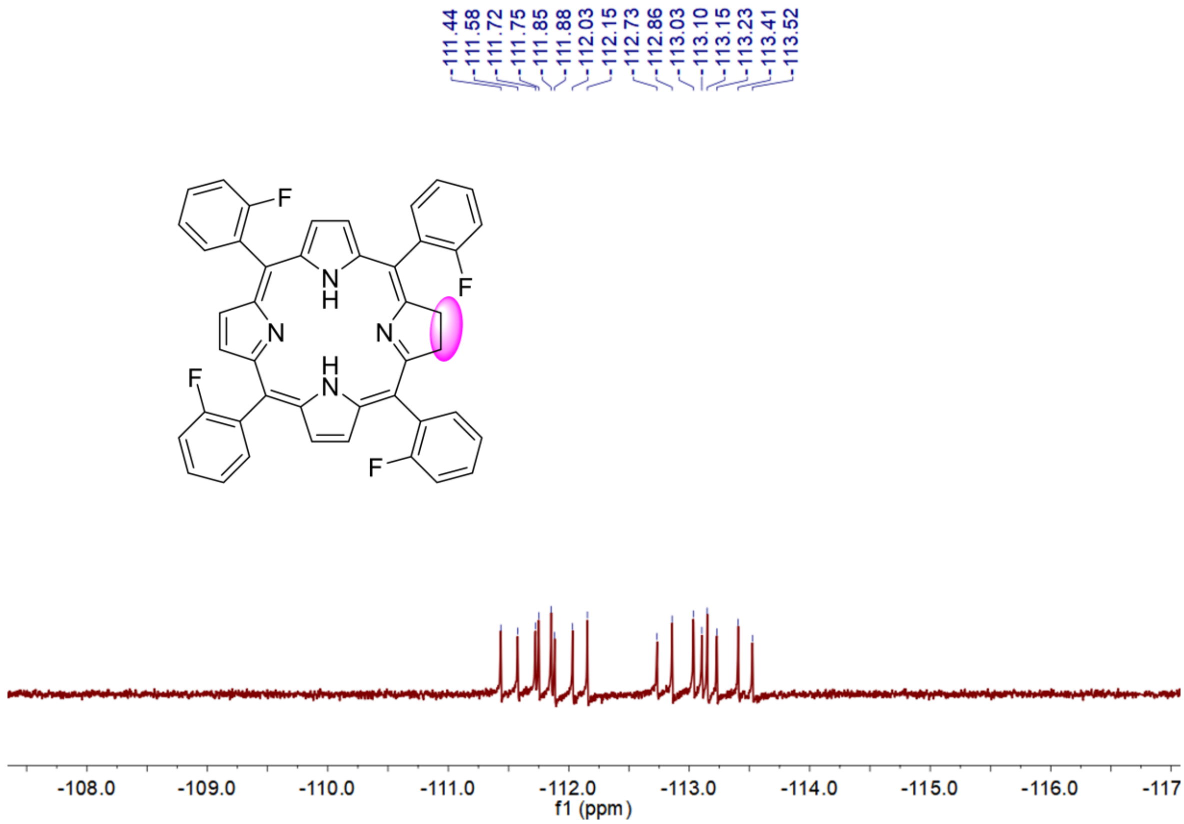

Supplementary Figure 75. ^{19}F NMR spectrum. ^{19}F NMR spectrum of F_4Ch in $CDCl_3$.

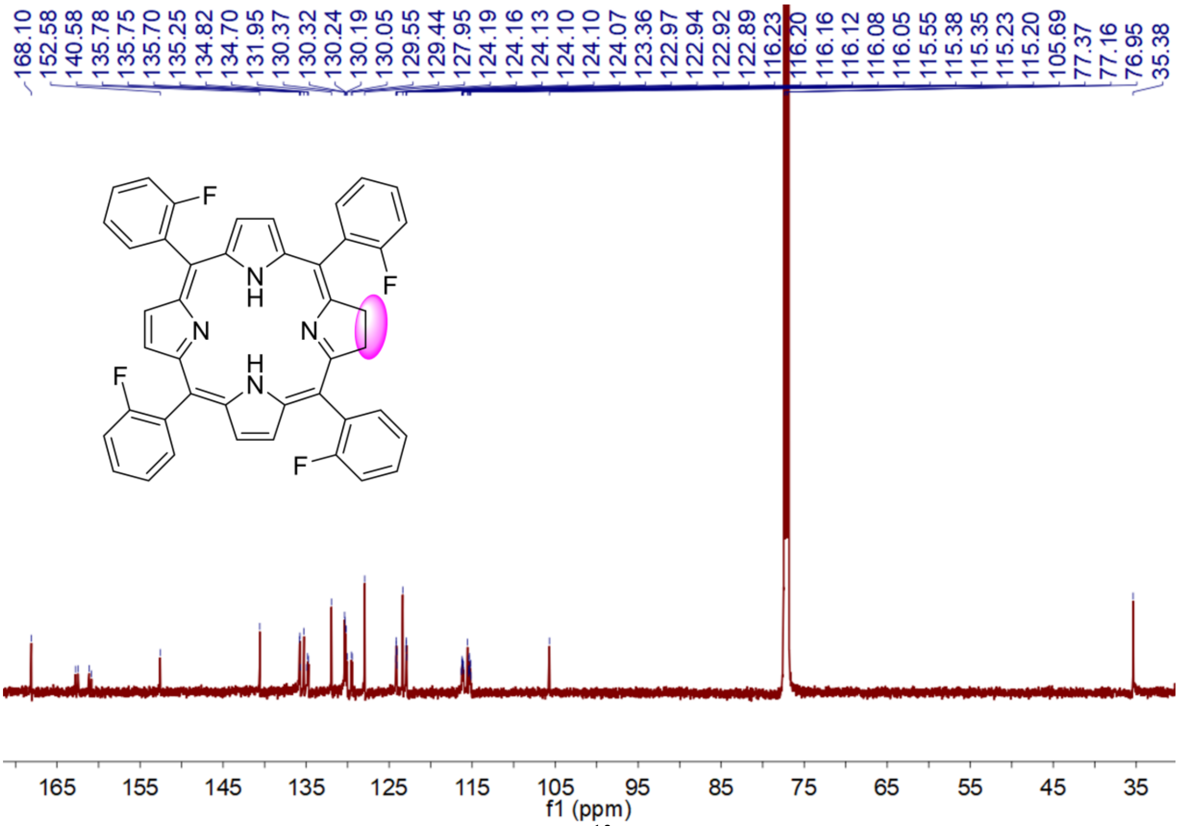

Supplementary Figure 76. ^{13}C NMR spectrum. ^{13}C NMR spectrum of F_4Ch in $CDCl_3$.

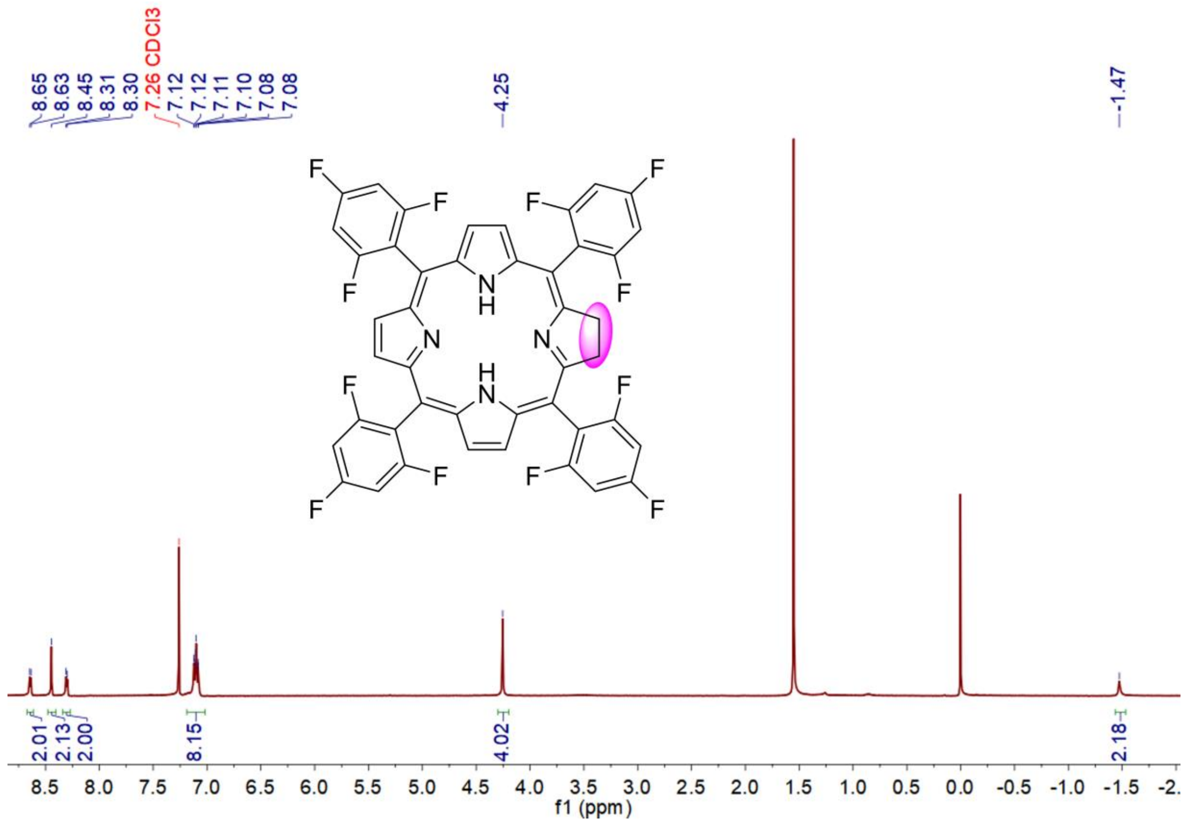

Supplementary Figure 77. ^{1}H NMR spectrum. ^{1}H NMR spectrum of $F_{12}Ch$ in $CDCl_3$.

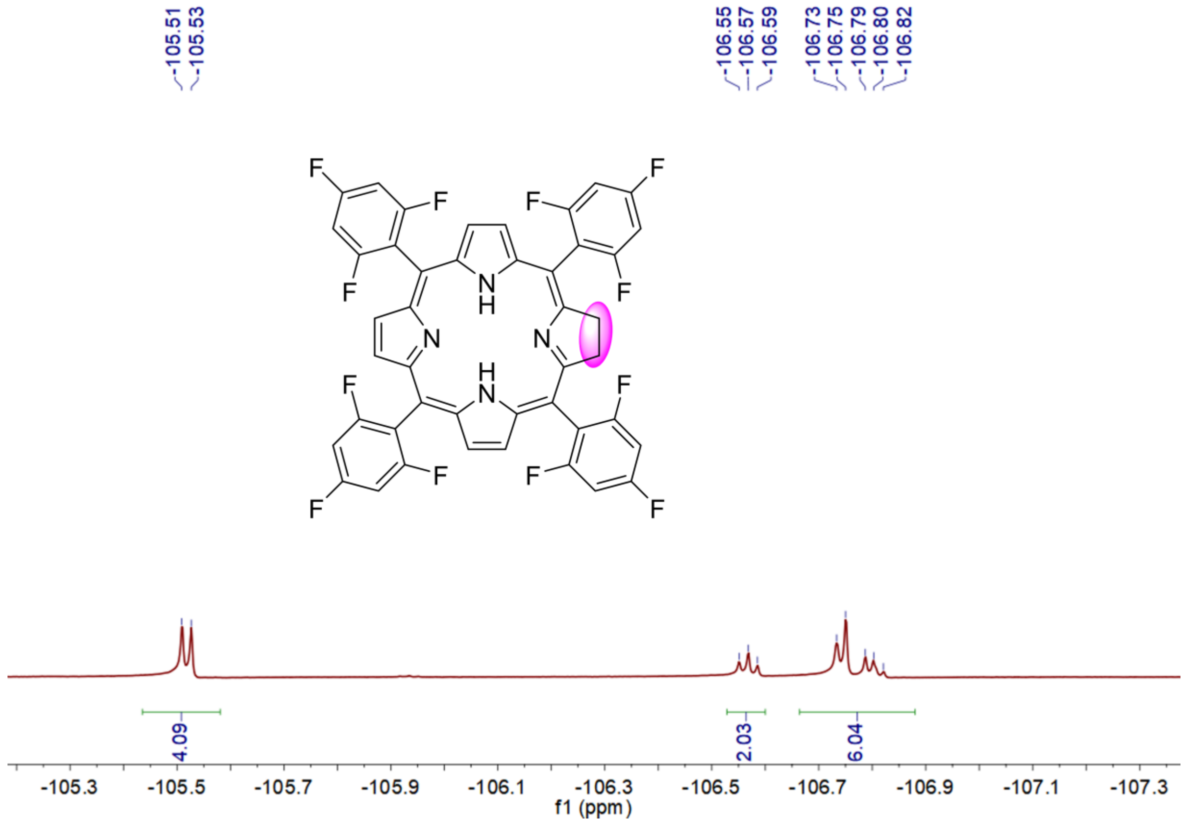

Supplementary Figure 78. ^{19}F NMR spectrum. ^{19}F NMR spectrum of $F_{12}Ch$ in $CDCl_3$.

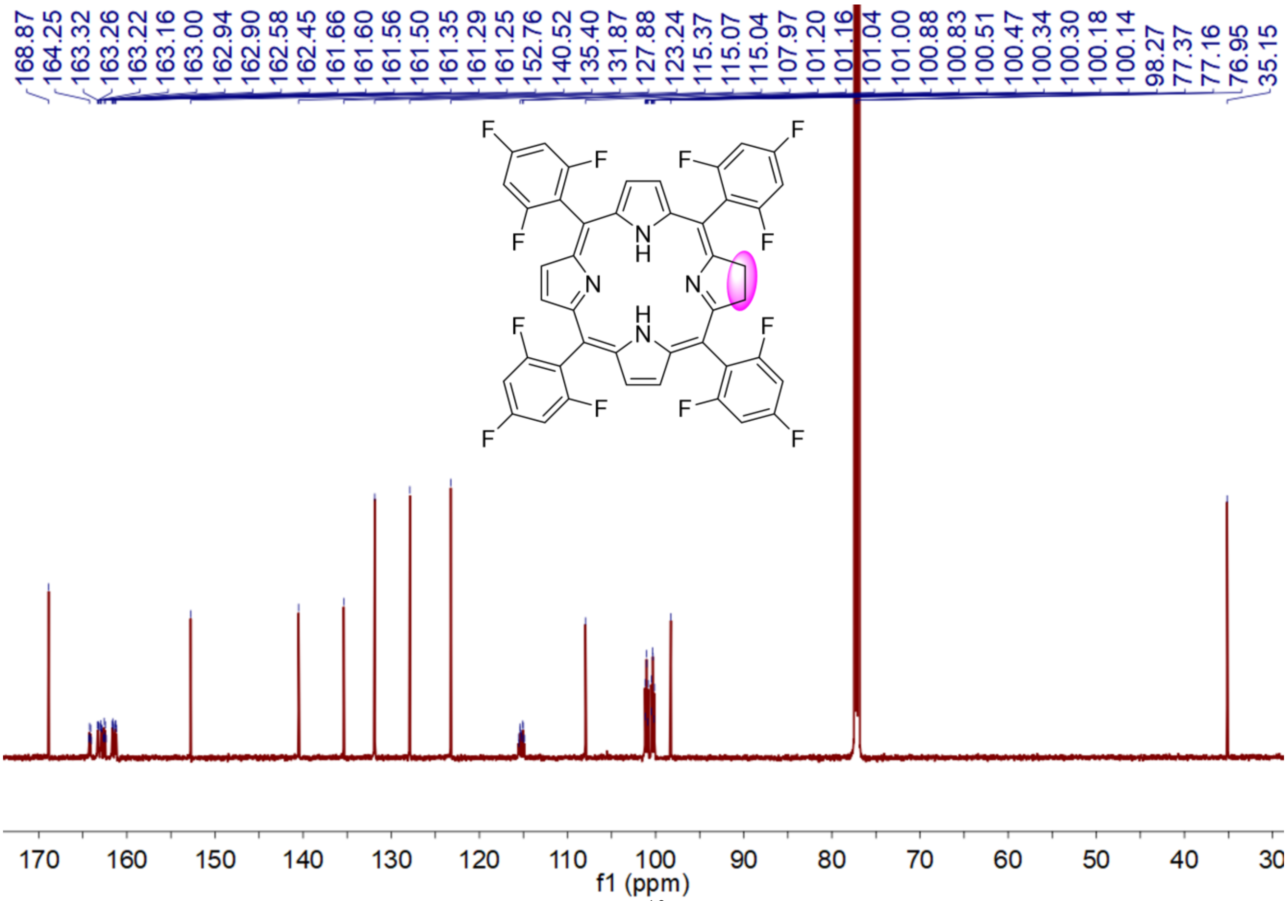

Supplementary Figure 79. ^{13}C NMR spectrum. ^{13}C NMR spectrum of F_{12}Ch in $CDCl_3$.

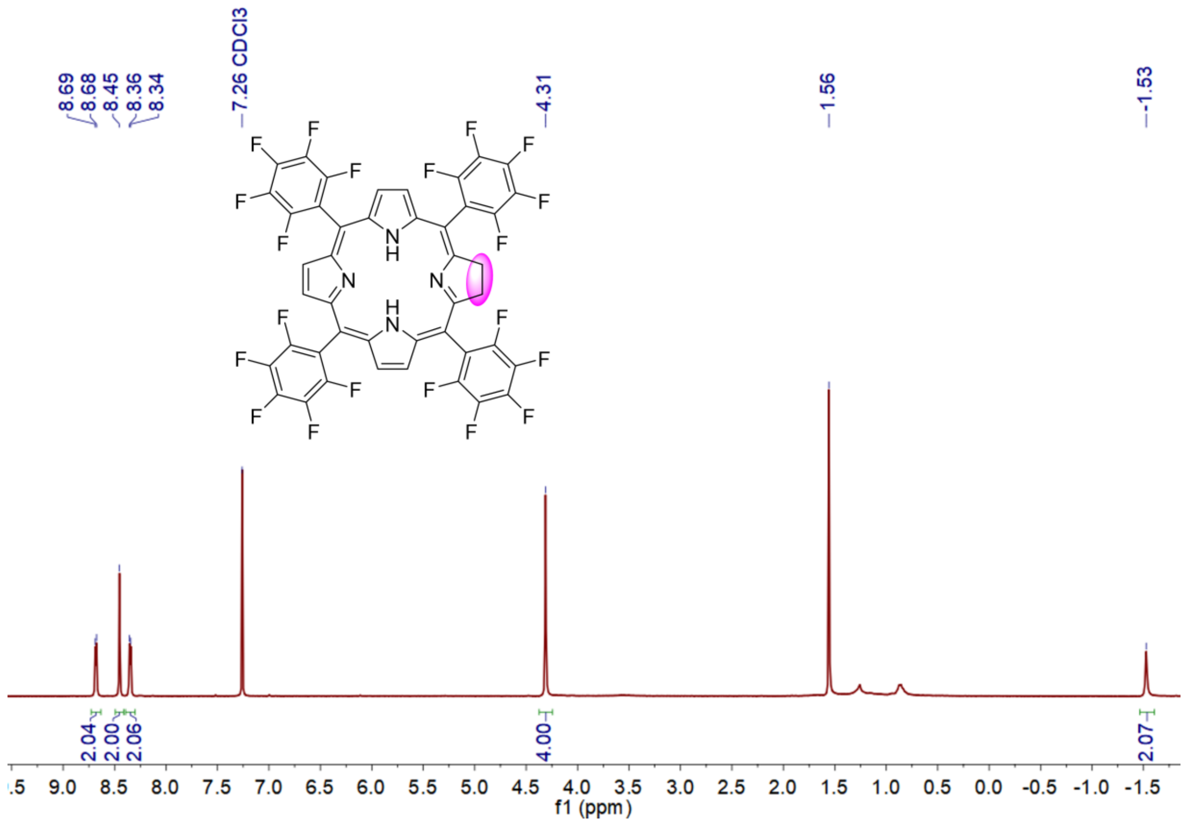

Supplementary Figure 80. ¹H NMR spectrum. ¹H NMR spectrum of $F_{20}Ch$ in $CDCl_3$.

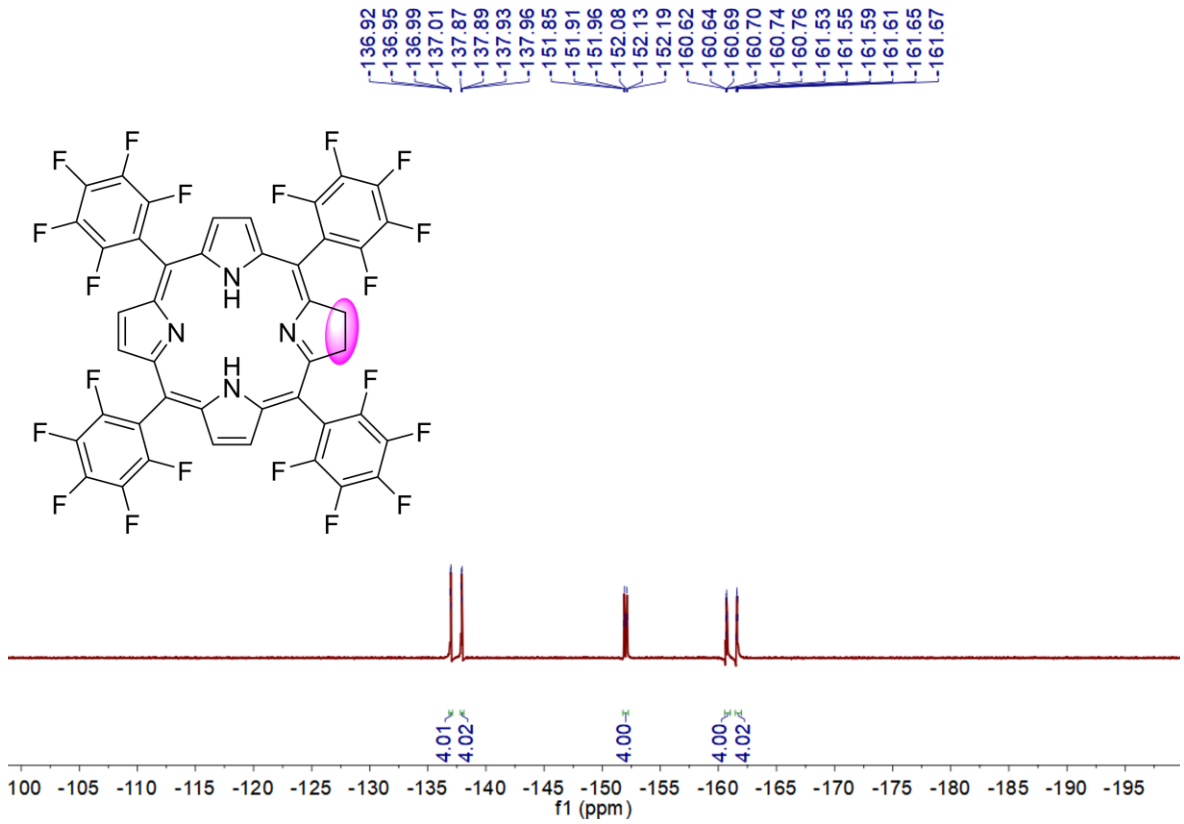

Supplementary Figure 81. ¹⁹F NMR spectrum. ¹⁹F NMR spectrum of $F_{20}Ch$ in $CDCl_3$.

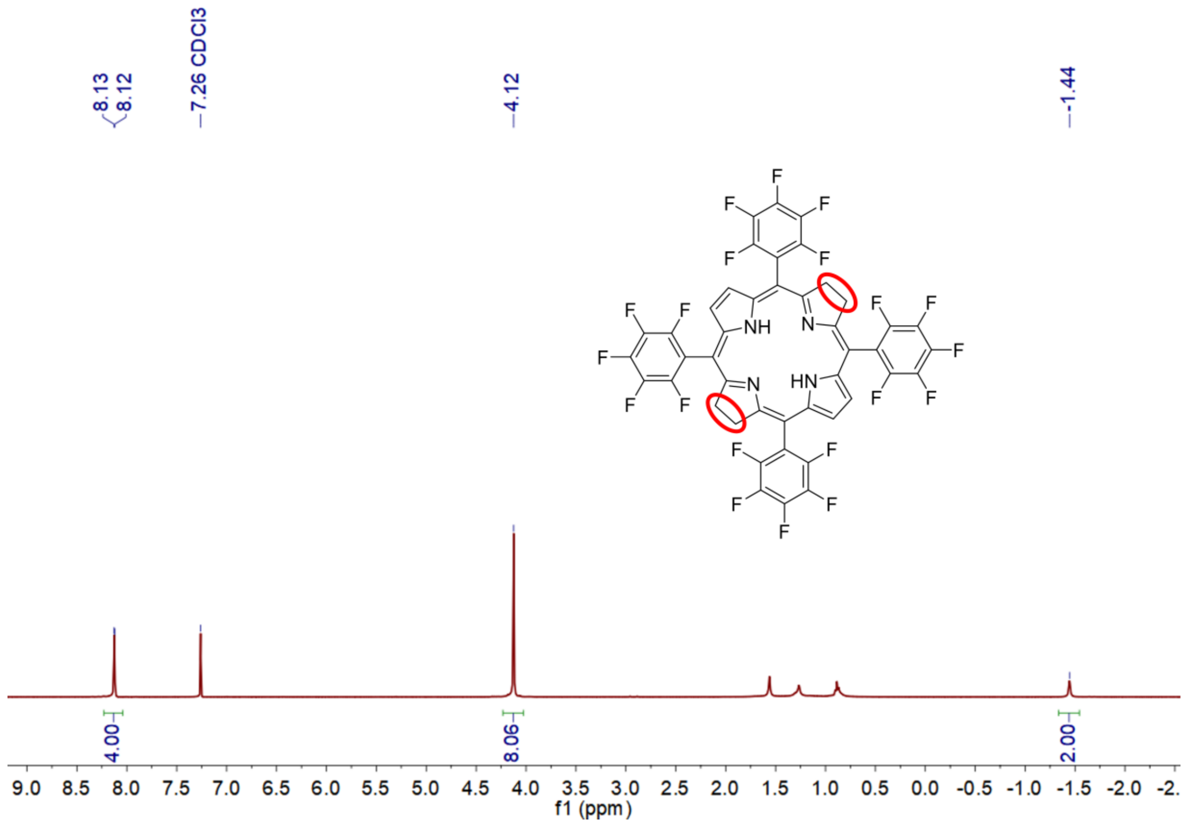

Supplementary Figure 82. ^{1}H NMR spectrum. ^{1}H NMR spectrum of F_{20}BC in $CDCl_3$.

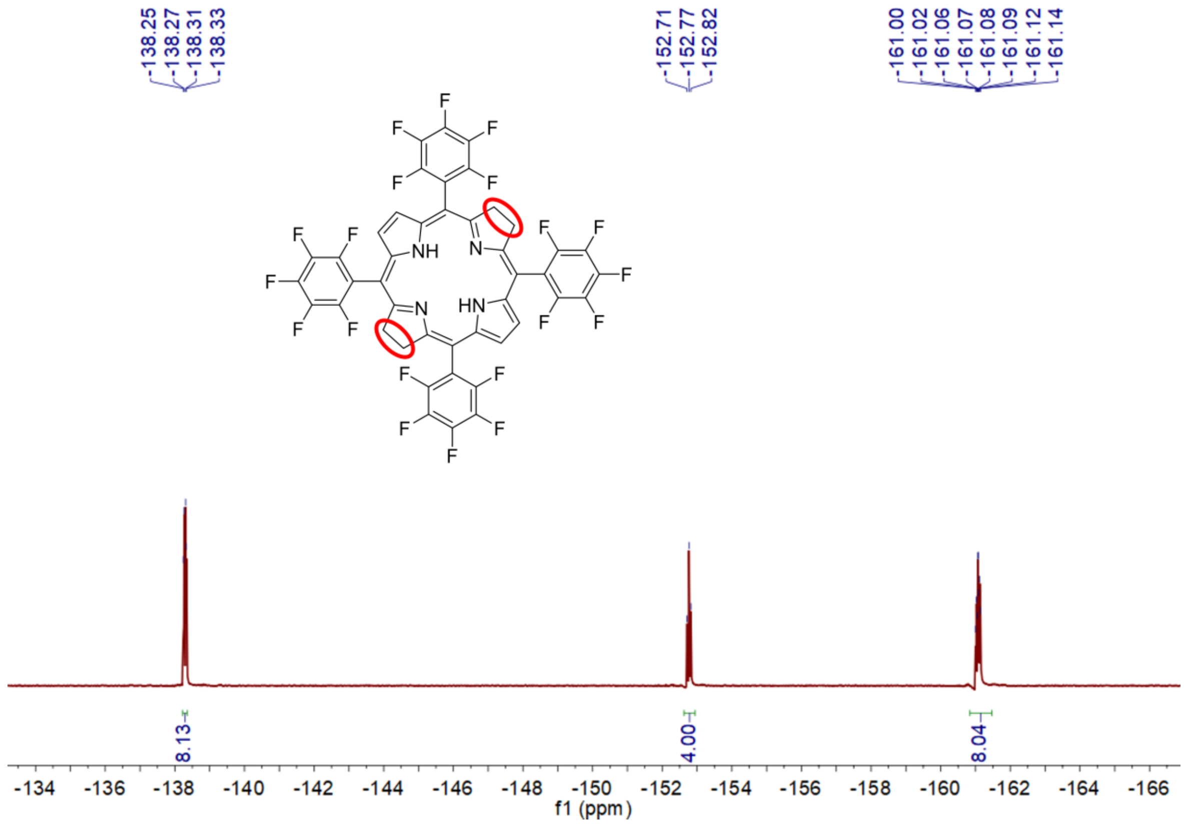

Supplementary Figure 83. ^{19}F NMR spectrum. ^{19}F NMR spectrum of F_{20}BC in $CDCl_3$.

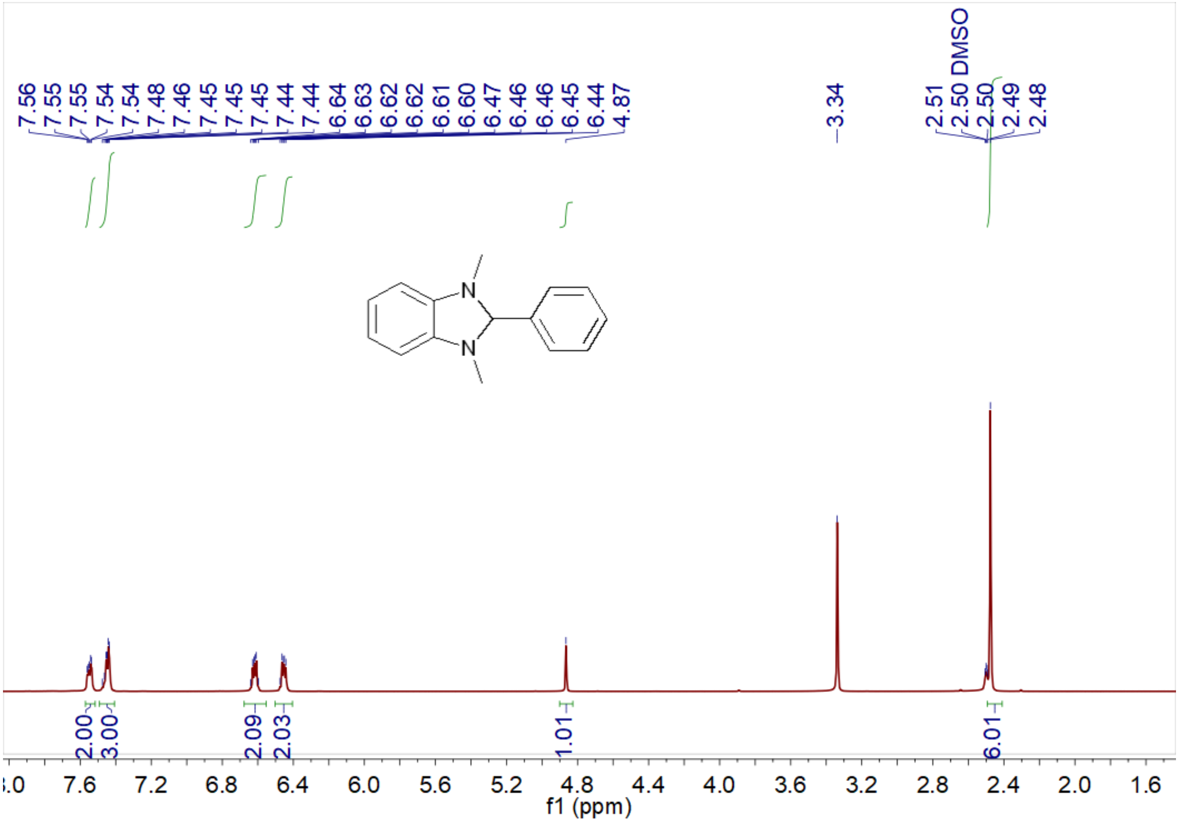

Supplementary Figure 84. ^{1}H NMR spectrum. ^{1}H NMR spectrum of BIH in DMSO-d_6.

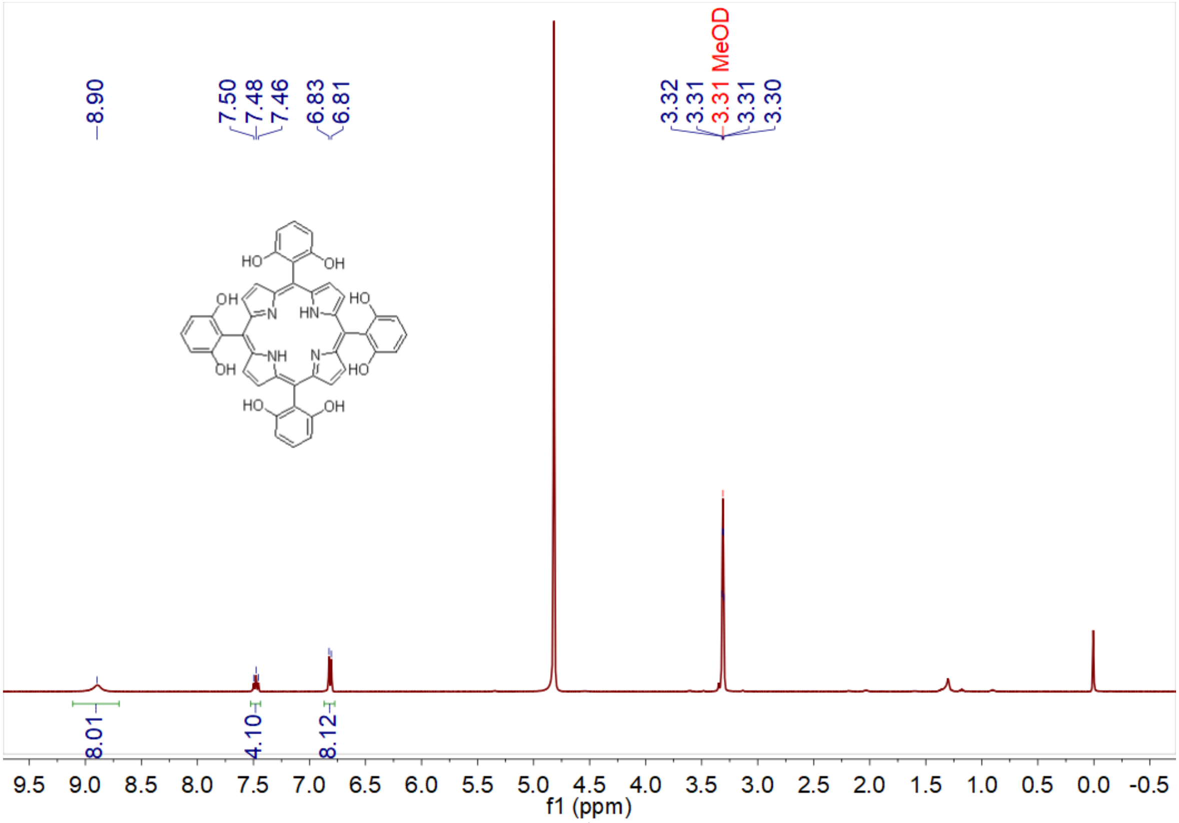

Supplementary Figure 85. ^{1}H NMR spectrum. ^{1}H NMR spectrum of TDHPP in methanol-d_4.

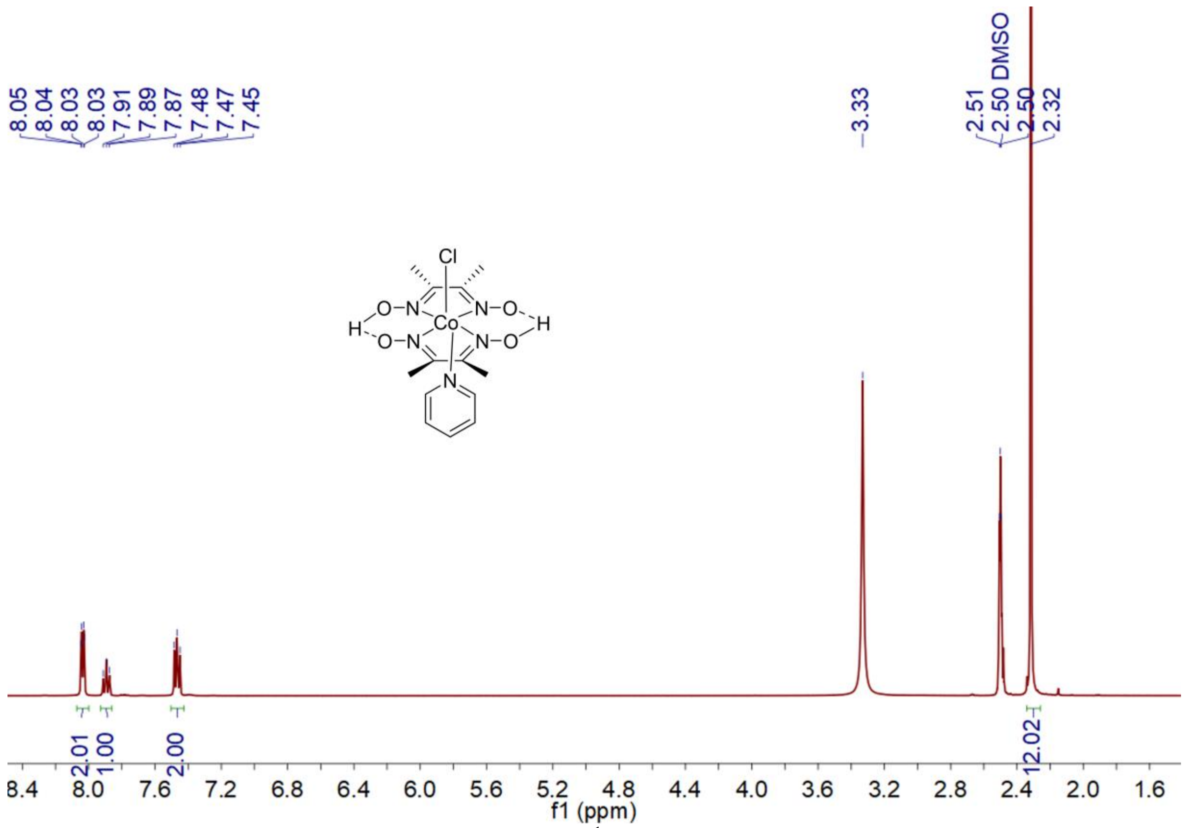

Supplementary Figure 86. ^{1}H NMR spectrum. ^{1}H NMR spectrum of Co(dmgH)$_2$PyCl in DMSO-d_6.

Supplementary References

1. Ming, M., *et al.* Efficient red-light-driven hydrogen evolution with an anthraquinone organic dye. *J. Am. Chem. Soc.* **144**, 19680-19684 (2022).
2. Irikura, M., Tamaki, Y. & Ishitani, O. Development of a panchromatic photosensitizer and its application to photocatalytic CO_2 reduction. *Chem. Sci.* **12**, 13888-13896 (2021).
3. Shipp, J., *et al.* Photocatalytic reduction of CO_2 to CO in aqueous solution under red-light irradiation by a Zn-porphyrin-sensitized Mn(I) catalyst. *Inorg. Chem.* **61**, 13281-13292 (2022).
4. Tamaki, Y., Koike, K., Morimoto, T., Yamazaki, Y. & Ishitani, O. Red-light-driven photocatalytic reduction of CO_2 using Os(II)–Re(I) supramolecular complexes. *Inorg. Chem.* **52**, 11902-11909 (2013).
5. Liang, L., *et al.* Efficient infrared light induced CO_2 reduction with nearly 100% CO selectivity enabled by metallic CoN porous atomic layers. *Nano Energy* **69**, 104421 (2020).
6. Shi, L., Ren, X., Wang, Q., Zhou, W. & Ye, J. Tridecaboron diphosphide: a new infrared light active photocatalyst for efficient CO_2 photoreduction under mild reaction conditions. *J. Mater. Chem. A* **9**, 2421-2428 (2021).
7. Wang, K., *et al.* Metallic $AgInS_2$ nanocrystals with sulfur vacancies boost atmospheric CO_2 photoreduction under near-infrared light illumination. *Appl. Catal. B Environ.* **332**, 122763 (2023).
8. Wu, W., *et al.* Red-light-driven CO_2 Photoreduction into CH_4 and CO enabled by narrow-gap conjugated microporous polymers. *Solar RRL* **7**, 2200907 (2023).
9. Yang, P., Wang, R., Zhou, M. & Wang, X. Photochemical construction of carbonitride structures for red-light redox catalysis. *Angew. Chem. Int. Ed.* **57**, 8674-8677 (2018).
10. Ye, L., *et al.* Synthesis of olive-green few-layered BiOI for efficient photoreduction of CO_2 into solar fuels under visible/near-infrared light. *Sol. Energy Mater. Sol. Cells* **144**, 732-739 (2016).
11. Tan, L., *et al.* Highly selective photoreduction of CO_2 with suppressing H_2 evolution over monolayer layered double hydroxide under irradiation above 600 nm. *Angew. Chem. Int. Ed.* **58**, 11860-11867 (2019).
12. Zeng, J.-Y., Wang, X.-S., Xie, B.-R., Li, Q.-R. & Zhang, X.-Z. Large π-conjugated metal–organic frameworks for infrared-light-driven CO_2 reduction. *J. Am. Chem. Soc.* **144**, 1218-1231 (2022).
13. Chaudhri, N., Brückner, C. & Zeller, M. Crystal structure of cis-7,8-dihydroxy-5,10,15,20-

tetraphenylchlorin and its zinc(II)–ethylenediamine complex. *Acta Crystallographica Section E Crystallographic Communications* **78**, 392-398 (2022).

14. Samankumara, L. P., Zeller, M., Krause, J. A. & Brückner, C. Syntheses, structures, modification, and optical properties of meso-tetraaryl-2,3-dimethoxychlorin, and two isomeric meso-tetraaryl-2,3,12,13-tetrahydroxybacteriochlorins. *Org. Biomol. Chem.* **8**, 1951 (2010).
15. Ho, P.-Y., *et al.* Light-driven reduction of CO_2 to CO in water with a cobalt molecular catalyst and an organic sensitizer. *ACS Catal.* **13**, 5979-5985 (2023).
16. Guo, Z., *et al.* Selectivity control of CO versus $HCOO^-$ production in the visible-light-driven catalytic reduction of CO_2 with two cooperative metal sites. *Nat. Catal.* **2**, 801-808 (2019).
17. Huckaba, A. J., Sharpe, E. A. & Delcamp, J. H. Photocatalytic reduction of CO_2 with Re-pyridyl-NHCs. *Inorg. Chem.* **55**, 682-690 (2015).
18. Li, Z., Wang, J.-W., Huang, Y. & Ouyang, G. Enhancing CO_2 photoreduction via the perfluorination of Co(II) phthalocyanine catalysts in a noble-metal-free system. *Chin. J. Catal.* **49**, 160-167 (2023).
19. Wang, J.-W., Jiang, L., Huang, H.-H., Han, Z. & Ouyang, G. Rapid electron transfer via dynamic coordinative interaction boosts quantum efficiency for photocatalytic CO_2 reduction. *Nat. Commun.* **12**, 4276 (2021).
20. Wang, J.-W., *et al.* Boosting CO_2 photoreduction by π–π induced preassembly between a Cu(I) sensitizer and a pyrene-appended Co(II) catalyst. *Proc. Natl. Acad. Sci. U.S.A.* **120**, e2221219120 (2023).
21. Nugegoda, D., Tzouras, N. V., Nolan, S. P. & Delcamp, J. H. N-Heterocyclic carbene gold complexes in a photocatalytic CO_2 reduction reaction. *Inorg. Chem.* **61**, 18802-18809 (2022).
22. Wang, J.-W., *et al.* Precious-metal-free CO_2 photoreduction boosted by dynamic coordinative interaction between pyridine-tethered Cu(I) sensitizers and a Co(II) catalyst. *JACS Au* **3**, 1984-1997 (2023).
